# An open-label, multiple ascending dose study of the anti-CTLA-4 antibody ipilimumab in viremic HIV patients

**DOI:** 10.1371/journal.pone.0198158

**Published:** 2018-06-07

**Authors:** Elizabeth Colston, Dennis Grasela, David Gardiner, R. Pat Bucy, Blisse Vakkalagadda, Alan J. Korman, Israel Lowy

**Affiliations:** 1 Innovative Medicines Development, Bristol-Myers Squibb, Princeton, New Jersey, United States of America; 2 Infectious Diseases Research and Development, GlaxoSmithKline, Collegeville, Pennsylvania, United States of America; 3 Department of Pathology, University of Alabama at Birmingham, Birmingham, Alabama, United States of America; 4 Clinical Pharmacology and Pharmacometrics, Bristol-Myers Squibb, Hopewell, New Jersey, United States of America; 5 Biologics Discovery California, Bristol-Myers Squibb, Redwood City, California, United States of America; 6 Translational Science and Clinical Oncology, Regeneron Pharmaceuticals, Tarrytown, New York, United States of America; Rush University, UNITED STATES

## Abstract

Expression of cytotoxic T-lymphocyte antigen 4 (CTLA-4), a negative regulator of T-cell function, is increased in chronic HIV-1 infection. It was hypothesized that CTLA-4 blockade may enhance immune response to HIV-1 and result in better control of viremia. This open-label, multiple ascending dose study (NCT03407105)—the first to examine ipilimumab in participants with HIV-1 infection—assessed the safety, tolerability, and pharmacokinetics of ipilimumab, as well as whether ipilimumab enhanced immune response to HIV-1 and improved control of viremia. Twenty-four participants received 2 or 4 doses of ipilimumab (0.1, 1, 3, or 5 mg/kg) every 28 days. No serious adverse events (AEs) or dose-limiting toxicities were reported; one participant discontinued ipilimumab for an AE of grade 2 facial palsy. Twenty participants (83.3%) had ≥1 AE; all but 1 were grade 1 or 2. Eight participants (33.3%) had potentially immune-related AEs (7 had grade 1 diarrhea not requiring corticosteroids; 1 who had diarrhea also had transient antinuclear antibody positivity; 1 had grade 2 facial palsy requiring corticosteroids). Two participants (8.3%), one each in the 0.1- and 1-mg/kg dose groups, had a decrease from baseline HIV-1 RNA of 0.85 and 1.36 log_10_ copies/mL. Fourteen participants (58.3%) had an increase from baseline HIV-1 RNA (mean, 0.87 log_10_ copies/mL; range, 0.59–1.29). Of these 14 participants, all but 1 were in the higher ipilimumab dose groups (3 or 5 mg/kg). No pattern was noted regarding change from baseline in CD4 or CD8 T cells; ex vivo assessments of immune response were precluded because of inadequate cell viability. Serum concentration data for ipilimumab showed biphasic disposition, with steady state reached by dose 3. Ipilimumab treatment was well tolerated and was associated with variations in HIV-1 RNA in excess of expected repeat measures in most participants, but these were not related to combination antiretroviral therapy status or CD4 counts. The mechanism(s) underlying the increased variation in HIV-1 RNA is unclear and needs further study.

## Introduction

Virus-induced suppression of host immunity contributes to the persistence of HIV. Combination antiretroviral therapy (cART) provides significant clinical benefits, transforming HIV into a chronic disease when treatment is available and patients are adherent to therapy [1]. Immune exhaustion and T-cell inactivation have been suggested as potential factors that contribute to ineffective immune responses and insufficient viral control seen in patients with HIV infection [1–3]. Broadly, the term immune “exhaustion” has been used to describe a loss of both effector (eg, cytokine secretion and cytotoxicity) and proliferative functions of T cells in response to antigen. In the case of chronic viral infection, including HIV, prolonged exposure to viral antigens leads to chronic stimulation and activation of T cells that may, in turn, trigger T-cell inactivation and immune exhaustion [2].

Despite the availability of cART, issues related to resistance, tolerability, toxicity, and drug–drug interactions remain; furthermore, while patients with HIV can achieve undetectable HIV RNA levels, immune dysfunction and inflammation persist [4]. A functional cure, defined as virologic control precluding the need for chronic cART, remains elusive. These considerations support the need for new therapeutic approaches for patients with HIV infection.

Cytotoxic T-lymphocyte antigen 4 (CTLA-4) is an immune checkpoint receptor that is upregulated on activated T cells and constitutively expressed by regulatory T cells. CTLA-4 binds B7-1 (cluster of differentiation [CD] 80 [CD80]) or B7-2 (CD86) and competitively blocks their ligation to CD28, thus preventing further T-cell activation. CTLA-4 is more highly expressed by unstimulated blood CD4 T cells from patients with HIV, regardless of treatment, compared with control T cells [5]. Increased CTLA-4 expression in HIV-infected patients was positively correlated with disease progression but negatively correlated with CD4 T-cell count and the ability of CD4 T cells to mount a response to viral antigen, as assessed by interleukin-2 production [6, 7]. Based on this evidence, blockade of CTLA-4 may be effective for patients with HIV infection [8].

Ipilimumab is a fully human monoclonal immunoglobulin G_1_κ antibody designed to block the CTLA-4 immune checkpoint to promote an effective immune response [9]. Ipilimumab was the first therapy proven to improve overall survival in patients with advanced melanoma. It was initially approved in the United States in 2011 for previously treated and treatment-naïve patients with unresectable or metastatic melanoma at a dose of 3 mg/kg every 3 weeks (× 4), and has subsequently been approved in more than 40 countries [10–12]. This study was the first clinical evaluation of ipilimumab in study participants with chronic HIV-1 infection, in the absence of concurrent malignancy, and was undertaken with the aim of investigating the safety, tolerability, and pharmacokinetics (PK) of ipilimumab in study participants infected with HIV-1, as well as to assess whether ipilimumab administration can enhance the immune response against HIV-1 and improve control of viremia.

## Materials and methods

### Ethics statement

Written informed consent was obtained from all participants. The study was approved by the institutional review board responsible for the 3 study sites: Western Institutional Review Board (Olympia, WA, USA). The study was conducted in compliance with the Declaration of Helsinki, Good Clinical Practice guidelines, and local regulatory requirements. Registration was not required at the time this phase 1 study was initiated; thus, this study was retrospectively registered on ClinicalTrials.gov (NCT03407105). All future clinical trials will be prospectively registered because the sponsor is committed to registering all phase 1–4 interventional trials, conducted in any geographic location, on applicable registries, prior to the first patient being enrolled. A copy of the study protocol has been included in the S2 Appendix.

### Study design and participants

HIV-infected participants were enrolled into this phase 1, open-label, partially randomized, multiple ascending dose study of ipilimumab at 3 centers in the United States (Quest Clinical Research, San Francisco, CA, USA; Tower ID Medical Associates, Los Angeles, CA, USA; and Care Resource, Miami, FL, USA). The study was designed primarily to determine the safety and tolerability of ipilimumab administered intravenously every 28 days for 2 or 4 doses in escalating dose cohorts of participants with chronic HIV-1 infection with no concurrent malignancy, for whom virus was no longer suppressed by available and tolerable cART. Secondary objectives included characterization of the PK of ipilimumab, assessment of any clinical activity to augment an effective immune response against HIV-1, and assessment of changes in cellular and humoral immune responses toward HIV-1, *Candida*, and a tetanus booster following administration of ipilimumab.

Eligible participants were adult (aged ≥18 years) males or females with chronic HIV-1 infection who had detectable viremia. In the 3 months before the study, HIV-1 RNA measurements (determined by reverse transcriptase–polymerase chain reaction) were required to be between 1000 and 100,000 copies/mL on at least 2 occasions, with the measurements within 0.5 log_10_ copies/mL variation of each other (including screening value and 1 value ≥45 days before screening). In the 6 months before the study, CD4 counts were required to be ≥100 cells/mm^3^ on at least 2 determinations (including screening value and 1 value ≥90 days before screening).

Eligible participants had undergone at least 2 previous changes in their cART regimen for documented virologic failure (breakthrough viremia while adherent to a regimen containing ≥3 antiretroviral agents, and exclusive of prior monotherapy or dual therapy) and documented resistance tests demonstrating ≥1 mutation to each major therapeutic class of antiretroviral therapy, including nucleoside reverse transcriptase inhibitors, non-nucleoside reverse transcriptase inhibitors, and protease inhibitors. If a therapeutic class of agent was not being used for reasons of intolerance, then documentation of the signs of intolerance could have substituted for resistance mutations to that class.

For the initial 3 cohorts (ipilimumab 0.1 mg/kg, 1 mg/kg, and 3 mg/kg; all for 2 doses), eligible participants were on stable cART for at least 3 months. For the final 2 cohorts (ipilimumab 3 mg/kg for 4 doses and 5 mg/kg for 2 doses), a protocol amendment broadened the inclusion criteria to include participants who were no longer taking previously failed cART due to intolerance or regimen fatigue.

Eligible participants had to be free of any significant organ compromise or functional medical disorder and willing to remain on their current therapy for the 12-week study duration (unless toxicity or safety issues indicated otherwise). Eligible participants had hemoglobin ≥8 g/dL, white blood cell count ≥2000 cells/mm^3^, neutrophil count ≥750 cells/mm^3^, platelet count ≥100,000 cells/mm^3^, alanine aminotransferase/aspartate aminotransferase/alkaline phosphate all ≤2 × upper limit of normal, and proteinuria <1 g/day. Sexually active males agreed to use an acceptable barrier method for birth control and prevention of venereal disease (by chance, no female participants were enrolled in the study).

Participants were excluded if they had received any experimental treatment within 4 months before screening or had initiated any new medications within 4 weeks before screening that might reasonably have affected immune response or HIV-1 RNA. Other exclusion criteria included a tetanus booster within 2 months before screening or history of reaction to tetanus vaccine; history of autoimmune disease at risk for recurrence; current malignancy except for stage A or B cervical or basal cell carcinoma; chronic viral hepatitis due to hepatitis B virus or hepatitis C virus (HCV; undergoing current treatment with interferon or ribavirin, or with hepatitis B virus DNA HIV-1 RNA >25 pg/mL or HCV RNA HIV-1 RNA >20,000 IU/mL); current treatment or prophylaxis for tuberculosis infection; chronic active infectious disease (other than HIV) such as chronic renal infection; chronic chest infection with bronchiectasis or sinusitis; and any known active drug or alcohol abuse that would interfere with participation in the study.

Groups of between 3 and 6 participants were treated at each of 5 ipilimumab dose levels or dosing regimens. Ipilimumab was administered once every 28 days by intravenous infusion over 90 minutes. For the ipilimumab 0.1-mg/kg, 1-mg/kg, and 3-mg/kg dose levels (all 2 doses), participants were assigned to the cohort available in the dose-escalation scheme when they presented to the investigative site. The maximum tolerated dose (MTD) was defined as the highest dose at which ≤1 of 6 participants experienced a dose-limiting toxicity (DLT). When an MTD was not established for the 6 participants administered ipilimumab at 3 mg/kg for 2 doses, a protocol amendment allowed additional participants (6 per group) to be randomly assigned to receive either 3 mg/kg (for 4 doses) or 5 mg/kg (for 2 doses). These final 2 cohorts were studied simultaneously with open-label, central randomization.

During the screening phase, study visits consisted of a prestudy visit (within 28 days before infusion of study drug) and a pre-entry visit (7 days before first infusion of study drug). During the treatment phase, participants were given infusions of ipilimumab every 28 days for 2 or 4 doses and were required to visit the investigator’s office or clinic every 3 to 7 days for PK sampling, vital sign measurements, physical examinations, clinical laboratory testing, HIV-1 RNA and immunologic assessments, and/or the collection of adverse event (AE) data. All participants received a tetanus booster on day 29 of the study and at the end of the observation period following the second dose of ipilimumab. Participants receiving 2 or 4 doses were followed until day 85 or 141, respectively. No further long-term follow-up was performed.

### End points and assessments

Safety evaluations included the assessment of the frequency and severity of treatment-emergent AEs, concomitant medications, physical examinations, vital sign measurements, electrocardiograms, chest radiography, clinical laboratory tests (including serology, hematology, clinical chemistry, and urinalysis), plasma sampling for immunogenicity measurements, and flow cytometry for evaluation of T-cell subsets. Tolerability was determined by evaluation of AEs, drug-induced DLTs, and observation for MTD. DLTs were defined as grade ≥3 AEs or new laboratory abnormalities occurring during or after the 28-day infusion period (up to 1 month posttreatment) that were considered at least possibly related to ipilimumab and did not resolve (reduce to grade 2 or less) within 48 hours with appropriate treatment (including corticosteroids if necessary); grade 3 infusion reactions that resolved within 2 hours with appropriate therapy were excluded. Clinical activity evaluations included assessments of HIV-1 RNA and CD4 T-cell counts from baseline to protocol-designated evaluation time points. The primary clinical activity parameter was HIV-1 RNA assessment. HIV-1 RNA was determined by reverse transcriptase–polymerase chain reaction using the Roche COBAS Amplicor HIV-1 Monitor Test, version 1.5 (Roche Diagnostics, Indianapolis, IN, USA). In an individual participant, a change from baseline HIV-1 RNA of at least 0.5 log_10_ copies/mL sustained over 2 or more consecutive determinations was considered significant and to indicate potential clinical activity. The secondary clinical activity parameter was CD4 T-cell count. Lymphocyte subpopulations were measured using AIDS Clinical Trials Group consensus methods that used fluorochrome-labeled monoclonal antibodies (BD Pharmingen, San Diego, CA, USA) to the following antigens: CD3, CD4, CD8, CD28, CD38, CD45RA, CD62L, and HLADR; of these, only CD4 and CD8 were analyzed. In an individual participant, an increase or decrease from baseline CD4 count by more than 30% sustained over the course of 2 or more consecutive determinations during the trial was considered significant and to indicate potential clinical activity.

Evaluation of immunologic activity included measurement of changes in the frequency and amount of CD4 and CD8 T cells with cytokine responses to HIV-1, *Candida*, and tetanus antigens; changes in lymphocyte proliferation assay to HIV-1, *Candida*, and tetanus antigens; and changes in anti-tetanus toxin antibody levels.

For the lymphocyte proliferation assay, the AIDS Clinical Trials Group consensus method was used [13]. Briefly, blood specimens were shipped by overnight courier to a central laboratory and 100,000 peripheral blood mononuclear cells inoculated per well of a 96-well plate were co-cultured with HIV-1 antigens including p24 antigen, Gag peptide pool, and a mixture of other HIV peptides [13]. Positive controls were *staphylococcus* enterotoxin B superantigen (10 μg/mL), pokeweed mitogen (0.1 μg/mL), tetanus toxoid (1 LFU/mL), *Candida albicans* (10 μg/mL), and cytomegalovirus antigen. The negative control was media alone. Following 6 days at 37°C in a 5% CO_2_ humidified incubator, the plates were pulsed with 25 μL/well of [3H]thymidine (1 μCi/well; NEN Life Science Products, Boston, MA, USA). Cells were harvested 6 hours later on glass fiber filters using a cell harvester. A scintillation counter measured the amount of radioactivity incorporated into DNA. The results are reported as stimulation indices (counts per minute experimental/counts per minute background unstimulated).

The cytokine assay for production of interferon (IFN)-γ was done using HIV-1 antigens including p24 antigen, Gag peptide pool, and a mixture of other HIV peptides [13]. Positive controls were *staphylococcus* enterotoxin B superantigen (10 μg/mL) and tetanus toxoid (1 LFU/mL). The negative control was media alone. One million peripheral blood mononuclear cells were co-cultured with antigen in a 96-well plate at 37°C in a 5% CO_2_ humidified incubator for 48 hours. From each well, 150 μL of supernatant was placed in a separate, labeled 96-well plate and stored at 20°C. IFN-γ was measured in the supernatant using a commercial enzyme immunoassay (Caltag, Buckingham, UK) in a single batch for each participant.

To assess PK parameters, plasma concentrations of ipilimumab were measured using a validated method. In participants receiving 2 doses of ipilimumab, PK sampling was performed on days 1 and 29 at 0.5 hours before infusion and 2 and 4 hours after the start of infusion, and on days 4, 8, 15, 22, 32, 36, 43, 57, and 85. In participants receiving 4 doses of ipilimumab, PK sampling was performed on days 1, 29, 57, and 85 at 0.5 hours before infusion and 2 and 4 hours after the start of infusion, and on days 4, 8, 15, 22, 32, 36, 43, 53, 64, 71, 78, 92, 99, 106, 113, and 141. Individual participant PK parameter values were derived by a noncompartmental method, using a validated PK analysis program (Phoenix WinNonlin version 6.3, Pharsight Corporation, Mountain View, CA, USA). Maximum plasma concentration observed postdose (C_max_), minimum plasma concentration observed postdose (C_min_), time of C_max_, area under the concentration–time curve over a dosing interval (AUC_tau_), and clearance at steady state were reported.

### Statistical methods

The sample size of up to 50 participants was based on the trial design for dose escalation and safety evaluation requirements, which allowed for up to 30 participants (3 to 6 participants per cohort) at 5 dose levels or dose regimens and an additional 20 participants in 2 cohorts for evaluation of clinical activity. All participants qualified for both the safety population and the clinical activity population; therefore, analyses were based on all participants, except where data for the 4-dose cohort and all the 2-dose cohorts were analyzed separately. Descriptive statistics were used to summarize safety data, antiviral activity, immunologic assessments, and PK data. Analyses were performed using SAS software (SAS Institute Inc., Cary, NC, USA) version 8.2 or higher.

### Data visualization

A data quality check was performed via independent review of all data by 2 individuals. The software program TIBCO Spotfire version 4.5.0 (TIBCO Software Inc., Palo Alto, CA, USA) was utilized for data visualization and production of some figures.

## Results

### Participant disposition and baseline characteristics

**Fig 1:**
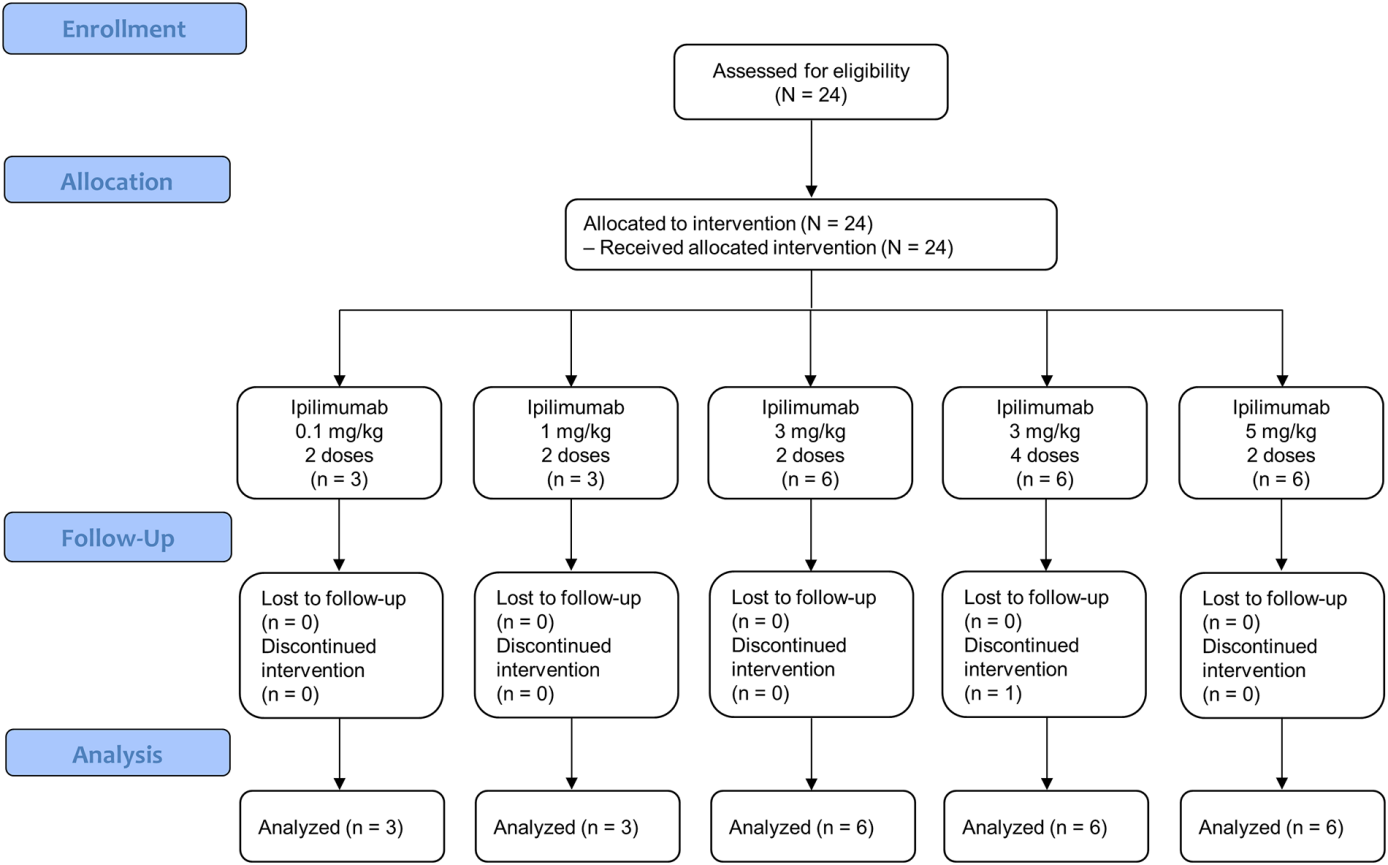
Participant disposition.

A total of 24 participants were enrolled, treated, and completed this study, which was conducted from April 21, 2003 to February 21, 2006. All 24 participants (100.0%) were included in analyses of safety, antiviral and immunologic outcomes, and PK data (Fig 1). The ipilimumab cohorts were as follows: 0.1 mg/kg for 2 doses (n = 3); 1 mg/kg for 2 doses (n = 3); 3 mg/kg for 2 doses (n = 6); 3 mg/kg for 4 doses (n = 6); and 5 mg/kg for 2 doses (n = 6).

There were no clinically relevant differences in the baseline demographic characteristics of participants in the various cohorts (Table 1). The average age of participants was 46.5 years, all participants were male, and most were white. The cohorts were generally similar regarding mean time since HIV-1 diagnosis, mean HIV-1 RNA at screening, and the number of prior failed cART regimens. Mean CD4 T-cell count at screening was numerically higher in the 5-mg/kg group, while the counts at screening were generally similar for the other dose groups. As a result of the study amendment, the cohorts were unbalanced with respect to the number of participants on cART, with the 3-mg/kg 4-dose and 5-mg/kg cohorts including some participants who were not on cART.

**Table 1:** Baseline participant characteristics.

Characteristic	0.1 mg/kg 2 doses (n = 3)	1 mg/kg 2 doses (n = 3)	3 mg/kg 2 doses (n = 6)	3 mg/kg 4 doses (n = 6)	5 mg/kg 2 doses (n = 6)	Total (N = 24)
Mean age, years (range)	47.7 (38–54)	48.7 (42–56)	46.2 (40–53)	46.7 (40–61)	44.8 (38–50)	46.5 (38–61)
Male sex, n (%)	3 (100.0)	3 (100.0)	6 (100.0)	6 (100.0)	6 (100.0)	24 (100.0)
Race, n (%)						
White	2 (66.7)	2 (66.7)	5 (83.3)	6 (100.0)	4 (66.7)	19 (79.2)
Black	0	0	0	0	1 (16.7)	1 (4.2)
Hispanic	0	1 (33.3)	1 (16.7)	0	1 (16.7)	3 (12.5)
Other	1 (33.3)	0	0	0	0	1 (4.2)
Mean time since diagnosis, years (range)	15.0 (12.8–16.0)	15.7 (13.4–18.3)	17.5 (14.6–21.8)	11.3 (4.8–18.4)	18.1 (10.4–22.6)	16.5 (4.8–22.6)
Mean HIV-1 RNA at screening, log_10_ copies/mL (range)	4.25 (3.62–4.64)	4.58 (4.35–4.80)	3.94 (3.08–4.97)	4.64 (3.62–5.11)	4.26 (3.58–4.72)	4.32 (3.08–5.11)
Mean CD4 count at screening, cells/μL (range)	373.3 (146–742)	309.3 (171–468)	349.3 (180–610)	340.2 (189–570)	441.5 (251–785)	368.1 (146–785)
Number of prior failed antiretroviral regimens, n	2	2	2	2	2	2
On antiretroviral therapy, n						
Yes	3	3	6	1	4	17
No	0	0	0	5	2	7

### Safety and tolerability

The primary objective of the study was to examine the safety and tolerability of ipilimumab in HIV-1-infected participants. There were no deaths or serious AEs. No DLTs were observed. There was one discontinuation due to an AE: moderate (grade 2) facial palsy in a participant in the 3-mg/kg 4-dose cohort—the participant had a history of herpes simplex, herpes zoster, and cytomegalovirus retinitis. The participant received ipilimumab 3 mg/kg on days 1 and 29. On day 55, the participant was noted to have mild (grade 1) facial palsy. The investigator determined that no treatment for the facial palsy was required, and the third dose of ipilimumab was administered on day 57. On day 58, the facial palsy worsened to moderate (grade 2) severity. On day 60, treatment was initiated with oral prednisone (20 mg 3 times daily for 3 days, 20 mg twice daily for 3 days, and 20 mg once daily for 3 days). Due to the facial palsy, the fourth ipilimumab dose on day 85 was withheld, but the participant continued to fully participate in all protocol-defined visits and assessments. A diagnostic workup for the facial palsy was not performed. The AE of facial palsy was considered by the investigator to be possibly related to the study drug. At study end, the moderate (grade 2) facial palsy was reported as continuing.

During the study, 20 of 24 participants (83.3%) reported at least one AE of any grade (Table 2). The most frequent AEs were diarrhea, nasopharyngitis, lymphadenopathy, and sinus headache, and there was no suggestion of dose-related differences in frequency. All events were mild or moderate in severity (grade 1 or 2), with the exception of a severe (grade 3) event of neutropenia in a participant in the 0.1-mg/kg cohort. The participant had mild neutropenia, anemia, and thrombocytopenia at baseline and mild neutropenia at most study assessment time points. Baseline neutrophil count was 1.0 × 10^9^/L (corresponding to a grade 2 decrease with lower limit of normal, 1.8 × 10^9^/L) and, on day 91, the participant experienced grade 3 neutropenia (neutrophil count, 0.7 × 10^9^/L). There were no concurrent AEs suggestive of infection, and simultaneous hemoglobin and platelet count values were at or near the participant’s baseline values. The grade 3 neutropenia was considered unlikely to be related to ipilimumab and resolved without treatment. It was the only AE with a severity greater than grade 2 in the study.

**Table 2:** Adverse events reported in ≥4% of participants (total).

Adverse event, n (%)	0.1 mg/kg 2 doses (n = 3)	1 mg/kg 2 doses (n = 3)	3 mg/kg 2 doses (n = 6)	3 mg/kg 4 doses (n = 6)	5 mg/kg 2 doses (n = 6)	Total (N = 24)
Any adverse event						
Grade 1	1 (33.3)	1 (33.3)	3 (50.0)	1 (16.7)	3 (50.0)	9 (37.5)
Grade 2	1 (33.3)	1 (33.3)	3 (50.0)	4 (66.7)	1 (16.7)	10 (41.7)
Grade 3	1 (33.3)	0	0	0	0	1 (4.2)
Total	3 (100.0)	2 (66.7)	6 (100.0)	5 (83.3)	4 (66.7)	20 (83.3)
Diarrhea	2 (66.7)	1 (33.3)	1 (16.7)	1 (16.7)	2 (33.3)	7 (29.2)
Lymphadenopathy	1 (33.3)	0	2 (33.3)	0	0	3 (12.5)
Nasopharyngitis	0	1 (33.3)	1 (16.7)	1 (16.7)	0	3 (12.5)
Sinus headache	0	0	2 (33.3)	1 (16.7)	0	3 (12.5)
Areflexia	0	1 (33.3)	1 (16.7)	0	0	2 (8.3)
Back pain	1 (33.3)	1 (33.3)	0	0	0	2 (8.3)
Cough	0	1 (33.3)	1 (16.7)	0	0	2 (8.3)
Dehydration	0	0	1 (16.7)	0	1 (16.7)	2 (8.3)
Dizziness	0	0	2 (33.3)	0	0	2 (8.3)
Herpes simplex	0	1 (33.3)	0	1 (16.7)	0	2 (8.3)
Nausea	0	0	1 (16.7)	1 (16.7)	0	2 (8.3)
Pharyngolaryngeal pain	1 (33.3)	0	1 (16.7)	0	0	2 (8.3)
Sinus congestion	0	0	0	1 (16.7)	1 (16.7)	2 (8.3)

Potentially immune-related AEs, defined as clinically significant events consistent with an immune-mediated mechanism, were identified in 8 participants. One of the 8 participants had grade 2 facial palsy (described earlier). In 7 participants, the potential immune-related AE was diarrhea, all were grade 1 in severity, none resulted in dose limitation, and none required treatment with corticosteroids. Diarrhea occurred in 2 participants in the 0.1-mg/kg cohort; 1 participant in the 1-mg/kg cohort; 1 participant in the 3-mg/kg 2-dose cohort; 1 participant in the 3-mg/kg 4-dose cohort; and 2 participants in the 5-mg/kg cohort who each had 2 episodes of diarrhea. Two participants required treatment for the diarrhea: 1 participant in the 0.1-mg/kg cohort who had a history of diarrhea received diphenoxylate/atropine, which was continued until study completion, and 1 participant in the 5-mg/kg cohort required a 7-day course of loperamide for the second episode of diarrhea. With the exception of 1 participant in the 0.1-mg/kg cohort with onset of diarrhea 1 day before the final study visit, all events of diarrhea had resolved by the end of the study. One of the participants with diarrhea also had transient antinuclear antibody (ANA) positivity considered possibly related to ipilimumab. This participant had a past medical history of asthma, psoriasis, and vitiligo. Their baseline ANA titer was <1:40 (negative), day 29 ANA titer was 1:160, speckled pattern (≥4-fold increase vs baseline), and day 57 ANA titer was <1:40 (negative). Throughout the study there was no report of rash or physical examination abnormality for this participant, and their hematology and chemistry laboratory values were within normal limits. Autoimmune test results for rheumatoid factor, thyroglobulin antibody, and cardiolipin antibody were negative.

Finally, AEs in the infections and infestations category were observed in 10 of 24 participants (41.7%) and included nasopharyngitis, herpes simplex virus outbreak, amoebic dysentery, bacteriuria, body tinea, candidiasis, folliculitis, giardiasis, gingival infection, onychomycosis, oral candidiasis, otitis externa, sinusitis, upper respiratory infection, and urinary tract infection (data not shown). There were no AEs to suggest an immune reconstitution syndrome. Infection-related AEs suggested neither treatment-related immune deficits nor exaggerated immune responses during the observation period.

### Clinical activity

A secondary study objective was to evaluate the clinical activity of ipilimumab in HIV-1-infected participants; evaluations included measurements of HIV-1 RNA and CD4 cell count from baseline to protocol-specified time points.

**Fig 2:**
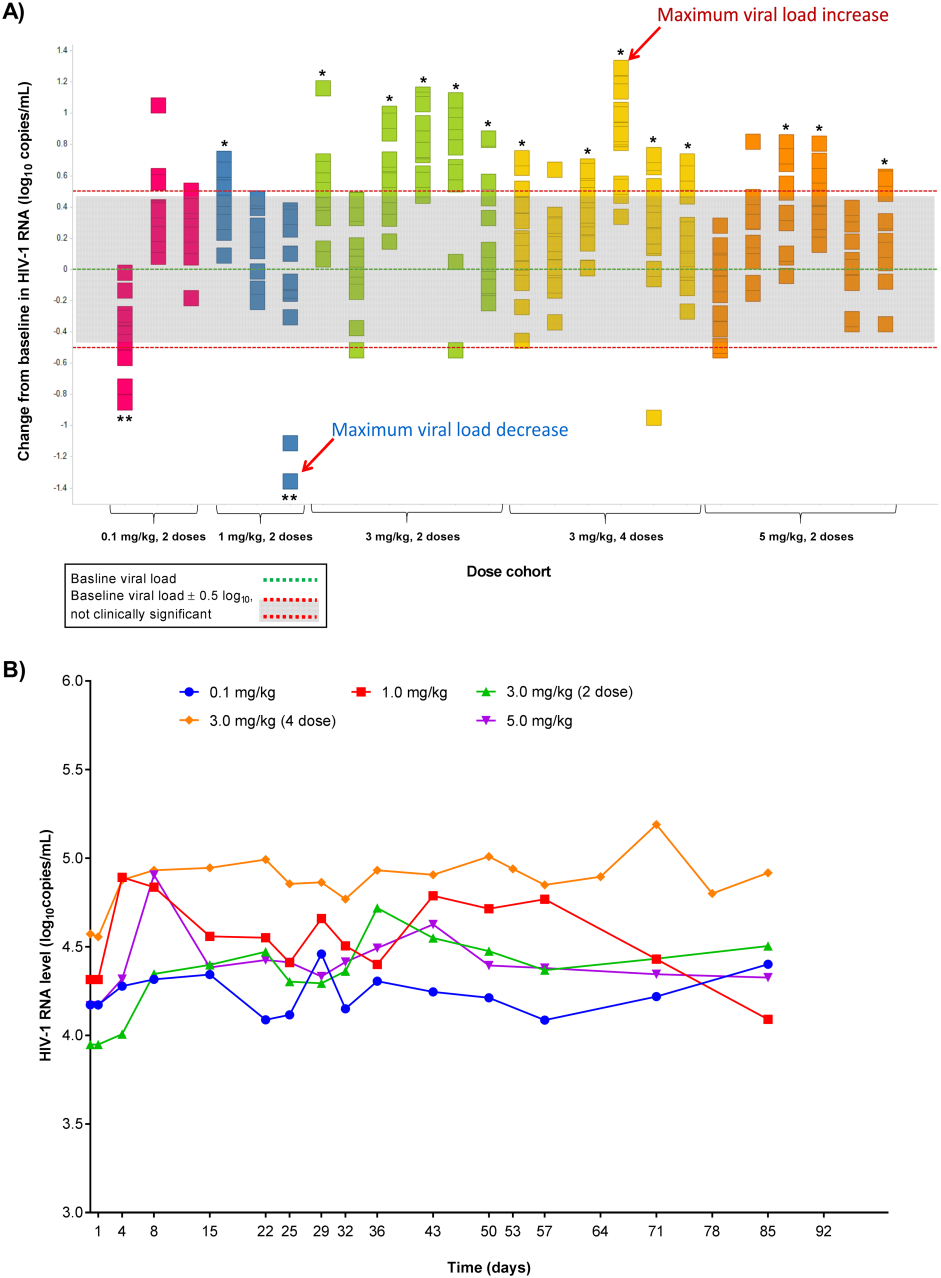
HIV-1 RNA change from baseline. **(A)** HIV-1 RNA values for individual participants (individual squares arranged vertically represent test results from a given study day for a given participant; the 2 participants with maximum HIV-1 RNA increase and decrease, respectively, are indicated with arrows; the 14 participants with significant increases in HIV-1 RNA are indicated with asterisks; the 2 participants with significant decreases in HIV-1 RNA are indicated with double asterisks); **(B)** mean HIV-1 RNA values over time for each dose cohort.

Eight of 24 participants (33.3%) did not have a significant change in HIV-1 RNA, defined as a ≥0.5 log_10_ copies/mL change in HIV-1 RNA sustained over 2 or more consecutive measurements. Two of 24 participants (8.3%) had a significant decrease in HIV-1 RNA of 0.85 and 1.36 log_10_ copies/mL, respectively (Fig 2A). Both participants with a decrease in HIV-1 RNA were in the lower dose cohorts (0.1 and 1 mg/kg). Fourteen of 24 participants (58.3%) had a significant increase in HIV-1 RNA (mean, 0.87 log_10_ copies/mL; range, 0.59–1.29) (Fig 2A). Of these 14 participants, all but 1 were in the higher dose groups; of these, 5 were in the 3-mg/kg 2-dose cohort, 5 were in the 3-mg/kg 4-dose cohort, and 3 were in the 5-mg/kg dose cohort (Fig 2A). Six of the 14 participants with an increase in HIV-1 RNA were not on cART during the study. Among these 6 participants, the mean (range) HIV-1 RNA increase from baseline was 0.80 (0.59–1.29) log_10_ copies/mL. By comparison, among the 8 of 14 participants who experienced an increase in HIV-1 RNA and were on cART during the study, the mean (range) increase from baseline HIV-1 RNA was 0.93 (0.71–1.16) log_10_ copies/mL. Of the 14 participants with HIV-1 RNA increase, 8 had an increase as soon as day 14 after the first dose (of these 8, 1 was in the 1-mg/kg dose cohort, 3 were in the 3-mg/kg 2-dose cohort, 1 was in the 3-mg/kg 4-dose cohort, and 2 were in the 5-mg/kg dose cohort; of these 8, 3 were not on cART and 5 were on cART; data not shown). Mean HIV-1 RNA values over time were lowest for the 0.1-mg/kg dose cohort and highest for the 3-mg/kg 4-dose cohort (Fig 2B).

**Fig 3:**
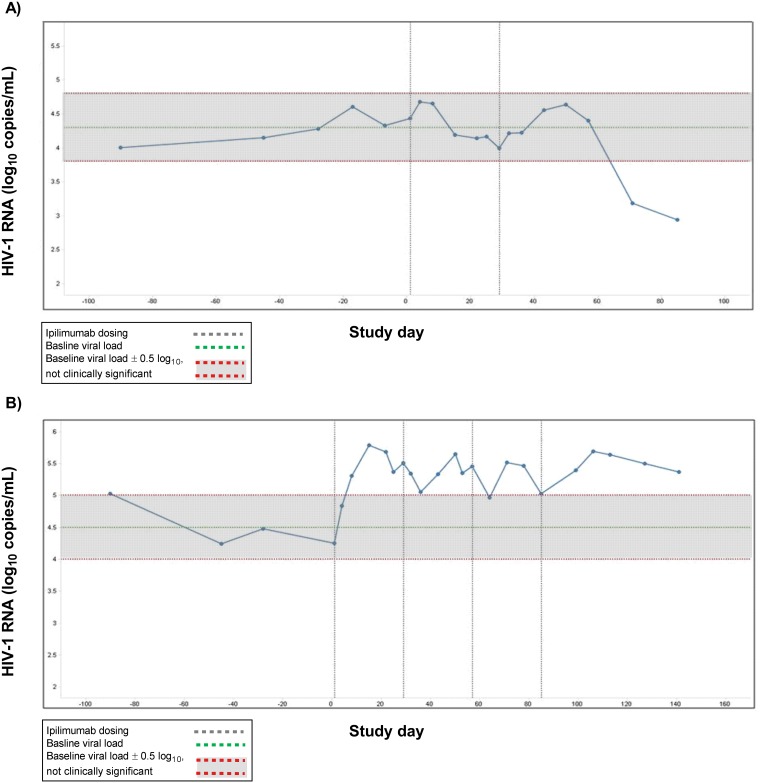
Data for the participants with the maximum increase or decrease in HIV-1 RNA from baseline. **(A)** Participant A (ipilimumab 1 mg/kg, 2 doses); **(B)** Participant B (ipilimumab 3 mg/kg, 4 doses).

Participant A had the maximum HIV-1 RNA decrease from baseline in the study (−1.36 log_10_ copies/mL) on the last visit day on day 85 (Fig 3A). This participant had received 2 previous cART regimens and was on a stable cART regimen during the study. The mean predose baseline HIV-1 RNA was 21,990 copies/mL (4.34 log_10_ copies/mL) and mean predose baseline CD4 count was 304 cells/μL. Participant A received ipilimumab 1 mg/kg on days 1 and 29 and experienced an HIV-1 RNA decrease of ≥0.5 log_10_ copies/mL on days 71 and 85 (Fig 3A). This participant achieved a significant HIV-1 RNA decrease 42 days after the second dose of ipilimumab. No follow-up information is available about the durability of HIV-1 RNA decrease beyond the study time points shown. During the study, the participant’s CD4 count change from baseline ranged from a decrease of 45 cells/μL to an increase of 43 cells/μL; during the period of HIV-1 RNA decrease, the CD4 count was 342 cells/μL on day 71 and 264 cells/μL on day 85; no pattern was noted regarding change from baseline in CD8 count.

Participant B had the maximum HIV-1 RNA increase from baseline in the study (+1.29 log_10_ copies/mL). This participant had received 2 previous cART regimens and was not on a stable cART regimen during the study. The mean predose baseline HIV-1 RNA was 43,472 copies/mL (4.64 log_10_ copies/mL) and mean predose baseline CD4 count was 503 cells/μL. Participant B received 4 doses of ipilimumab 3 mg/kg on days 1, 29, 57, and 85 and experienced sustained HIV-1 RNA increases from days 8 to 141 (Fig 3B). No follow-up information about the durability of HIV-1 RNA increase is available for this participant beyond day 141. During the study, the participant’s CD4 count change from baseline ranged from a decrease of 150 cells/μL to an increase of 312 cells/μL; during the period of HIV-1 RNA increase from days 8 to 141, the CD4 count ranged from 454 to 815 cells/μL. No pattern was noted regarding change from baseline in CD8 count.

**Fig 4:**
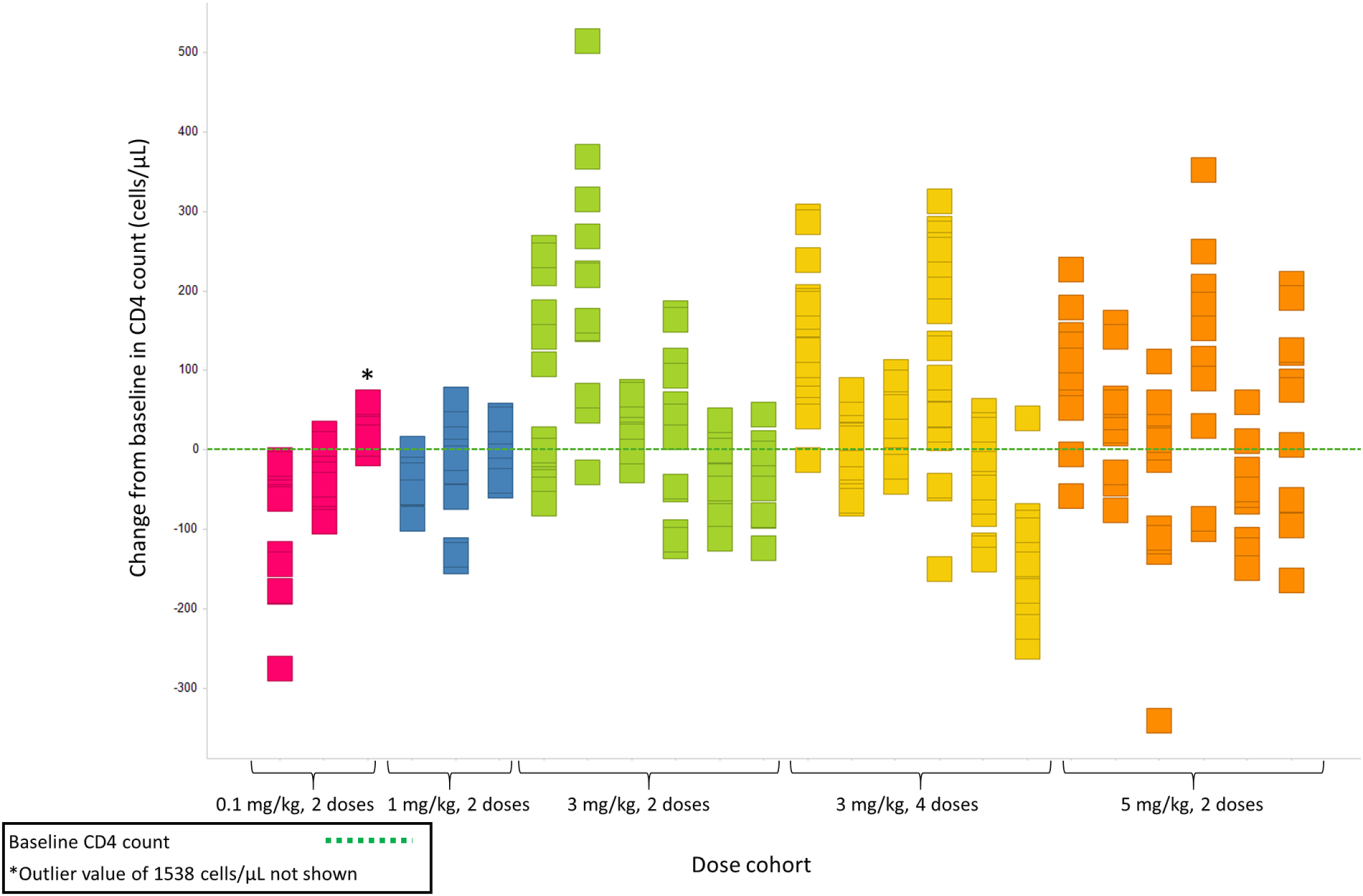
CD4 count change from baseline. Individual squares arranged vertically represent test results from a given study day for a given participant.

Ten of 24 participants (41.7%) experienced no significant change in CD4 count. Four of 24 participants (16.7%) experienced a decrease in CD4 count, and 10 of 24 (41.7%) experienced an increase in CD4 count (Fig 4). Of the 10 participants with an increase in CD4 count, 9 (90.0%) were in the higher dose cohorts (4 were in the 3-mg/kg 2-dose cohort, 2 were in the 3-mg/kg 4-dose cohort, and 3 were in the 5-mg/kg dose cohort) (data not shown). Generally, observed increases in CD4 count did not correspond with similar magnitude changes in the percentage of CD4 cells, were not appreciably above the level of variation observed during the screening period, were transient, did not appear to be dose-related, and did not correlate with changes in HIV-1 RNA.

Mean CD8 cell counts were within normal limits in the 1-mg/kg cohort and were above normal limits in the 0.1-mg/kg cohort at most evaluation time points; above normal limits in the 3-mg/kg 2-dose cohort and the 5-mg/kg cohorts at all evaluation time points; and showed no discernible pattern in the 3-mg/kg 4-dose cohort.

### Immunologic outcomes

Another secondary study objective was to assess cellular and humoral immune responses to HIV-1, *Candida*, and tetanus booster following ipilimumab infusion. Changes in lymphocyte proliferation and cytokine induction and secretion assays in response to HIV-1, *Candida*, and tetanus antigens were minimal and similar across cohorts (data not shown). Similarly, changes in anti-tetanus toxoid antibody levels were minimal and not considered meaningful over time. Examination of mean data by cohort did not show any apparent treatment- or dose-related trends in serum levels of neopterin, high-sensitivity C-reactive protein, tumor necrosis factor-α, soluble tumor necrosis factor receptor II, or interleukin-2 soluble receptor. Examination of participant-specific data from these immunologic assays did not show any clear trends or patterns in participants with a significant change in either HIV-1 RNA or CD4 count. T-cell activation marker analysis, T-cell subset analysis, and enzyme-linked immunospot assays were not performed.

### Pharmacokinetics

The serum concentration data of ipilimumab showed biphasic disposition. In the 3-mg/kg 4-dose regimen in HIV-1-infected participants, the geometric mean (% coefficient of variation) values for C_max_, C_min_, AUC_tau_, and clearance at steady state of ipilimumab following dose 4 were 51.8 (30.6) μg/mL, 6.29 (37.4) μg/mL, 13,400 (38.3) μg·h/mL, and 0.22 (48.7) mL/h/kg, respectively. Steady-state concentrations appear to have been reached by dose 3. In the 2-dose regimen, ipilimumab concentrations increased with increasing dose, but it was not possible to determine whether the increase was dose-proportional at steady state because steady state was not reached over this dosing period. In both the 2- and 4-dose regimens, the sampling time was too short for an accurate estimation of half-life. Finally, plotting AUC_tau_, C_max_, and C_min_ (dose normalized and not dose normalized; for the doses for which there were data) versus baseline CD4 values for participants on cART, as well as for those not on cART, did not reveal any meaningful relationship.

## Discussion

This study, undertaken during the early days of clinical development of ipilimumab, was the first clinical evaluation of ipilimumab in study participants with chronic HIV-1 infection, and in whom virus was no longer suppressed by available and tolerable cART. The primary objective was to establish the safety and tolerability of ipilimumab. A secondary objective was to determine whether CTLA-4 blockade augmented an effective immune response to HIV-1, resulting in clinical activity as evidenced by reduction in HIV-1 RNA. It was hypothesized that therapeutic blockade of CTLA-4 may enhance study participants’ immune response to HIV-1, allowing better control of viremia; however, consideration was given to the potential that drug-induced activation of CD4 T cells may result in enhanced viral replication, increases in HIV-1 RNA, and decreases in CD4 T-cell counts.

This study demonstrated that treatment with ipilimumab was safe and tolerable across the dose range and time frame evaluated. No participant experienced a serious AE, and there were no deaths during the study; one participant withdrew from the study because of facial palsy (maximum grade 2 severity). Perhaps this event of facial palsy was an immune-related AE, as nerve damage resulting in palsy may have been due, in part, to an enhanced immune response to viral infection and was managed with corticosteroids. At least one case report of bilateral facial palsy has been reported in the literature in a participant receiving ipilimumab for melanoma, and the facial palsy was considered likely to be immune-mediated [14]. The most commonly reported AE was grade 1 diarrhea, which did not result in dose limitation and did not require treatment with corticosteroids. Diarrhea is reported as an immune-related AE in clinical trials of ipilimumab conducted in patients with melanoma and other advanced solid tumors [9–12]. Data from a retrospective review of 1498 patients with advanced melanoma from 14 completed ipilimumab clinical trials showed the most commonly observed AEs of any grade were gastrointestinal (diarrhea, colitis, enterocolitis, and perforation), dermatologic (rash and pruritus), hepatic, neurological, and endocrine events [15]. With the exception of diarrhea, which was grade 1 and did not result in dose modifications or corticosteroid treatment in this study, AEs commonly associated with ipilimumab therapy in previous clinical trials in patients with cancer were not noted in this study in participants with HIV-1 infection. Although the present study was not placebo-controlled, the occurrence, frequency, and severity of the AEs in the infections and infestations category are largely consistent with that observed in outpatient HIV practice.

This study also demonstrated that changes in HIV-1 RNA occurred in the majority of participants. Two participants, both of whom received lower doses of ipilimumab, had decreases in HIV-1 RNA, whereas most participants who received higher doses of ipilimumab had increases in HIV-1 RNA. In a setting of uncontrolled viremia, it is possible that an unintended effect of more widespread T-cell activation with higher doses of ipilimumab may be the presentation of increased targets for viral replication. At lower doses of ipilimumab, the HIV-1 RNA decrease seen weeks after ipilimumab administration might be explained if an immune response more specifically targeted to HIV-1 was selectively augmented or elicited. A study of another immune checkpoint inhibitor, nivolumab (anti-programmed death 1), in patients with chronic HCV infection showed reductions in viral load observed in some participants weeks after receiving a single dose of nivolumab [16].

The 2 study participants who met the protocol-defined criteria for a significant decrease in HIV-1 RNA had some common characteristics. Both experienced their HIV-1 RNA decline “late” (day 57 or beyond) in the study and had a grade 1 immune-related AE of diarrhea. In one participant, HIV-1 RNA decline occurred on days 71 and 85 (Fig 3A) and diarrhea was observed on days 2–4. In the other, HIV-1 RNA decline occurred on days 57 and 71 (data not shown) and diarrhea was observed on day 84; this participant was also noted to have a transiently positive ANA on day 29. In both participants, no further HIV-1 RNA measurements were taken after day 85. No other meaningful baseline demographic or disease characteristics were common between these participants. Change in CD4 count from baseline and results of immunologic assays did not show any differences in immunologic outcomes in the participants with HIV-1 RNA decreases compared with those who showed no change or an increase.

Exploring characteristics of the 14 study participants with increases in HIV-1 RNA reveals that 13 participants (92.8%) were in the higher dose groups (ipilimumab 3 and 5 mg/kg). Among participants with an increase in HIV-1 RNA, the level of HIV-1 RNA increase was not different between participants who were or were not on cART, suggesting no meaningful correlation between cART use and HIV-1 RNA increase. Of the 14 participants with HIV-1 RNA increase, 8 had an increase as soon as day 14 after the first dose. Among the 14 participants with an increase in HIV-1 RNA, 2 had grade 1 diarrhea considered unlikely to be related to ipilimumab, 1 had lethargy and dizziness (both grade 1) considered possibly related to ipilimumab, and 1 had grade 2 facial palsy considered possibly related to ipilimumab.

In most participants, CD4 count fluctuated within a range of ± 200 cells/μL and changes in CD4 counts were not appreciably greater than the level of variation observed during the screening period, were transient, and appeared unrelated to dose and HIV-1 RNA level. In summary, the CD4 changes observed during ipilimumab treatment do not appear clinically meaningful. There is a previously published single case study in which significant increases in CD4 T-cell counts were observed after treatment with ipilimumab in an HIV-1-infected patient who also had melanoma [8]. In that case study, the patient was on a stable regimen of cART with fully suppressed virus by standard measures. The participants in our study had uncontrolled viremia.

The clearance of ipilimumab in participants with HIV-1 (0.22 mL/h/kg or 15.7 mL/h using an average weight of 70 kg) was similar to that in participants with melanoma (16.8 mL/h). The steady-state C_min_ of ipilimumab observed in HIV-1-infected participants was lower than that reported in participants with melanoma. This is expected, due to the difference in dosing frequency (every 4 weeks in HIV-1-infected participants vs every 3 weeks in participants with melanoma).

Limitations on the interpretation of this study’s results include the small number of participants, absence of a placebo control arm, imbalance between cohorts with respect to cART status, limited PK data availability, and limited immunologic analyses. Additionally, study participants had a mean duration of disease of 15.6 years, and the chronic viremia may have caused difficult-to-reverse T-cell exhaustion [17]. It is possible that patients with a more recent HIV diagnosis and potentially more active T-cell population might respond differently to treatment with ipilimumab.

Since the time of study completion reported here, immune checkpoint inhibitors have been evaluated in other settings of patients with chronic viral infections, including patients with HIV, with and without concomitant malignancies, and in patients with chronic HCV infection. The results of these preliminary studies of treatment with antibodies directed at CTLA-4 [8], programmed death 1 [18, 19], or programmed death ligand 1 (PD-L1) [20] have not demonstrated uniform effects on viral load or effector cell counts.

The mean percentage of HIV-1-specific IFN-γ^+^ CD8^+^ cells in HIV-infected individuals well controlled on cART was observed to increase slightly over 28 days after treatment with a single dose of an anti-PD-L1 antibody (BMS-936559) [20]. This phenomenon was driven by increases in 2 of 6 participants. Similarly, the HIV-1-specific IFN-γ^+^ CD8^+^ cell count increased in 1 HIV-infected individual who received repeated injections of nivolumab for non-small cell lung cancer over a 120-day period [19]. Ipilimumab treatment of an HIV-infected patient on antiretroviral therapy increased CD4^+^ T cells, predominantly total memory and effector memory cells, post-infusion along with transient increases in CD8^+^ T cells without change in cell activation. Furthermore, ipilimumab increased cell-associated unspliced HIV RNA and a subsequent decline in plasma HIV RNA [8]. Reports in macaques found that treatment with ipilimumab was associated with increases in mean serum SIV levels [21], but with decreases in SIV levels in lymph nodes [22]. The findings of a potential effect on effector cell function are consistent with those of Hryniewicz et al, who also observed a numerical increase in IFN-γ^+^ CD8^+^ cell counts in SIV-infected macaques treated with ipilimumab [22].

In summary, this study showed that ipilimumab administration to a small number of participants with chronic HIV-1 infection in whom virus was not suppressed was tolerated as well, if not better, as in patients with advanced melanoma [23]. Potential concerns for large HIV-1 RNA increases or CD4 count decreases were not observed in the current study. In this population, administration of ipilimumab resulted in decreases in HIV-1 RNA in 2 participants, although the changes were not clearly related to cART status or changes in CD4 counts. Fourteen participants had increases in HIV-1 RNA levels and all but 1 of these participants were in the higher ipilimumab dose cohorts; among these 14 participants, no clinically meaningful correlation with cART use was observed. Based on the results of this small study, there is no safety concern that precludes further study of immune checkpoint inhibitors in patients with HIV-1 infection. Further studies are warranted to expand our understanding of strategies to augment immune response to HIV-1 and to extend the work described here and that of others for ipilimumab [8], nivolumab [18, 19], and PD-L1 [20].

## Supporting information

S1 Appendix CONSORT checklist. (PDF)

S2 Appendix Study protocol. (PDF)
