# Supplementary material for: An open-label, multiple ascending dose study of the anti-CTLA-4 antibody ipilimumab in viremic HIV patients
Source: PLoS One. 2018 Jun 7;13(6):e0198158. doi: 10.1371/journal.pone.0198158 (PMC5991705; doi:10.1371/journal.pone.0198158)
Supplement: S2 Appendix — (PDF) [file pone.0198158.s002.pdf]

Bristol-Myers Squibb Company

Clinical Development

Product No. MDX-010

Protocol No. MDX010-10

A Phase I Open-Label, Dose-Escalation Study of MDX-010 Administered Monthly as Immunotherapy in Patients Infected with Human Immunodeficiency Virus

Document type:	Summary of Changes to Protocol: Amendment 1
Development Phase:	Phase I
Document status:	Final
Release date:	31 January 2003
Number of pages:	6

Property of Medarex, Inc.

Confidential
The information in this document contains trade secrets and commercial information that are privileged or confidential and may not be used, divulged, published, or otherwise disclosed without the written consent of Medarex, Inc. These restrictions on disclosure will apply equally to *all* future information supplied to you which is indicated as *privileged* or *confidential*.

The following changes (indicated in shaded area) have been made as Amendment 1 to the Protocol for Study MDX010-10:

Page 7, Time and Events Schedule, Clinic Visits and Routine Laboratory Measurements

Change from:

Screening and monitoring serologies	•[7]	•[8]							•[9]					•[9]		•[9]

To:

Screening and monitoring serologies	•[7]								•[8]					•[8]		•[8]

Reason for change: Positive ANA and rheumatoid factor results will lead to reflex testing of additional serologic parameters by the laboratory.

Note: All subsequent footnotes were renumbered.

Add:

Note: All patients will have Visit 16 (Termination Visit) evaluations performed at the time of completion or early withdrawal.

Reason for change: Clarification of Visit 16.

Change Footnotes 7 and 8 to read:

7. Initial screening serologies to include CRP, ANA, VDRL, sIL-2r, TNFα, sTNFr, neopterin, rheumatoid factor, hepatitis B Ag and Ab, and hepatitis C Ab. If ANA or rheumatoid factor are positive, then anti-cardiolipin antibody, anti-thyroid antibody, and TSH will be reflexively measured. If hepatitis B or C are positive, the relevant quantitative titer will be reflexively measured.
8. All patients will have ANA and rheumatoid factor tested at Visits 9, 14, and 16 (termination). Patients with any positive serology will have the full complement of serologies monitored at the remaining Visits (9, 14, and 16).

Reason for change: Additional monitoring for ANA and rheumatoid factor as requested by CBER and clarification of additional testing procedures.

Replace Footnote 10:

PK sampling Intensive: -30 minutes and 120 minutes, and 2 hours post-infusion start time.

With Footnote 9:

PK sampling Intensive: -30 minutes and 120 minutes and 4 hours post-infusion start time.

Reason for change: Clarification of pharmacokinetic sampling time.

Page 22, Section 6.1, **MDX-010 Dosage**

Change paragraph to read:

Beginning Day 1 (Visit 3), MDX-010 will be administered as a 90 minute i.v. infusion, using a volumetric pump, at an initial dosage of 0.1 mg/kg/dose; it is **not** to be administered as an i.v. push or bolus injection. Infusions of MDX-010 will be administered every 28 days for 2 doses. Succeeding dosage levels will include 1.0 and 3.0 mg/kg/dose. Cohorts of 3 to 6 patients will be treated in each escalating dosage level until DLT or MTD is achieved.

Reason for change: Clarification of Visit day.

Page 28, Section 9.1.2.2, **Pre-Entry (Visit 2)**

Change from:

- Screening serology:
  If ANA or rheumatoid factor were positive at prestudy Visit 1, the following screening serologies will be performed at this visit:
  - Anti-cardiolipin Ab;
  - TSH; and
  - Anti-thyroid antibody

  If hepatitis B or C were positive at prestudy Visit 1, quantitative viral load will be determined at this visit.

To:

- Screening serology:
  If ANA or rheumatoid factor were positive at prestudy Visit 1, the following screening serologies will have been reflexively tested and should be noted at this visit:
  - Anti-cardiolipin Ab;

- TSH; and
- Anti-thyroid antibody

If hepatitis B or C were positive at prestudy Visit 1, quantitative viral load will have been reflexively tested and should be noted at this visit.

Reason for change: Consistent with previous changes

Page 29, Section 9.1.3, Treatment Phase - Infusion

Change from:

- Serologic monitoring:
  - CRP, sIL-2r, TNFα, sTNFr, and neopterin will be monitored at Visits 9 and 14
  - If ANA, rheumatoid factor, anti-cardiolipin Ab, TSH, or anti-thyroid Ab were positive during the screening phase, these parameters will be monitored at Visits 9 and 14
  - If hepatitis B or C viral loads were detectable during the screening phase, these parameters will be monitored at Visits 9 and 14

To:

- Serologic monitoring:
  - CRP, sIL-2r, TNFα, sTNFr, and neopterin will be monitored at Visits 9 and 14
  - ANA and rheumatoid factor will be tested in all patients at Visits 9 and 14
  - If ANA, rheumatoid factor, anti-cardiolipin Ab, TSH, or anti-thyroid Ab were positive during the screening phase or become positive, then all of these parameters will be monitored at Visits 9 and 14
  - If hepatitis B or C viral loads were detectable during the screening phase, these parameters will be monitored at Visits 9 and 14

Reason for change: Additional monitoring for ANA and rheumatoid factor as requested by CBER and clarification of serology testing during treatment.

Page 30, Section 9.1.4, Follow-Up Phase

Change from:

- Serologic monitoring:
  - CRP, sIL-2r, TNFα, sTNFr, and neopterin will be monitored at Visits 9 and 14
  - If ANA, rheumatoid factor, anti-cardiolipin Ab, TSH, or anti-thyroid Ab were positive during the screening phase, these parameters will be monitored at Visits 9 and 14
  - If hepatitis B or C viral loads were detectable during the screening phase, these parameters will be monitored at Visits 9 and 14

To:

- Serologic monitoring:
  - CRP, sIL-2r, TNFα, sTNFr, and neopterin will be monitored at Visit 16 (Termination)
  - ANA and rheumatoid factor will be tested in all patients at Visit 16 (Termination)
  - If ANA, rheumatoid factor, anti-cardiolipin Ab, TSH, or anti-thyroid Ab were positive during the screening phase or become positive, then all of these parameters will be monitored at Visit 16 (Termination).
  - If hepatitis B or C viral loads were detectable during the screening phase, these parameters will be monitored at Visit 16 (Termination).

Reason for change: Correction of incorrect Visit Number, additional monitoring for ANA and rheumatoid factor as requested by CBER, and clarification of serology testing during treatment.

Page 30, Section 9.1.5, **Termination Visit**

Change title to read:

Section 9.1.5, Termination Visit (Visit 16)

Reason for change: Clarification of Termination Visit as Visit 16.

Page 30, Section 9.1.5, **Termination Visit**

Change paragraph to read:

A Study Completion/Early Withdrawal visit (Visit 16) will be scheduled for all patients enrolled in the study. If patients prematurely withdraw, these procedures will be completed on the day of withdrawal from the study.

Reason for change: Clarification of Termination Visit as Visit 16.

Page 31, Section 9.2, **Treatment Phase:**

Clarify:

Visits 3 and 9: 30 minutes prior to infusion, 120 minutes and 4 hours post infusion start time 0.

Reason for change: Clarification of pharmacokinetic sampling time.

Pages 39 and 40, Section 12.4.6, Pharmacokinetic Parameters, Definitions and Calculation of PK Parameters

Add:

$t_{1/2}$ Elimination half-life; determined as $0.693/\lambda_z$.

Reason for change: Addition of $t_{1/2}$ as requested by CBER.

Clinical Development

Product No. MDX-010

Protocol No. MDX010-10

A Phase I Open-Label, Dose-Escalation Study of MDX-010 Administered Monthly as Immunotherapy in Subjects Infected with Human Immunodeficiency Virus

Document type:	Summary of Changes to Protocol: Amendment 2
Development Phase:	Phase I
Document status:	Final
Release date:	27 June 2003
Number of pages:	3

Property of Medarex, Inc.

Confidential
The information in this document contains trade secrets and commercial information that are privileged or confidential and may not be used, divulged, published, or otherwise disclosed without the written consent of Medarex, Inc. These restrictions on disclosure will apply equally to *all* future information supplied to you which is indicated as *privileged* or *confidential*.

The following changes (indicated in the shaded areas) have been made as Amendment 2 to the Protocol for Study MDX010-10:

Please note the following global change: throughout the protocol, patients will be referred to as subjects.

Page 7, Time and Events Schedule, Clinic Visits and Routine Laboratory Measurements

Change from:

Table 1: **Time and Events Schedule**

Examination	Screening		Treatment (+/- 1 day)												Follow-Up (+/-3 days)	
Time point (Days)	28 to 21	-7	1	4	8	15	22	25	29	32	36	43	50	57	71	85
Visit	1	2	3	4	5	6	7	8	9	10	11	12	13	14	15	16
**Study Interventions**																
Tetanus booster								•								

To:

Examination	Screening		Treatment (+/- 1 day)												Follow-Up (+/-3 days)	
Time point (Days)	28 to 21	-7	1	4	8	15	22	25	29	32	36	43	50	57	71	85
Visit	1	2	3	4	5	6	7	8	9	10	11	12	13	14	15	16
**Study Interventions**																
Tetanus booster									•							

Summary of Change:

A tetanus booster will be required at Visit 9 and no longer at Visit 8.

Reason for Change:

In a series of preclinical studies conducted to examine the effect of timing on the administration of CTLA-4 antibodies and the extent of immune response to a tumor antigen vaccine in a murine model, it was noted that when the timing of the antibody injection was delayed 3 days beyond the vaccination, there was a marked decrease in efficacy of the immune response to the vaccine. The optimal response was obtained when the vaccine and antibody were both administered on the same day. Based upon these results, the protocol will be amended to allow for the tetanus booster to be administered at the same visit as the antibody infusion.

Page 19, Overview of Study Design

Change from:

All patients will receive a tetanus booster at Week 4 of the study, 3 days prior to the second infusion of MDX-010.

To:

All subjects will receive a tetanus booster at Visit 9 (Day 29) of the study, to be given at the end of the observation period following the second infusion of MDX-010.

Summary of Change:

Clarification of the timing of the tetanus booster.

Reason for Change:

Consistent with previous change.

Page 29, Section 9.1.3., Treatment Phase—Infusion

Change from:

- Tetanus booster (Visit 8 only, 3 to 4 days prior to second infusion of MDX-010)

To:

- Tetanus booster (Visit 9 only; to be given at the end of the observation period following the second infusion of MDX-010)

Summary of Change:

Clarification of the timing of the tetanus booster.

Reason for Change:

Consistent with previous change.

Clinical Development

Product No. MDX-010

Protocol No. MDX010-10

A Phase I Open-Label, Dose-Escalation Study of MDX-010 Administered Monthly as Immunotherapy in Subjects Infected with Human Immunodeficiency Virus

Document type:	Summary of Changes to Protocol: Amendment 3
Development Phase:	Phase I
Document status:	Final
Release date:	27 February 2004
Number of pages:	23

Property of Medarex, Inc.

Confidential
The information in this document contains trade secrets and commercial information that are privileged or confidential and may not be used, divulged, published, or otherwise disclosed without the written consent of Medarex, Inc. These restrictions on disclosure will apply equally to *all* future information supplied to you which is indicated as *privileged* or *confidential*.

The following changes (indicated in the shaded areas) have been made as Amendment 3 to the Protocol for Study MDX010-10:

Page 5, Synopsis, Objectives

Change first paragraph to read:

The primary objective of the study is to establish the safety and tolerability of 2 or 4 doses of MDX-010 in human immunodeficiency virus (HIV)-infected subjects.

Page 5, Synopsis, Overview of Study Design

Change paragraphs to read:

This is a Phase I, multicenter, open-label, dose-escalation study to be conducted in HIV-infected subjects. Up to 50 subjects are expected to be enrolled, with 3 to 6 subjects at each of 5 dose levels or dosing regimens. One or 2 cohorts that exhibit safety and evidence of efficacy will be expanded to 16 patients.

Subjects will receive 2 to 4 doses, administered 4 weeks apart, as detailed in Dosage and Administration. Dosing of subjects at the next higher dose level in each group will not be initiated until 10 days after all subjects in the previous cohort have received both doses. For the first 3 dose levels, cohorts will accrue at least 3 subjects, with 6 subjects in the maximum tolerated dose (MTD) cohort. The MTD dose will be the highest dose where no more than 1 of 6 has experienced a dose-limiting toxicity (DLT). If 1 of 3 subjects experience a DLT, the cohort will be increased to 6 subjects. If 2 or more of the 6 subjects experience DLT, that dose will have exceeded the MTD and a lower dose will accrue to a total of 6 subjects. If this lower dose level is well tolerated and none of the 6 subjects experience a DLT, an additional intermediate dose level may be defined by protocol amendment. If the MTD is not established in the first 6 patients administered 2 doses of MDX-010 at 3.0 mg/kg, additional subjects (6/group) will be randomized to receive either 4 doses of MDX-010 at 3.0 mg/kg or 2 doses of MDX-010 at 5.0 mg/kg.

Reason for Changes:

MDX-010 has been extremely well tolerated in the first 2 completed cohorts of this study at doses of 0.1 and 1.0 mg/kg. Accumulating experience in multiple other protocols in oncology has routinely dosed MDX-010 at 3 mg/kg q3-4 weeks for 4 doses, and higher doses of 5 mg/kg and above are currently under exploration. Therefore, it is reasonable to explore these more typical doses in the HIV infected subjects as well, assuming that the third cohort of 3 mg/kg q4 weeks x 2 doses is completed without exceeding the MTD. The new cohorts exploring the higher doses of 3 mg/kg q4weeks x 4 doses and 5 mg/kg q4 weeks x 2 doses will be studied simultaneously with open label central randomization. One or both of these cohorts that enroll 6 patients without exceeding MTD and show preliminary evidence of activity by decreased viral load and/or increased CD4 count will be expanded to 16 patients in order to get a preliminary sense of clinical efficacy to guide subsequent Phase II studies.

Page 5, Synopsis, Dosage and Administration

Change paragraph to read:

Cohorts of subjects are to be administered MDX-010 at escalating dosage levels of 0.1, 1.0, 3.0, and 5.0 mg/kg/dose, administered as an intravenous (i.v.) infusion. MDX-010 will be administered every 28 days for 2 to 4 doses. If the MTD is not established in the first 6 patients administered 2 doses of MDX-010 at 3.0 mg/kg, additional subjects (6/group) will be randomized to receive either 4 doses of MDX-010 at 3.0 mg/kg or 2 doses of MDX-010 at 5.0 mg/kg. One or 2 cohorts that exhibit safety and evidence of efficacy will be expanded to 16 patients.

Reason for Change:

Consistent with previous changes.

Page 6, Synopsis, Statistical Methods

Change paragraph to read:

The sample size of up to 50 subjects is based on the trial design for dose escalation and safety evaluation requirements, with up to 30 subjects (3 to 6 subjects/cohort) at 5 dose levels or treatment regimens and additional 20 subjects in the selected 2 cohorts for the evaluation of clinical activity. The primary clinical activity parameter is viral load assessment. The other activity parameters include, secondary clinical activity parameter CD4 cell count, immunologic activity parameters CD4 and CD8 cell counts with cytokine response, change in LPA to HIV, Candida, and tetanus antigens, and changes in quantitative anti-tetanus antibody titer. All activity parameters will be summarized using descriptive statistics. A one-sample exact Binomial test will be used for the primary clinical activity parameter in the selected 2 cohorts. The safety parameters include vital signs, clinical laboratory tests, physical examinations, adverse events, diagnostic tests, and special immune function measurements. All safety parameters will be summarized using descriptive statistics. The pharmacokinetic parameters include AUC, C_{max}, and t_{max}. All pharmacokinetic parameters will be summarized using descriptive statistics.

Reason for Change:

Updated statistical methods for consistency with previous changes.

Page 7, Table 1: Time and Events Schedule

Change table as indicated on the following page.

Reason for Change:

Predose blood sampling prior to additional dose of MDX-010 added to enable monitoring of laboratory values and to ensure patient safety.

Note: All subsequent footnotes were renumbered.

Table 1: Time and Events Schedule for Administration of Two Doses

Examination	Screening		Treatment (+/- 1 day)												Follow-Up (+/-3 days)	
**Time point (Days)**	**28 to 21**	**-7**	**1**	**4**	**8**	**15**	**22**	**25**	**29**	**32**	**36**	**43**	**50**	**57**	**71**	**85**
**Visit**	**1**	**2**	**3**	**4**	**5**	**6**	**7**	**8**	**9**	**10**	**11**	**12**	**13**	**14**	**15**	**16**
Informed consent	•															
Inclusion/exclusion criteria[1]	•															
Demographics and medical history[2]	•															
**Clinic Visits and Routine Laboratory Measurements**																
Physical examination	•[3]		•[4]						•[4]							•[3]
Vital sign measurements	•	•	•[5]	•	•	•	•	•	•[5]	•	•	•	•	•	•	•
Electrocardiograph	•															
Chest radiograph	•															
Pregnancy test		•[6]	•[6]						•[6]							
Screening and monitoring serologies	•[7]								•[8]					•[8]		•[8]
Clinical chemistries	•	•[9]	•			•		•[9]	•					•		•
Hematology	•	•[9]	•	•	•	•	•	•[9]	•	•	•	•	•	•	•	•
Urinalysis[10]	•		•						•					•		•
**Study Interventions**																
MDX 010 infusion			•						•							
Tetanus booster									•							
**Standard HIV Laboratory Measurements**																
Plasma HIV RNA	•	•	•	•	•	•	•	•	•	•	•	•	•	•	•	•
CD4/CD8 counts	•	•	•	•	•	•	•	•	•	•	•	•	•	•	•	•
**MDX-010 Pharmacokinetic and HAHA Measurements**																
MDX 010 PK sampling			•[11]	•[12]	•[12]	•[12]	•[12]		•[11]	•[12]	•[12]	•[12]		•[12]		•[12]
Plasma sample for HAHA			•[13]						•[13]							•
**Special Immune Function Measurements**																
Quantitative tetanus EIA	•		•					•	•	•	•	•	•	•		•
T cell subsets flow panel		•	•	•		•		•	•		•		•	•		•
LPA panel		•	•	•		•		•	•		•		•	•		•
Cytokine production (EIA)		•						•						•		
Stored cryopreserved PBMC		•						•						•		
ICC IHC (cryopreserved PBMC)		•						•						•		
**Concomitant medications**	•	•	•	•	•	•	•	•	•	•	•	•	•	•	•	•
**Adverse events**	•[14]	•	•	•	•	•	•	•	•	•	•	•	•	•	•	•

Note: All subjects will have Visit 16 (Termination Visit) evaluations performed at the time of completion or early withdrawal.
Footnotes and Abbreviations:
EIA enzyme immunoassay; PBMC peripheral blood mononuclear cell; HAHA human anti human antibodies;
ICC immunocytochemistry; HC immunohistochemistry

1. To include pretherapy viral load history and pretherapy CD4 count history if known, but not required.
2. Medical history including prior antiretroviral therapy and concurrent medical conditions.
3. Complete physical examination.
4. Target physical examination to include evaluation of heart, lungs, and abdomen.
5. Vital sign measurements will be obtained every 15 mins during the infusion period and every 30 mins for 2 hours following infusion.
6. Serum β HCG pregnancy test within 7 days prior to the first infusion, and urine pregnancy test taken prior to each infusion.
7. Initial screening serologies to include CRP, ANA, VDRL, sIL 2r, TNFα, sTNFr, neopterin, rheumatoid factor, hepatitis B Ag and Ab, and hepatitis C Ab. If ANA or rheumatoid factor are positive, then anti cardiolipin antibody, anti thyroid antibody, and TSH will be reflexively measured. If hepatitis B or C are positive, the relevant quantitative titer will be reflexively measured.
8. All subjects will have ANA and rheumatoid factor tested at Visits 9, 14, and 16 (termination). Subjects with any positive serology will have the full complement of serologies monitored at the remaining Visits (9, 14, and 16).
9. Central laboratory results must be reviewed and the results must be found to be acceptable by the Investigator before additional MDX 010 can be administered.
10. Urinalysis: analysis including microscopic/gross exam and laboratory measurement.
11. PK sampling Intensive: 30 minutes and 120 minutes and 4 hours post infusion start time.
12. PK sampling one sample to be drawn.
13. Sample obtained prior to infusion with MDX 010.
14. During the screening period, baseline signs and symptoms will be collected following signing of the Informed Consent.

Pages 8 and 9, Add

Table 2 (found on the following 2 pages)

Reason for Change:

Time and events schedule outlining procedures for patients receiving 4 doses.

Table 2: Time and Events Schedule for Administration of Four Doses

Examination	Screening		Treatment (+/- 1 day)																					Follow-Up (+/-3 days)	
**Time point (Days)**	**–28 to –21**	**-7**	**1**	**4**	**8**	**15**	**22**	**25**	**29**	**32**	**36**	**43**	**50**	**53**	**57**	**64**	**71**	**78**	**85**	**92**	**99**	**106**	**113**	**127**	**141**
**Visit**	**1**	**2**	**3**	**4**	**5**	**6**	**7**	**8**	**9**	**10**	**11**	**12**	**13**	**14**	**15**	**16**	**17**	**18**	**19**	**20**	**21**	**22**	**23**	**24**	**25**
Informed consent	•																								
Inclusion/exclusion criteria[1]	•																								
Demographics and medical history[2]	•																								
**Clinic Visits and Routine Laboratory Measurements**																									
Physical examination	•[3]		•[4]						•[4]						•[4]				•[4]						•[3]
Vital sign measurements	•	•	•[5]	•	•	•	•	•	•[5]	•	•	•	•	•	•[5]	•	•	•	•[5]	•	•	•	•	•	•
Electrocardiograph	•																								
Chest radiograph	•																								
Pregnancy test		•[6]	•[6]						•[6]						•[6]				•[6]						
Screening and monitoring serologies	•[7]								•[8]						•[8]				•[8]						•[8]
Clinical chemistries	•	•[9]	•			•		•[9]	•					•[9]	•			•[9]	•				•		•
Hematology	•	•[9]	•	•	•	•	•	•[9]	•	•	•	•	•	•[9]	•[9]	•	•	•[9]	•	•	•	•	•	•	•
Urinalysis[10]	•		•						•					•											•
**Study Interventions**																									
MDX-010 infusion			•						•						•				•						
Tetanus booster									•																
**Standard HIV Laboratory Measurements**																									
Plasma HIV RNA	•	•	•	•	•	•	•	•	•	•	•	•	•	•	•	•	•	•	•	•	•	•	•	•	•
CD4/CD8 counts	•	•	•	•	•	•	•	•	•	•	•	•	•	•	•	•	•	•	•	•	•	•	•	•	•
**MDX-010 Pharmacokinetic and HAHA Measurements**																									
MDX-010 PK sampling			•[11]	•[12]	•[12]	•[12]	•[12]		•[11]	•[12]	•[12]	•[12]		•[12]	•[11]	•[12]	•[12]	•[12]	•[11]	•[12]	•[12]	•[12]	•[12]		•[12]
Plasma sample for HAHA			•[13]						•[13]						•[13]				•[13]						•
**Special Immune Function Measurements**																									
Quantitative tetanus EIA	•		•					•	•	•	•	•	•	•		•		•		•			•		•
T cell subsets – flow panel		•	•	•		•		•	•		•		•	•		•		•		•			•		•
LPA panel		•	•	•		•		•	•		•		•	•		•		•		•			•		•
Cytokine production (EIA)		•						•								•				•			•		
Stored cryopreserved PBMC		•						•								•				•			•		
ICC-IHC (cryopreserved PBMC)		•						•								•				•			•		
**Concomitant medications**	•	•	•	•	•	•	•	•	•	•	•	•	•	•	•	•	•	•	•	•	•	•	•	•	•
**Adverse events**	•[14]	•	•	•	•	•	•	•	•	•	•	•	•	•	•	•	•	•	•	•	•	•	•	•	•
Footnotes may be found on the following page.																									

Table 2: Time and Events Schedule for Additional Dosing (continued)

Footnotes:

Note: All subjects will have Visit 28 (Termination Visit) evaluations performed at the time of completion or early withdrawal.

Footnotes and Abbreviations:

EIA = enzyme immunoassay; PBMC = peripheral blood mononuclear cell; HAHA = human anti-human antibodies; ICC = immunocytochemistry; HC = immunohistochemistry

1. To include pretherapy viral load history and pretherapy CD4 count history if known, but not required.
2. Medical history including prior antiretroviral therapy and concurrent medical conditions.
3. Complete physical examination.
4. Target physical examination to include evaluation of heart, lungs, and abdomen.
5. Vital sign measurements will be obtained every 15 mins during the infusion period and every 30 mins for 2 hours following infusion.
6. Serum β-HCG pregnancy test within 7 days prior to the first infusion, and urine pregnancy test taken prior to each infusion.
7. Initial screening serologies to include CRP, ANA, VDRL, sIL-2r, TNFα, sTNFr, neopterin, rheumatoid factor, hepatitis B Ag and Ab, and hepatitis C Ab. If ANA or rheumatoid factor are positive, then anti-cardiolipin antibody, anti-thyroid antibody, and TSH will be reflexively measured. If hepatitis B or C are positive, the relevant quantitative titer will be reflexively measured.
8. All subjects will have ANA and rheumatoid factor tested at Visits 9, 15, 19, and 25 (termination). Subjects with any positive serology will have the full complement of serologies monitored at the remaining Visits (9, 15, 19, and 25).
9. Central laboratory results must be reviewed and the results must be found to be acceptable by the Investigator before additional MDX-010 can be administered.
10. Urinalysis: analysis including microscopic/gross exam and laboratory measurement.
11. PK sampling – Intensive: -30 minutes and 120 minutes and 4 hours post-infusion start time.
12. PK sampling – one sample to be drawn.
13. Sample obtained prior to infusion with MDX-010.
14. During the screening period, baseline signs and symptoms will be collected following signing of the Informed Consent.

Page 8, (currently Page 10) Signature Page for Medarex, Inc.

Add:

Approved by:

[redacted]

[redacted]	Signature	Date

Reason for Change:

Addition of new personnel.

Page 11 (currently Page 13) Abbreviations

Change from

NRTIs	nucleoside reverse transcriptase inhibitors

To

(N/Nt)RTIs	nucleoside/nucleotide reverse transcriptase inhibitors

Reason for Change:

To include both nucleoside and nucleotide inhibitors.

Note: This abbreviation was updated throughout the document.

Page 17 (currently Page 19) Section 1.4, Rationale for Exploring MDX-010 Therapy in HIV-Infected Subjects

Remove paragraph that reads:

MDX-010 is a human antibody to CD152 that blocks the interaction of CD152 with CD80/86. Binding by MDX-010 results in disinhibition of cellular immune responses that would otherwise be downmodulated after the induction of CD152 and its competition with CD28 for binding to the CD80/86 receptors on antigen-presenting cells (APCs). Experimental and clinical data indicate that increased expression of CD152 is present in the T cells of HIV-infected subjects, and that this excess expression may interfere with the elaboration of an effective immune response that can control HIV replication and disease progression.[7,8] Signalling via CD152 may increase susceptibility of primary

lymphocytes to macrophage tropic (R5) strains of HIV.[10] Blockade of CD152 by MDX-010 may therefore augment an effective immune response to HIV in infected subjects. Two Phase I trials have been completed with MDX-010 in subjects with prostate and melanoma cancers. MDX-010 was well tolerated at single doses of 3 mg/kg, displayed a half life of 12 to 15 days, with preliminary indications of efficacy.[23,24] It is currently undergoing Phase II evaluations in a variety of cancers, with both multiple dosing and in combination with vaccines or chemotherapy to assess its utility to enhance immune responses to tumors and induce their regression.

Reason for Change:

Removal of repetitive paragraph.

Page 18 (currently page 19) Section 1.4, Rationale for Exploring MDX-010 Therapy in HIV-Infected Subjects

Change paragraph to read:

Subjects whose antiretroviral therapy is no longer suppressing viremia are to be enrolled in this study. These will be HAART-experienced subjects with at least 2 episodes of virologic failure, defined as documented virus breakthrough while on therapy on at least 2 occasions, and will have virus with documented resistance to drugs in each therapeutic class (RTIs, NNRTIs, and PIs) and also to the newer fusion inhibitor if available. Despite breakthrough viremia, subjects may still benefit from their regimen and preserve their CD4 counts, either because the level of viremia on-therapy is lower than that off-therapy, or the breakthrough virus may not be as "fit" and pathogenic as "wild-type" virus.[26] Subjects who are no longer taking previously failed HAART due to intolerance or to regimen fatigue will also be permitted. Ultimately these subjects will require additional therapies to prevent progression of disease, and are in need of new treatment modalities. Therefore these subjects are appropriate for initial studies with MDX-010.

Reason for Change:

To broaden the criteria for failure of HAART therapy.

Page 19 (currently Page 21) Section 3, Overview of Study Design

Change paragraphs to read:

This is a Phase I, open-label, multicenter, dose-escalation study in HIV-infected subjects. The study is designed to determine the safety and tolerability profile of 2 or 4 doses of MDX-010, a human mAb. It is also intended to characterize the pharmacokinetic profile of MDX-010 administered i.v. once every 4 weeks for 2 or 4 doses, to assess any preliminary clinical activity to augment an effective immune response against HIV, and to assess for induced cellular and humoral immune responses toward HIV, Candida, and a tetanus booster.

During the first visit, after obtaining informed consent, screening evaluations will begin. Subject eligibility will be determined by the inclusion and exclusion criteria on prestudy evaluation listed in Section 4.

During the treatment phase, subjects will be required to visit the Investigator's office or clinic every 3 to 7 days for 8 weeks (subjects receiving 2 doses) or 16 weeks (subjects receiving 4 doses) for administration of study drug, pharmacokinetic sampling, vital sign measurements, physical examinations, clinical laboratory testing, viral load and immunologic assessments, and/or the collection of adverse events.

Reason for Change:

Consistent with previous changes.

Page 20 (currently Page 22) Section 4, Study Population

Change paragraph to read:

Subjects with active HIV infection, who are on antiretroviral therapy and who have detectable viremia (viral load by RT-PCR [VL] between 1,000 and 100,000 copies/mL), and CD4 > 100 cells/mm^3 will be enrolled into the study. A maximum of 50 subjects (3 to 6 subjects per group, with 16 subjects in 1 or 2 cohorts that demonstrates safety and evidence of efficacy) will be enrolled. The specific inclusion and exclusion criteria for enrolling subjects in this study are described in the following sections.

Reason for Change:

Consistent with previous changes.

Page 20, (currently Page 22) Section 4.1, Inclusion Criteria:

Change criterion to read:

5. Current ART regimen follows at least 2 previous changes for documented virologic failure (breakthrough viremia while adherent to a regimen containing ≥ 3 antiretroviral agents, and exclusive of prior mono or dual therapy), and documented resistance tests demonstrate the presence of at least 1 mutation to each major therapeutic class of ART [(N/Nt)RTI, NNRTI, PI]. Subjects failing fusion inhibitors do not need resistance tests. If a therapeutic class of agents is not being used for reasons of intolerance, then documentation of the signs of intolerance can substitute for resistance mutations to that class. Therefore, subjects who are not currently taking antiretroviral therapy for reasons of resistance, intolerance, or regimen fatigue may also be enrolled.

Reason for Change:

The objective of the inclusion criteria is to identify subjects for whom current accepted HAART is no longer a viable option, and therefore an appropriate population for this first study of MDX-010 in HIV infected subjects. Failure of previous regimens must be documented as either due to persistent viremia in the face of ongoing HAART due to the accrual of resistance mutations to all classes of HIV inhibitors, or the inability to take inhibitors due to intolerance or fatigue (unwillingness to continue HAART after prior courses had failed).

Page 21 (currently Page 24) Section 4.2., Exclusion Criteria

Change criterion to read:

13. Pregnant or breastfeeding women; women of childbearing potential must be using an acceptable barrier method of birth control and prevention of venereal disease and must agree to continue using this method during the course of the study.

Reason for Change:

Clarification of wording.

Page 22 (currently Page 24) Section 5.1, Procedures

Add the following paragraph:

To assign the subjects to the 2 cohorts consisting of 16 patients for evaluation of clinical activity, the Biostatistics group at Medarex, Inc. will provide a centralized randomization list to Clinical Operations using SAS procedure PROC PLAN. To keep the balance of the treatment allocation, the randomization scheme will be generated using block size of 4

and will not be stratified by site. The randomization list includes block number, patient number, and treatment group. The Biostatistics group will provide an overall randomization list (both electronic copy and hard copy) to Clinical Operations.

Reason for Change:

Additional patients and additional dosage group required the addition of randomization procedures.

Page 22 (currently Pages 24 and 25) Section 6.1, MDX-010 Dosage

Change paragraph to read:

Beginning Day 1 (Visit 3), MDX-010 will be administered as a 90 minute i.v. infusion, using a 1.2 µm in-line filter and a volumetric pump, at an initial dosage of 0.1 mg/kg/dose; it is **not** to be administered as an i.v. push or bolus injection. Infusions of MDX-010 will be administered every 28 days for 2 or 4 doses. Succeeding dosage levels will include 1.0, 3.0, and 5.0 mg/kg/dose. If the MTD is not established in the first 6 subjects administered 2 doses of MDX-010 at 3.0 mg/kg, additional subjects (6/group) will be randomized to receive either 4 doses of MDX-010 at 3.0 mg/kg or 2 doses of MDX-010 at 5.0 mg/kg. One or 2 cohorts that exhibit safety and evidence of efficacy will be expanded to 16 subjects.

Reason for Change:

Addition of in-line filter and consistency with previous changes.

Page 23 (currently Pages 25 and 26) Section 6.2, Dose Escalation

Change paragraph to read:

Dose escalation will start at 0.1 mg/kg; succeeding dose levels will include 1.0, 3.0, and 5.0 mg/kg. Cohorts of 3 to 6 subjects will be treated at each of the 5 dose levels or treatment regimens. Subjects will receive MDX-010 at 4-week intervals for 2 or 4 doses.

For the first 3 cohorts, in the absence of dose-limiting toxicity (DLT) in the first 3 subjects at each dosage level, enrollment in the next dosage level will begin 10 days after the third subject has tolerated the second dose. Cohorts will accrue at least 3 subjects, with 6 subjects in the MTD cohort. The MTD dose will be the highest dose where no more than 1 of 6 subjects has experienced DLT. If one subject experiences a DLT, the cohort will be increased to 6 subjects. If 2 or more of the 6 subjects experience DLT, that dose will have exceeded the MTD and a lower dose level will accrue to a total

of 6 subjects. If the previous dose level was well tolerated and no subjects experienced DLT at that level, an intermediate dose level may be defined by protocol amendment.

Add the following:

If the MTD is not established in the first 6 subjects administered 2 doses of MDX-010 at 3.0 mg/kg, additional subjects (6/group) will be randomized to receive either 4 doses of MDX-010 at 3.0 mg/kg or 2 doses of MDX-010 at 5.0 mg/kg. One or 2 of these cohorts that exhibit safety and evidence of efficacy will be expanded to 16 subjects.

In the 5 mg/kg cohort, should 2 DLTs occur, this will exceed the MTD and no additional subjects will be enrolled at this dose, but this will not automatically affect the enrollment in the 3 mg/kg cohort. If the 5 mg/kg cohort is expanded to 16 subjects, an occurrence of 6 or more DLTs in the 16 subjects will exceed the MTD.

In the 3 mg/kg cohort, DLTs that occur prior to the planned third dose will be considered together with the prior experience of the cohort that received 2 doses of 3 mg/kg, for a total of 12 subjects. If the total number of DLTs before 3 doses remains at 3 or less out of 12 subjects, dosing will continue, and subjects that were discontinued before the third dose will be replaced for this cohort. If 4 or more DLTs prior to the third dose occur in the group of 12, dosing of all subjects will be stopped, including the 5 mg/kg cohort, since the MTD for 2 monthly doses will have been exceeded.

DLTs that occur after the third dose of MDX-010 in the 3 mg/kg x 4 cohort will be considered separately, and 2 or more DLTs that occur after the third or fourth dose in the first 6 subjects to reach those doses will exceed the MTD for 4 doses of 3 mg/kg given monthly. The determination of exceeding the MTD with the third and fourth doses will not automatically affect dosing in the 5 mg/kg cohort If the 3 mg/kg x 4 doses cohort is expanded to 16 subjects, a total of 6 or more DLTs will exceed the MTD.. In all cases, the DLTs will be reviewed by the sponsor and the investigator to assess whether to maintain, decrease, or stop dosing of other subjects under treatment.

Reason for Change:

Dose escalation rules required for additional dosing groups.

Page 23 (currently Page 26) Section 6.3, Dose-Limiting Toxicity

Change paragraph to read:

A dose-limiting toxicity is defined as an adverse event or a new laboratory abnormality that occurs during or after the 4-week MDX-010 infusion period (up to 1 month post-treatment), judged to be possibly, likely, or definitely related to MDX-010 and meeting any of the following criteria:

Reason for Change:

Clarification of adverse events considered "related."

Page 28 (currently Page 31) Section 9.1.2.2, Pre-entry (Visit 2)

Add the following:

- Clinical Chemistry (as outlined in Section 9.1.2.1.)
- Hematology (as outlined in Section 9.1.2.1.)

Reason for Change:

Clinical chemistry addition for consistency with previous changes. Hematology added for consistency with Time and Events Table.

Page 29 (currently Page 32) Section 9.1.3, Treatment Phase – Infusion

Change title to read:

Treatment Phase for Subjects Receiving Two Doses – Infusion

Reason for Change:

Addition of dosing regimen required Treatment Phases to be defined for patients receiving 2 doses and patients receiving 4 doses.

Page 29 (currently Page 32) Section 9.1.3, Treatment Phase for Subjects Receiving Two Doses – Infusion

Change parameters to read:

- Hematology* (as outlined in Section 9.1.2.1)
- Clinical Chemistry* (Visits 3, 6, 9, and 14; as outlined in Section 9.1.2.1)
- Urinalysis (Visits 3, 9, and 14; as outlined in Section 9.1.2.1)

* Local CBC and chemistry panel: results to be reviewed and found acceptable prior to the second infusion of MDX-010 (Visit 8)

Reason for Change:

Consistent with previous changes.

Page 32, Add the Following Section:

9.1.4. Treatment Phase for Patients Receiving Four Doses – Infusion

The treatment period begins with the first i.v. infusion (Day 1, Visit 3) and continues through the end of Visit 28 (Day 141). Subjects who meet selection criteria will start MDX-010 treatment within 7 +/- 1 days of the pre-entry visit (Visit 2).

The subject will be given i.v. infusions of MDX-010 every 4 weeks for 4 doses. During this treatment phase, the following data will be collected and recorded in the CRF at each scheduled visit (or as indicated):

- MDX-010 infusion information (Visits 3, 9, 15, and 19)
- Vital sign measurements including weight, temperature, and resting systolic and diastolic blood pressure
- Targeted physical examination, including evaluation of heart, lungs, and abdomen (Visits 3, 9, 15, and 19)
- Tetanus booster (Visit 9 only; to be given at the end of the observation period following the second infusion of MDX-010)
- Urine pregnancy test (prior to dosing at Visits 3, 9, 15, and 19)
- Pharmacokinetic assessment (as outlined in Section 9.2; Visits 3 through 7, 9 through 12, and 14 through 23)
- HAHA plasma sample (prior to dosing at Visits 3, 9, 15, and 19)
- Serologic monitoring:
  - CRP, sIL-2r, TNFα, sTNFr, and neopterin will be monitored at Visits 9, 15, and 19
  - ANA and rheumatoid factor will be tested in all subjects at Visits 9, 15, and 19
  - If ANA, rheumatoid factor, anti-cardiolipin Ab, TSH, or anti-thyroid Ab were positive during the screening phase or become positive, then all of these parameters will be monitored at Visits 9, 15, and 19
  - If hepatitis B or C viral loads were detectable during the screening phase, these parameters will be monitored at Visits 9, 15, and 19

- Clinical laboratory tests:
    - Viral load assessments
    - CD4/CD8 counts
    - Immunologic assessments (as outlined in Section 9.1.2.2.)
        - Limited immunologic assessments will be performed at Visits 3, 4, 6, 9, 11, 13, 14, and 18
        - Full immunology assessments will be performed at Visits 8 16, 20, and 23
    - Quantitative tetanus EIA (Visits 3, 8 through 14, 16, 18, 20, and 23)
    - Hematology* (as outlined in Section 9.1.2.1)
    - Clinical Chemistry* (Visits 3, 6, 9, 14, 18, and 23; as outlined in Section 9.1.2.1)
    - Urinalysis (Visits 3, 9, and 14; as outlined in Section 9.1.2.1)
    - * Local CBC and chemistry panel: results to be reviewed and found acceptable prior to the second infusion of MDX-010 (Visit 8)
- Concomitant medications
- Adverse event assessment

Reason for Change:

Addition of dosing regimen required Treatment Phases to be defined for patients receiving 4 doses.

Note: All subsequent sections in Section 9 were renumbered.

Page 30 (currently Page 34) Section 9.1.4 (currently Section 9.1.5), Follow-Up Phase

Change paragraph to read:

Subjects who complete all doses of MDX-010 or who discontinue prematurely for reasons other than death, withdrawal of consent, or are lost to follow-up will return for 2 follow-up evaluations biweekly (Visits 15 and 16 for subjects receiving 2 doses, Visits 24 and 25 for subjects receiving 4 doses). At follow-up visits, subjects will undergo the following:

- Vital sign measurements including weight, height, temperature, and resting systolic and diastolic blood pressure
- Complete physical examination including evaluation of heart, lungs, and abdomen (Visits 16 or 25 only)

- Serologic monitoring:
  - CRP, sIL-2r, TNFα, sTNFr, and neopterin will be monitored at Visits 16 or 25 (Termination)
  - ANA and rheumatoid factor will be tested in all subjects at Visit 16 or 25 (Termination)
  - If ANA, rheumatoid factor, anti-cardiolipin Ab, TSH, or anti-thyroid Ab were positive during the screening phase or become positive, then all of these parameters will be monitored at Visits 16 or 25 (Termination).
  - If hepatitis B or C viral loads were detectable during the screening phase, these parameters will be monitored at Visit 16 or 25 (Termination).
- Quantitative tetanus EIA (Visits 16 or 25 only)
- Hematology (as outlined in Section 9.1.2.1)
- Clinical chemistry (Visits 16 or 25 only; as outlined in Section 9.1.2.1)
- Urinalysis (Visits 16 or 25 only)
- Viral load
- CD4/CD8 counts
- Pharmacokinetic assessment (Visits 16 or 25 only)
- HAHA plasma sample (Visits 16 or 25 only)
- Limited immunologic assessment (Visits 16 or 25 only; as outlined in Section 9.1.2.2)
- Concomitant medications
- Adverse event assessment

Reason for Change:

Additional dosing required additional Termination Visits.

Page 30 (currently Page 34), Section 9.1.5 (currently Section 9.1.6), Termination Visit

Change title to read:

Termination Visit (Visits 16 or 25)

Change paragraphs to read:

A Study Completion/Early Withdrawal visit (Visit 16 for patients receiving 2 doses, Visit 25 for patients receiving 4 doses) will be scheduled for all subjects enrolled in the study. If subjects prematurely withdraw, these procedures will be completed on the day of withdrawal from the study.

If subjects are withdrawn prior to completing 85 or 141 days of the study, the reason for withdrawal will be documented in the CRF and in the source document. All available subjects (withdrawn or completed) will have the following evaluations and procedures completed:

Reason for Change:

Consistent with previous changes.

Page 31 (currently Page 35) Section 9.2, Pharmacokinetic Evaluations

Change section to read:

Blood samples for the analysis of plasma concentrations of MDX-010 will be drawn according to the following schedule:

9.2.1. For Patients Receiving Two Doses

Treatment Phase:

Visits 3 and 9: 30 minutes prior to infusion, 120 minutes and 4 hours post infusion start time 0.

Visits 4, 5, 6, 7, 10, 11, 12, and 14: 1 sample will be obtained.

Follow-up Phase:

Visit 16: 1 sample will be obtained.

9.2.2. For Patients Receiving Four Doses

Treatment Phase:

Visits 3, 9, 15, and 19: 30 minutes prior to infusion, 120 minutes and 4 hours post infusion start time 0.

Visits 4, 5, 6, 7, 10, 11, 12, 14, 16, 17, 18, 20, 21, 22, and 23: 1 sample will be obtained.

Follow-up Phase:

Visit 25: 1 sample will be obtained.

Instructions for the handling, storage, and shipment of pharmacokinetic blood samples are provided in Appendix 1.

Reason for Change:

Consistent with previous changes detailing pharmacokinetic evaluations in patients receiving 2 doses or 4 doses.

Page 31 (currently Page 36) Section 9.3, Clinical Activity Evaluations

Change section to read:

The primary surrogate clinical activity parameter is viral load assessment. A decrease in viral load of at least 0.5 log sustained over 2 or more consecutive determinations will indicate potential clinical activity.

The secondary activity parameter is CD4 count. A CD4 count that increases by more than 20% of entry level sustained over the course of 2 or more consecutive determinations during the trial will indicate potential clinical activity.

Reason for Change:

The Sponsor considers that observed changes (over consecutive readings) are more likely to reflect a "real effect", and not transient fluctuations.

Page 33 (currently Page 38) Section 10.1, Definitions

Change from:

The relationship between the administration of investigational product and the occurrence of the adverse event is described as one of the following:

1 Unrelated (the adverse event is clearly not related to the investigational product)
2 Unlikely (the adverse event is doubtfully related to the investigational product)
3 Possible (the adverse event may be related to the investigational product)
4 Probably (the adverse event is likely related to the investigational product)
5 Definite (the adverse event is clearly related to the investigational product)

To:

The relationship of each adverse event to study drug will be defined as "unrelated", "unlikely", "possible", "probable", or "definite". The Investigator is responsible for determining the study drug relationship for each adverse event that occurs during the study. Assessments are to be recorded on the CRF.

1. Unrelated it is beyond all reasonable doubt that the reported AE was caused by the study drug.
2. Unlikely there is little possibility that the study drug caused the reported AE; and another factor(s) including concurrent illnesses, progression and expression of the disease state, concurrent medications, or a reaction to concurrent medications appear to explain the AE.
3. Possible the reported AE follows a reasonable temporal sequence from administration of the study drug, but could reasonably be explained by the subject's clinical state or concurrent therapy.

4. Probable   the reported AE follows a reasonable temporal sequence from administration of the study drug, and could not reasonably be explained by the subject's clinical state or concurrent therapy.
5. Definite   the reported AE follows an anticipated response and a reasonable temporal sequence to the study drug; and is confirmed by both improvement upon stopping of study drug (withdrawal) and reappearance of the reaction on repeated exposure (rechallenge).

Reason for Change:

Clarification of the terms defining the relationship of the investigational product to the occurrence of the adverse event.

Page 35 (currently Page 40) Section 11.1, Completion

Change paragraph to read:

It will be documented whether or not each subject completed the Treatment Phase of the clinical study (to the end of Visit 10 or Visit 23), how long they were followed, and the reason for withdrawal.

Reason for Change:

Consistent with previous changes.

Page 36 (currently Page 41) Section 12.1, Sample Size Determination

Change paragraph to read:

The sample size of up to 50 patients is based on the trial design for dose escalation and safety evaluation requirements, with up to 30 patients (3 to 6 patients/cohort) at 5 dose levels or treatment regimens and additional 20 patients in the selected 2 cohorts for the evaluation of clinical activity. The primary clinical activity parameter is the proportion of patients who achieve clinical response (a decrease in viral load of at least 0.5 log sustained over two consecutive viral load assessments). It is assumed that none of the patients would have clinical response if they would not have received any therapy and the response rate (RR) for patients in the treatment of 3 mg/kg or 5 mg/kg MDX-010 would be 10%. A one-sample exact Binomial test will be used in testing the alternative hypothesis H_a: $RR_{with\ treatment}$ 10% against the null hypothesis H_0: $RR_{without\ treatment}$ 0%. A sample size of 16 evaluable patients is required to provide more than 80% power in a two-sided exact Binomial test at the significance level of 0.05.

Reason for change:

Consistent with previous changes.

Page 37 (currently Page 42) Section 12.4.2., Extent of Exposure

Change paragraphs to read:

The actual dose of MDX-010 will be summarized by cohort using descriptive statistics.

And add the following section:

12.4.3. Dose Escalation

The probability that dose escalation (PrDE) will be permitted at any stage during MTD determination is a function of the underlying DLT rate (p) at the current dose level. This probability can be calculated as the sum of the binomial probabilities of the following 2 possible outcomes that would permit escalation to occur: (1) no DLT is observed in the first 3 MTD evaluable patients; or (2) one DLT is observed in the first 3 MTD evaluable patients, followed by no DLT observed in 3 additional patients at the same dose level, as expressed by the following formula:

$$Pr_{DE} \quad q^3 + (3pq^2)q^3 \quad q^3 + 3pq^5, \text{ where } q \quad 1 - p.$$

The following table provides the probabilities of dose escalation (PrDE) for a range of underlying DLT rates (p):

Probabilities of Dose Escalation

Underlying DLT Rate (%) P	Probability of Dose Escalation (%) Pr_{DE}
10%	91%
20%	71%
30%	49%
40%	31%
50%	17%
60%	8%
70%	3%
80%	1%
90%	0.1%

Thus, the probability of dose escalation is no more than 3% when the underlying DLT rate is ≥70%, and when the DLT rate is ≤ 10%, the likelihood of escalation is more than 91%.

Those patients who experience at least one DLT will be listed individually by cohort and reason for DLT.

Reason for Change:

Clarification of the statistical methods for extent of exposure and dose escalation.

Note: All sections in 12.4 were subsequently renumbered.

Page 38 (currently Page 43) Section 12.4.3. (currently Section 12.4.4.), Concomitant Medications

Change paragraph to read:

Concomitant medications and significant non-drug therapies will be summarized by cohort using descriptive statistics.

Reason for Change:

Clarification of the statistical methods for summarizing concomitant medications.

Page 38 (currently page 43) Section 12.4.4. (currently Section 12.4.5.), Activity

Change paragraphs to read:

<u>Primary Activity Parameter</u>

The primary clinical activity parameter is the proportion of patients who achieve clinical response in the viral load assessment. A decrease in viral load of at least 0.5 log sustained over two consecutive assessments will indicate potential clinical activity. Viral load will be summarized by cohort using descriptive statistics. A one-sample exact Binomial test will be used in testing the alternative hypothesis Ha: $RR_{with\ treatment}$ 10% against the null hypothesis H0: $RR_{without\ treatment}$ 0%. in the selected 2 cohorts. An exact 95% confidence interval will also be provided.

<u>Secondary Activity Parameters</u>

The secondary activity parameter is CD4 cell counts. A CD4 count that increases by more than 20% of baseline over 2 consecutive assessments will indicate potential clinical activity. CD4 cell counts will be summarized by cohort using descriptive statistics.

Reason for Changes:

Addition of statistical methods for additional dosage groups and consistency with previous changes.

Page 39 (currently Page 44) Section 12.4.5. (currently Section 12.4.6.), Safety

Change paragraph to read:

Vital Signs

The change from baseline to each notable post-baseline visit will be summarized by cohort using descriptive statistics.

Reason for Change:

Clarification of statistical methods for summarizing vital sign measurements.

Page 39 (currently Page 45) Section 12.4.6. (currently Section 12.4.7.), Pharmacokinetic Parameters:

Change paragraph to read:

Plasma concentrations of MDX-010 will be determined by a validated method at the times listed in Tables 1 and 2.

Reason for Change:

Addition of Table 2.

Page 40 (currently Page 45) Section 13.1, MDX-010

Change paragraph to read:

MDX-010 will be administered to the first cohort at a dosage of 0.1 mg/kg, administered as an i.v. infusion at a rate of 1 mL/min (controlled by a volumetric pump) using a 1.2 µm in-line filter for up to 90 minutes, with a 10 cc flush at the end. Subsequent cohorts will be administered MDX-010 at dosages of 1, 3, and 5 mg/kg.

Reason for Change:

Consistent with previous changes.

Clinical Development

Product: MDX-010

Protocol: MDX010-10

A Phase I, Open-label, Dose-escalation Study of MDX-010 Administered Monthly as Immunotherapy in Subjects Infected with Human Immunodeficiency Virus

Document type:	Summary of Changes to Protocol: Amendment 4
Development Phase:	Phase I
Document status:	Final
Release date:	8 September 2005
Number of pages:	4

Property of Medarex, Inc.

Confidential
The information in this document contains trade secrets and commercial information that are privileged or confidential and may not be used, divulged, published, or otherwise disclosed without the written consent of Medarex, Inc. These restrictions on disclosure will apply equally to *all* future information supplied to you which is indicated as *privileged* or *confidential*.

The following changes (indicated in shaded areas) have been made as Amendment 4 to the Protocol for Study MDX010-10:

Page 10, Signature Page for Medarex, Inc.

Change the following:

[redacted]

Signature Date

[redacted]

Signature Date

[redacted]

Signature Date

Approved by:

[redacted]

Signature Date

Reason for change: Change in study personnel and personnel titles.

Page 31 (formerly page 30), Section 9.1.2.2., Pre-entry (Visit 2)

Add the following:

- Clinical Chemistry:
  - Albumin
  - Amylase
  - Serum alkaline phosphatase
  - SGOT (AST)
  - SGPT (ALT)
  - Bilirubin (direct and total)
  - Calcium
  - Creatinine
  - Glucose
  - Lactate dehydrogenase (LDH)
  - Lipase
  - Total protein
  - Urea nitrogen (BUN)
  - Uric acid

Reason for change: Added at the request of the FDA reviewer to evaluate possible cases of pancreatitis.

Page 33 (formerly page 32), Section 9.1.3., Treatment Phase for Subjects Receiving Two Doses - Infusion

Replace:

- Clinical Chemistry* (Visits 3, 6, 9, and 14; as outlined in Section 9.1.2.1)

With:

- Clinical Chemistry* (Visits 3, 6, 9, and 14; as outlined in Section 9.1.2.2)

Reason for change: Consistent with previous change adding amylase and lipase measurements**.**

Page 34 (formerly page 33), Section 9.1.4., Treatment Phase for Subjects Receiving Four Doses – Infusion

Replace:

- Clinical Chemistry* (Visits 3, 6, 8, 9, 14, 15, 18, 19, and 23; as outlined in Section 9.1.2.1)

With:

- Clinical Chemistry* (Visits 3, 6, 8, 9, 14, 15, 18, 19, and 23; as outlined in Section 9.1.2.2)

Reason for change: Consistent with previous change adding amylase and lipase measurements**.**

Page 34, Section 9.1.5., Follow-up Phase

Replace:

- Clinical chemistry (Visits 16 or 25 only; as outlined in Section 9.1.2.1)

With:

- Clinical chemistry (Visits 16 or 25 only; as outlined in Section 9.1.2.1) amylase and lipase to be measured at Visits 15, 21, and 24

Reason for change: Consistent with previous change adding amylase and lipase measurements**.**

Clinical Development

Product: MDX-010

Protocol: MDX010-10

A Phase I, Open-label, Dose-escalation Study of MDX-010 Administered Monthly as Immunotherapy in Subjects Infected with Human Immunodeficiency Virus

Document type:	Clinical Trial Protocol: Amendment 4
Development Phase:	Phase I
Document status:	Final
Release date:	8 September 2005
Number of pages:	59

Property of Medarex, Inc.

Confidential
The information in this document contains trade secrets and commercial information that are privileged or confidential and may not be used, divulged, published, or otherwise disclosed without the written consent of Medarex, Inc. These restrictions on disclosure will apply equally to *all* future information supplied to you which is indicated as *privileged* or *confidential*.

TABLE OF CONTENTS

SYNOPSIS.....5
Signature Page for Medarex, Inc. 10
Signature Page for Investigators 11
ABBREVIATIONS 12
1. INTRODUCTION AND RATIONALE.....14
1.1. Human Immunodeficiency Virus.....14
1.2. CD152 and T-Cell Activation 15
1.3. MDX-010 Specificity and Functional Analyses 16
1.3.1. Multidose Non-Human Primate Toxicity Experience.....16
1.3.2. Prior Clinical Studies in Oncology.....16
1.3.2.1 Completed Phase I Studies.....16
1.3.2.1 Ongoing Studies.....17
1.3.3. Summary of Safety 18
1.4. Rationale for Exploring MDX-010 Therapy in HIV-Infected Subjects 19
2. STUDY OBJECTIVES 21
2.1. Primary Objectives.....21
2.2. Secondary Objectives.....21
3. OVERVIEW OF STUDY DESIGN 21
4. STUDY POPULATION.....22
4.1. Inclusion Criteria.....22
4.2. Exclusion Criteria 23
5. SUBJECT ENROLLMENT 24
5.1. Procedures.....24
5.2. Blinding.....24
6. DOSAGE AND ADMINISTRATION, TOXICITY, AND MANAGEMENT 24
6.1. MDX-010 Dosage 24
6.2. Dose Escalation.....25
6.3. Dose-Limiting Toxicity 26
6.3.1. Management of Diarrhea.....26
6.3.2. Management of HIV Disease 27
6.4. Stopping Rules for Delayed Dose-Limiting Toxicity.....27
6.5. Infusion Reactions.....28
7. COMPLIANCE 29
8. CONCOMITANT THERAPY 29
9. STUDY EVALUATIONS.....29
9.1. Study Procedures by Visit.....29
9.1.1. Overview 29
9.1.2. Screening Phase.....30
9.1.2.1. Prestudy (Visit 1) 30
9.1.2.2. Pre-entry (Visit 2) 31
9.1.3. Treatment Phase for Subjects Receiving Two Doses Infusion 32
9.1.4. Treatment Phase for Subjects Receiving Four Doses Infusion 33
9.1.5. Follow-Up Phase 34
9.1.6. Termination Visit (Visits 16 or 25) 35

9.2. Pharmacokinetic Evaluations....36
9.2.1. For Subjects Receiving Two Doses....36
9.2.2. For Subjects Receiving Four Doses....36
9.3. Clinical Activity Evaluations....36
Immunologic Activity Evaluations....36
9.5. Safety Evaluations....37
10. ADVERSE EVENT REPORTING....37
10.1. Definitions....37
10.2. Serious Adverse Events....39
10.3. Instructions for Rapid Notification of Serious Adverse Events....39
10.3.1. Reporting Responsibility....39
10.3.2. Reporting Procedures....40
10.3.3. Contact Persons and Numbers....40
11. SUBJECT COMPLETION/WITHDRAWAL....40
11.1. Completion....40
11.2. Discontinuation of Treatment....41
12. STATISTICAL METHODS....41
12.1. Sample Size Determination....41
Study Population....42
12.2.1. Safety Population....42
12.2.2. Clinical Activity Population....42
12.2.3. Immunologic Activity Population....42
12.3. Statistical Consideration....42
12.4. Statistical Analysis....42
12.4.1. Demographics and Baseline Characteristics....43
12.4.2. Extent of Exposure....43
12.4.3. Dose Escalation....43
Concomitant Medication....44
12.4.5. Activity....44
12.4.6. Safety....44
12.4.7. Pharmacokinetic Parameters....45
12.5. Statistical Software....46
13. STUDY DRUG INFORMATION....46
13.1. MDX-010....46
13.2. Storage Conditions and Handling of MDX-010....46
14. ETHICAL ASPECTS....47
14.1. Ethics and Good Clinical Practice....47
14.2. Institutional Review Board/Independent Ethics Committee....47
14.3. Informed Consent....47
15. ADMINISTRATIVE REQUIREMENTS....48
15.1. Protocol Amendments....48
15.2. Monitoring Procedures....49
15.3. Recording of Data and Retention of Documents....49
15.4. Auditing Procedures....50
15.5. Handling and Accountability of Investigational Product....51
15.6. Publication of Results....52

15.7. Disclosure and Confidentiality....52
15.8. Discontinuation of Study 52
15.9. Data Management 52
15.9.1. Data Collection....52
15.9.2. Database Management and Quality Control....53
16. REFERENCES 54
17. APPENDICES 56
Appendix 1: Instructions for the Handling, Storage, and Shipment of Blood Samples 57

SYNOPSIS

TITLE:
A Phase I, Open-label, Dose-escalation Study of MDX-010 Administered Monthly as Immunotherapy in Subjects Infected with Human Immunodeficiency Virus

PROTOCOL NUMBER:
MDX010-10

OBJECTIVES:

The primary objective of the study is to establish the safety and tolerability of 2 or 4 doses of MDX-010 in human immunodeficiency virus (HIV)-infected subjects.

Secondary objectives include determination of the pharmacokinetic profile of MDX-010, assessment of any clinical activity to augment an effective immune response against HIV, and assessment for induced cellular and humoral immune responses toward HIV, Candida, and tetanus booster following infusion with MDX-010.

OVERVIEW OF STUDY DESIGN:
This is a Phase I, multicenter, open-label, dose-escalation study to be conducted in HIV-infected subjects. Up to 50 subjects are expected to be enrolled, with 3 to 6 subjects at each of 5 dose levels or dosing regimens. One or 2 cohorts that exhibit safety and evidence of efficacy will be expanded to 16 subjects.

Subjects will receive 2 or 4 doses, administered 4 weeks apart, as detailed in Dosage and Administration. Dosing of subjects at the next higher dose level in each group will not be initiated until 10 days after all subjects in the previous cohort have received both doses. For the first 3 dose levels, cohorts will accrue at least 3 subjects, with 6 subjects in the maximum tolerated dose (MTD) cohort. The MTD dose will be the highest dose where no more than 1 of 6 has experienced a dose-limiting toxicity (DLT). If 1 of 3 subjects experience a DLT, the cohort will be increased to 6 subjects. If 2 or more of the 6 subjects experience DLT, that dose will have exceeded the MTD and a lower dose will accrue to a total of 6 subjects. If this lower dose level is well tolerated and none of the 6 subjects experience a DLT, an additional intermediate dose level may be defined by protocol amendment. If the MTD is not established in the first 6 subjects administered 2 doses of MDX-010 at 3.0 mg/kg, additional subjects (6/group) will be randomized to receive either 4 doses of MDX-010 at 3.0 mg/kg or 2 doses of MDX-010 at 5.0 mg/kg.

STUDY POPULATION:
Subjects whose virus is no longer suppressed by available and tolerable antiretroviral therapy will be enrolled into the study.

DOSAGE AND ADMINISTRATION:
Cohorts of subjects are to be administered MDX-010 at escalating dosage levels of 0.1, 1.0, 3.0, and 5.0 mg/kg/dose, administered as an intravenous (i.v.) infusion. MDX-010 will be administered every 28 days for 2 or 4 doses. If the MTD is not established in the first 6 subjects administered 2 doses of MDX-010 at 3.0 mg/kg, additional subjects (6/group) will be randomized to receive either 4 doses of MDX-010 at 3.0 mg/kg or 2 doses of MDX-010 at 5.0 mg/kg. One or 2 cohorts that exhibit safety and evidence of efficacy will be expanded to 16 subjects.

CLINICAL ACTIVITY EVALUATIONS:
Evaluation of clinical efficacy will include assessments of viral load and CD4 counts.

SYNOPSIS (continued)

IMMUNOLOGIC ACTIVITY EVALUATIONS:

Evaluation of immunologic efficacy will include assessments of changes in frequency and amount of CD4 and CD8 cells with cytokine responses to HIV, Candida, and tetanus antigens; changes in LPA to HIV, Candida, and tetanus antigens; and changes in anti-tetanus toxin antibody levels.

PHARMACOKINETIC EVALUATIONS:

Plasma concentrations of MDX-010 will be assessed prior to and after each infusion, with additional measurements taken throughout the treatment and follow-up periods.

SAFETY EVALUATIONS:

Assessment of safety will be determined by vital sign measurements, clinical laboratory tests, physical examinations, and the incidence and severity of treatment-emergent adverse events.

STATISTICAL METHODS:

The sample size of up to 50 subjects is based on the trial design for dose escalation and safety evaluation requirements, with up to 30 subjects (3 to 6 subjects/cohort) at 5 dose levels or treatment regimens and additional 20 subjects in the selected 2 cohorts for the evaluation of clinical activity. The primary clinical activity parameter is viral load assessment. The other activity parameters include, secondary clinical activity parameter CD4 cell count, immunologic activity parameters CD4 and CD8 cell counts with cytokine response, change in LPA to HIV, Candida, and tetanus antigens, and changes in quantitative anti-tetanus antibody titer. All activity parameters will be summarized using descriptive statistics. A one-sample exact Binomial test will be used for the primary clinical activity parameter in the selected 2 cohorts. The safety parameters include vital signs, clinical laboratory tests, physical examinations, adverse events, diagnostic tests, and special immune function measurements. All safety parameters will be summarized using descriptive statistics. The pharmacokinetic parameters include AUC, C_{max}, and t_{max}. All pharmacokinetic parameters will be summarized using descriptive statistics.

SCHEMA AND TIME AND EVENTS SCHEDULES:

The time and events schedules for this study are shown on the following pages.

Table 1: Time and Events Schedule for Administration of Two Doses

Examination	Screening		Treatment (+/- 1 day)												Follow-Up (+/-3 days)	
**Time point (Days)**	**28 to 21**	**-7**	**1**	**4**	**8**	**15**	**22**	**25**	**29**	**32**	**36**	**43**	**50**	**57**	**71**	**85**
**Visit**	**1**	**2**	**3**	**4**	**5**	**6**	**7**	**8**	**9**	**10**	**11**	**12**	**13**	**14**	**15**	**16**
Informed consent	•															
Inclusion/exclusion criteria[1]	•															
Demographics and medical history[2]	•															
**Clinic Visits and Routine Laboratory Measurements**																
Physical examination	•[3]		•[4]						•[4]							•[3]
Vital sign measurements	•	•	•[5]	•	•	•	•	•	•[5]	•	•	•	•	•	•	•
Electrocardiograph	•															
Chest radiograph	•															
Pregnancy test		•[6]	•[6]						•[6]							
Screening and monitoring serologies	•[7]								•[8]					•[8]		•[8]
Clinical chemistries	•	•[9]	•			•		•[9]	•					•		•
Hematology	•	•[9]	•	•	•	•	•	•[9]	•	•	•	•	•	•	•	•
Urinalysis[10]	•		•						•					•		•
**Study Interventions**																
MDX 010 infusion			•						•							
Tetanus booster									•							
**Standard HIV Laboratory Measurements**																
Plasma HIV RNA	•	•	•	•	•	•	•	•	•	•	•	•	•	•	•	•
CD4/CD8 counts	•	•	•	•	•	•	•	•	•	•	•	•	•	•	•	•
**MDX-010 Pharmacokinetic and HAHA Measurements**																
MDX 010 PK sampling			•[11]	•[12]	•[12]	•[12]	•[12]		•[11]	•[12]	•[12]	•[12]		•[12]		•[12]
Plasma sample for HAHA			•[13]						•[13]							•
**Special Immune Function Measurements**																
Quantitative tetanus EIA	•		•					•	•	•	•	•	•	•		•
T cell subsets flow panel		•	•	•		•		•	•		•		•	•		•
LPA panel		•	•	•		•		•	•		•		•	•		•
Cytokine production (EIA)		•						•						•		
Stored cryopreserved PBMC		•						•						•		
ICC IHC (cryopreserved PBMC)		•						•						•		
**Concomitant medications**	•	•	•	•	•	•	•	•	•	•	•	•	•	•	•	•
**Adverse events**	•[14]	•	•	•	•	•	•	•	•	•	•	•	•	•	•	•

Note: All subjects will have Visit 16 (Termination Visit) evaluations performed at the time of completion or early withdrawal.
Footnotes and Abbreviations:
EIA enzyme immunoassay; PBMC peripheral blood mononuclear cell; HAHA human anti human antibodies;
ICC immunocytochemistry; HC immunohistochemistry

1. To include pretherapy viral load history and pretherapy CD4 count history if known, but not required.
2. Medical history including prior antiretroviral therapy and concurrent medical conditions.
3. Complete physical examination.
4. Target physical examination to include evaluation of heart, lungs, and abdomen.
5. Vital sign measurements will be obtained every 15 mins during the infusion period and every 30 mins for 2 hours following infusion.
6. Serum β HCG pregnancy test within 7 days prior to the first infusion, and urine pregnancy test taken prior to each infusion.
7. Initial screening serologies to include CRP, ANA, VDRL, sIL 2r, TNFα, sTNFr, neopterin, rheumatoid factor, hepatitis B Ag and Ab, and hepatitis C Ab. If ANA or rheumatoid factor are positive, then anti cardiolipin antibody, anti thyroid antibody, and TSH will be reflexively measured. If hepatitis B or C are positive, the relevant quantitative titer will be reflexively measured.
8. All subjects will have ANA and rheumatoid factor tested at Visits 9, 14, and 16 (termination). Subjects with any positive serology will have the full complement of serologies monitored at the remaining Visits (9, 14, and 16).
9. Central laboratory results must be reviewed and the results must be found to be acceptable by the Investigator before additional MDX 010 can be administered.
10. Urinalysis: analysis including microscopic/gross exam and laboratory measurement.
11. PK sampling Intensive: 30 minutes and 120 minutes and 4 hours post infusion start time.
12. PK sampling one sample to be drawn.
13. Sample obtained prior to infusion with MDX 010.
14. During the screening period, baseline signs and symptoms will be collected following signing of the Informed Consent.

Table 2: Time and Events Schedule for Administration of Four Doses

**Examination**	**Screening**		**Treatment (+/- 1 day)**																					**Follow-Up (+/-3 days)**	
**Time point (Days)**	**–28 to –21**	**-7**	**1**	**4**	**8**	**15**	**22**	**25**	**29**	**32**	**36**	**43**	**50**	**53**	**57**	**64**	**71**	**78**	**85**	**92**	**99**	**106**	**113**	**127**	**141**
**Visit**	**1**	**2**	**3**	**4**	**5**	**6**	**7**	**8**	**9**	**10**	**11**	**12**	**13**	**14**	**15**	**16**	**17**	**18**	**19**	**20**	**21**	**22**	**23**	**24**	**25**
Informed consent	•																								
Inclusion/exclusion criteria[1]	•																								
Demographics and medical history[2]	•																								
**Clinic Visits and Routine Laboratory Measurements**																									
Physical examination	•[3]		•[4]						•[4]						•[4]				•[4]						•[3]
Vital sign measurements	•	•	•[5]	•	•	•	•	•	•[5]	•	•	•	•	•	•[5]	•	•	•	•[5]	•	•	•	•	•	•
Electrocardiograph	•																								
Chest radiograph	•																								
Pregnancy test		•[6]	•[6]						•[6]						•[6]				•[6]						
Screening and monitoring serologies	•[7]								•[8]						•[8]				•[8]						•[8]
Clinical chemistries	•	•[9]	•			•		•[9]	•					•[9]	•			•[9]	•				•		•
Hematology	•	•[9]	•	•	•	•	•	•[9]	•	•	•	•	•	•[9]	•	•	•	•[9]	•	•	•	•	•	•	•
Urinalysis[10]	•		•						•					•											•
**Study Interventions**																									
MDX-010 infusion			•						•						•				•						
Tetanus booster									•																
**Standard HIV Laboratory Measurements**																									
Plasma HIV RNA	•	•	•	•	•	•	•	•	•	•	•	•	•	•	•	•	•	•	•	•	•	•	•	•	•
CD4/CD8 counts	•	•	•	•	•	•	•	•	•	•	•	•	•	•	•	•	•	•	•	•	•	•	•	•	•
**MDX-010 Pharmacokinetic and HAHA Measurements**																									
MDX-010 PK sampling			•[11]	•[12]	•[12]	•[12]	•[12]		•[11]	•[12]	•[12]	•[12]		•[12]	•[11]	•[12]	•[12]	•[12]	•[11]	•[12]	•[12]	•[12]	•[12]		•[12]
Plasma sample for HAHA			•[13]						•[13]						•[13]				•[13]						•
**Special Immune Function Measurements**																									
Quantitative tetanus EIA	•		•					•	•	•	•	•	•	•		•		•		•			•		•
T cell subsets – flow panel		•	•	•		•		•	•		•		•	•		•		•		•			•		•
LPA panel		•	•	•		•		•	•		•		•	•		•		•		•			•		•
Cytokine production (EIA)		•						•								•				•			•		
Stored cryopreserved PBMC		•						•								•				•			•		
ICC-IHC (cryopreserved PBMC)		•						•								•				•			•		
**Concomitant medications**	•	•	•	•	•	•	•	•	•	•	•	•	•	•	•	•	•	•	•	•	•	•	•	•	•
**Adverse events**	•[14]	•	•	•	•	•	•	•	•	•	•	•	•	•	•	•	•	•	•	•	•	•	•	•	•
Footnotes may be found on the following page.																									

Table 2: Time and Events Schedule for Additional Dosing (continued)

Footnotes:

Note: All subjects will have Visit 28 (Termination Visit) evaluations performed at the time of completion or early withdrawal.

Footnotes and Abbreviations:

EIA = enzyme immunoassay; PBMC = peripheral blood mononuclear cell; HAHA = human anti-human antibodies;
ICC = immunocytochemistry; HC = immunohistochemistry

1. To include pretherapy viral load history and pretherapy CD4 count history if known, but not required.
2. Medical history including prior antiretroviral therapy and concurrent medical conditions.
3. Complete physical examination.
4. Target physical examination to include evaluation of heart, lungs, and abdomen.
5. Vital sign measurements will be obtained every 15 mins during the infusion period and every 30 mins for 2 hours following infusion.
6. Serum β-HCG pregnancy test within 7 days prior to the first infusion, and urine pregnancy test taken prior to each infusion.
7. Initial screening serologies to include CRP, ANA, VDRL, sIL-2r, TNFα, sTNFr, neopterin, rheumatoid factor, hepatitis B Ag and Ab, and hepatitis C Ab. If ANA or rheumatoid factor are positive, then anti-cardiolipin antibody, anti-thyroid antibody, and TSH will be reflexively measured. If hepatitis B or C are positive, the relevant quantitative titer will be reflexively measured.
8. All subjects will have ANA and rheumatoid factor tested at Visits 9, 15, 19, and 25 (termination). Subjects with any positive serology will have the full complement of serologies monitored at the remaining Visits (9, 15, 19, and 25).
9. Central laboratory results must be reviewed and the results must be found to be acceptable by the Investigator before additional MDX-010 can be administered.
10. Urinalysis: analysis including microscopic/gross exam and laboratory measurement.
11. PK sampling – Intensive: -30 minutes and 120 minutes and 4 hours post-infusion start time.
12. PK sampling – one sample to be drawn.
13. Sample obtained prior to infusion with MDX-010.
14. During the screening period, baseline signs and symptoms will be collected following signing of the Informed Consent.

Signature Page for Medarex, Inc.

Protocol No.: MDX010-10

Title: A Phase I, Open-label, Dose-escalation Study of MDX-010 Administered Monthly as Immunotherapy in Subjects Infected with Human Immunodeficiency Virus

Reviewed by:

[redacted]

Signature Date

[redacted]

Signature Date

[redacted]

[redacted] Date

Approved by:

[redacted]

[redacted] Date

[redacted]

Signature Page for Investigators

Protocol No.: MDX010-10

Title: A Phase I, Open-label, Dose-escalation Study of MDX-010 Administered Monthly as Immunotherapy in Subjects Infected with Human Immunodeficiency Virus

I have read this protocol and agree that it contains all necessary details for carrying out this study. I will conduct the study as outlined herein and will complete the study within the time designated, in accordance with all stipulations of the protocol and in accordance with Good Clinical Practices, local regulatory requirements, and the Declaration of Helsinki.

I will provide copies of the protocol and all pertinent information to all individuals responsible to me who assist in the conduct of this study. I will discuss this material with them to ensure that they are fully informed regarding the study drug and the conduct of the study.

I will use only the informed consent form approved by the Institutional Review Board/Independent Ethics Committee (IRB/IEC) and will fulfill all responsibilities for submitting pertinent information to the IRB/IEC responsible for this study.

I agree that the sponsor or its representatives shall have access to any source documents from which case report form information may have been generated.

I further agree not to originate or use the name of Medarex, Inc., or MDX-010 in any publicity, news release, or other public announcement, written or oral, whether to the public, press, or otherwise, relating to this protocol, to any amendment to the protocol, or to the performance of this protocol, without the prior written consent of Medarex, Inc.

______________________	______________________	__________
Investigator Name (print)	Signature	Date

ABBREVIATIONS

**Abbreviation**	**Term**
Ab	antibody
ADCC	antibody-dependent cellular toxicity
AE	adverse event
AIDS	acquired immunodeficiency syndrome
ANOVA	Analysis of Variance
APCs	antigen-presenting cells
ART	antiretroviral therapy
ATI	analytic treatment interruption
AUC	area under the time versus concentration curve
BMT	bone marrow transplant
BUN	blood urea nitrogen
CBC	complete blood count
cDNA	complementary deoxyribonucleic acid
C_{max}	maximum plasma concentration observed post-dose
CRF	Case Report Form
CRP	C-reactive protein
CRO	contract research organization
CTC	Common Toxicity Criteria
CTL	cytotoxic T-lymphocytes
DAIDS	The NIH/NIAID Division of AIDs
DLT	dose limiting toxicity
ECG	electrocardiogram
ELISA	enzyme-linked immunosorbant assay
EIA	enzyme immunoassay
GVH	graft versus host
GVL	graft versus leukemia
HAART	highly-active antiretroviral treatment
HAHA	human anti-human antibodies
HCG	human chorionic gonadotropin
HIV	human immunodeficiency virus
HPF	high-powered field
HuMab®	human monoclonal antibody
ICC	immunocytochemistry
IHC	immunohistochemistry
i.v.	intravenous
IRB/IEC	Institutional Review Board/Independent Ethics Committee
LDH	lactate dehydrogenase
LPA	lymphocyte proliferation assay
LTNP	long term non-progressors
mAbs	monoclonal antibodies
MTD	maximum tolerated dose

ABBREVIATIONS (continued)

Abbreviation	Term
(N/Nt)RTIs	nucleoside/nucleotide reverse transcriptase inhibitors
NNRTI	non-nucleoside reverse transcriptase inhibitors
PBMC	peripheral blood mononuclear cell
Pis	protease inhibitors
RNA	ribonucleic acid
SAE	serious adverse event
SAS	Statistical Analysis System
SGOT (AST)	serum glutamic oxaloacetic transaminase (aspartate transaminase)
SGPT (ALT)	serum glutamic pyruvate transaminase (alanine transaminase)
SHIV	simian-human immunodeficiency syndrome
SIL-R	soluble interleukin 2 receptor
SOPs	Standard Operating Procedures
STI	structured/scheduled treatment interruptions
sTNFr	soluble tumor necrosis factor α
TET	tetanus toxoid
t_{max}	time at which the maximum concentration (C_{max}) occurs
TCR	T-cell receptors
TNFα	tumor necrosis factor α
VL	viral load
WHO	World Health Organization

1. INTRODUCTION AND RATIONALE

1.1. Human Immunodeficiency Virus

Chronic infection with human immunodeficiency virus (HIV) is characterized by progressive depletion of CD4 cells and loss of cellular immune responses that control infections by previously exposed and/or environmentally common pathogens, and results in acquired immunodeficiency, AIDS. One of the first cellular immune responses that is lost after infection, well before advanced CD4 depletion occurs, is the CD4 proliferative response directed toward HIV antigens.[1] Although CD8 cells that bind HIV epitopes are readily detectable throughout the course of HIV infection, they do not completely suppress viral replication, in part because of viral mutations that evade peptide specific immune responses, but also because CD8 function and cytotoxic activity are impaired.[2,3] HIV-specific CD4 proliferative responses are minimal or absent in uncontrolled chronic infection. The proliferative response to HIV antigen does not recover to a significant degree even after several years of successful highly-active antiretroviral treatment (HAART) in chronically infected subjects.[4] In the minority of patients who control HIV without the need for antiretroviral therapy, designated as long term non-progressors (LTNPs), CD4 proliferation in response to HIV antigens is easily detectable and provides the help for HIV-specific CD8 cytotoxic T-lymphocytes (CTL) to suppress viral replication.[5] It was initially thought that the absence of HIV-specific CD4 proliferation in chronic infection was due to depletion of reactive CD4 cells by direct HIV infection. Subsequent experiments however, demonstrate that CD4 cells with T-cell receptors (TCR) that recognize HIV antigens are still present, but their production of cytokines in response to antigen is reduced, they do not proliferate, and do not provide adequate help.[6] Thus, one view of an important immunological defect in chronic HIV infection is a weak or anergized CD4 response to HIV antigens that fails to adequately mobilize the CD8 cytotoxic T cells necessary to limit viral replication. Interventions that strengthen these weak and/or anergic cellular immune responses to the virus may, therefore, be beneficial.

CD152 (CTLA-4) is a T-cell surface molecule induced upon T-cell activation; CD152 competes with CD28, the T-cell co-receptor, for binding to the cognate CD80/86 (B7 family) receptors present on antigen-presenting cells (APC). CD28 binding to APC is required to deliver costimulatory signals for the expansion of a cellular immune response, while CD152 signalling results in an inhibitory response (see Section 1.2). An increase in the circulating level of CD152-positive CD4 and CD8 cells in the blood of HIV-infected subjects compared with uninfected controls has been reported, and the amount of CD152-positive cells correlated with the level of HIV plasma viremia.[7,8]

Enhanced expression of CD152 may thus contribute to inhibition of the cellular immune response to HIV, and therefore, blockade of the CD152 CD80/86 inhibitory interactions may enhance the immune response of CD4 and CD8 cells to HIV antigens.[7] In addition, a recent report suggests that export of the HIV envelope protein to the cell surface in infected cells occurs via the secretory pathway utilized by CD152, and that expression of HIV envelope on the cell surface is accompanied by increased surface expression of CD152.[9] Moreover, other reports demonstrate that signalling via CD152 leads to increased expression of CCR5 and enhanced susceptibility of primary lymphocytes to R5 tropic strains of HIV.[10] Thus, increased expression of CD152 on T cells may be directly both caused by and facilitate HIV replication in individual lymphocytes, as well as downmodulate productive immune responses to the virus.

1.2. CD152 and T-Cell Activation

Advances in understanding of the mechanisms that regulate T-cell activation have allowed the rational design of new strategies for immunotherapy of chronic diseases such as tumors and persistent infections. It has been known for some time that engagement of the T-cell antigen receptor by itself is not sufficient for full T-cell activation; a second co-stimulatory signal is required for induction of IL-2 production, proliferation and differentiation to effector function of naive T cells. Abundant data now indicate that the primary source of this co-stimulation is mediated by engagement of CD28 on the T cell surface by members of the B7 family (CD80 and CD86) on the antigen-presenting cell.[11] Expression of B7 has been shown to be limited to "professional" antigen-presenting cells; that is, specialized cells of the hematopoietic lineage, including dendritic cells, activated macrophages, and activated B cells. It has been suggested that this sharply defined restriction of B7 expression is a fail-safe mechanism for maintenance of peripheral T-cell tolerance, insuring that T cells are only stimulated by appropriate antigen-presenting cells.[12]

In the past few years it has become apparent that co-stimulation is even more complex than originally thought. After activation, T cells express CD152 (CTLA-4), a close homologue to CD28. CD152 binds members of the B7 family with a much higher affinity than CD28.[13,14] Although there was initially some controversy as to the role of CD152 in regulating T cell activation, it has become clear that CD152 down-regulates T cell responses.[15] This was initially suggested by the following in vitro observations: (1) blockade of CD152/B7 interactions with antibody enhanced T cell responses; (2) cross-linking of CD152 with CD3 and CD28 inhibited T cell responses; and (3) administration of antibodies to CD152 in vivo enhanced the immune response to peptide antigens or superantigens in mice.[16 19 789] The main evidence for an inhibitory rather

than agonist role for CD152 was that anti-CD28 antibodies blocked responses in vivo; this effect was dominant over the enhancing effect of anti- CD152 antibodies.[18]

Perhaps the most convincing demonstration of the down-regulatory role of CD152 came from examination of mice with a null mutation.[20 22] CD152 knockout mice appear to have spontaneously activated T cells evident at approximately 1 week after birth, followed by rampant lymphoproliferation and lymphadenopathy. These mice die at approximately 3 weeks of age, either as a result of polyclonal T cell expansion and tissue destruction or as a result of toxic shock resulting from lymphokine production by the T cells. Since thymocyte differentiation and selection proceed normally in CD152-deficient mice, the rampant T cell expansion that occurs in the mice indicates that CD152 plays a critical role in down-regulating T cell responses in the periphery.[20]

1.3. MDX-010 Specificity and Functional Analyses

MDX-010 is a human IgG1 anti- CD152 monoclonal antibody (mAb). In vitro studies were performed with mAb MDX-010 to demonstrate that it is specific for CD152, actively inhibits CD152 interactions with B7.1 and B7.2, and does not show any cross-reactivity with 4 human B7.1 and B7.2 negative cell lines. These results show that human mAb MDX-010 is active, specific, and appropriate for evaluation in human clinical trials.

1.3.1. Multidose Non-Human Primate Toxicity Experience

In vivo primate studies were performed to further determine the safety of MDX-010. Specific studies included:

- Cynomologus monkeys (4 animals per dose level) tolerated 3 bolus i.v. doses of 3, 10, and 30 mg/kg on Days 1, 4, and 7 without any significant clinical findings
- Cynomologus monkeys (4 males and 4 females) tolerated 2 monthly i.v. injections of 10 mg/kg without any signs of clinical toxicity.
- In a chronic toxicology study, cynomologus monkeys (2 males and 2 females) tolerated 5 monthly i.v. injections of 10 mg/kg without any signs of clinical or pathological toxicity. In addition, 3 male and 3 female animals tolerated 5 monthly i.v. injections of 10 mg/kg in combination with a vaccine (GM-CSF secreting human melanoma cell line).

1.3.2. Prior Clinical Studies in Oncology

1.3.2.1 Completed Phase I Studies

Two disease-specific, open-label, multicenter, Phase I trials have been completed. These studies evaluated a single fixed dose of MDX-010 (3 mg/kg) in order to determine the pharmacokinetics and potential responsiveness of tumors to MDX-010 administration.

MDXCTLA4-01 was performed in 14 subjects with progressive, metastatic, hormone-refractory prostate cancer. MDXCTLA4-02 was performed in 17 subjects with surgically unresectable Stage III or IV malignant melanoma.

Plasma levels were consistent with the expected result of non-linear pharmacokinetics with a prolonged half-life. There was evidence of detectable plasma concentrations up to 4 months after administration in some subjects.

Infusions of MDX-010 were well tolerated. Initial mild infusion reactions were seen, including diaphoresis, nausea, flushing, and hypotension. These were rapidly reversible, and were similar to infusions with many other antibodies. A number of subjects developed a macular, erythematous rash, which was only rarely symptomatic and did not require therapeutic intervention. These rashes persisted for several months. One subject with prostate carcinoma developed moderately severe pruritus and rash (Grade 3). Other adverse events considered related to the infusion of MDX-010 include abdominal pain, fever, fatigue, rhinitis, arthralgia, myalgia, cough, retinal pigment changes, hypotension, leukocytosis, anemia, thrombocytosis, diarrhea, vomiting, diverticulosis, ataxia, facial twitching, impaired speech, vitiligo, and positive ANA.

Although these studies were designed for safety assessments, some preliminary evidence of efficacy was observed. There was objective evidence of tumor regression in 2 of 17 subjects treated in the melanoma trial and in 2 of 14 subjects in the prostate carcinoma trial.[23,24]

1.3.2.1 Ongoing Studies

Multiple dosing of MDX-010 is currently being tested in 5 Phase II trials in oncology. In one ongoing melanoma trial, an aggressive regimen of q 3 week administration of a melanoma vaccine along with MDX-010 at 3 mg/kg (a more frequent dosing regimen than any dose proposed for this trial) is being studied. This ongoing trial has noted episodes of diarrhea in 2 of 10 subjects receiving at least the second of q 3 week infusion with melanoma vaccine. These episodes were either self-limited or easily controlled with a short steroid taper. Colonic biopsies obtained from these 2 subjects revealed lymphocytic infiltrates in the absence of any documented infectious pathogen. One case of transaminase elevation and 1 case of possible hypophysitis have also been observed. The transaminase elevation resolved, largely on its own, and the subject with pituitary suppression is currently being controlled with replacement therapy. The dosing regimen on this trial has been amended; the initial dose of MDX-010 is administered at 3 mg/kg with subsequent doses at 1 mg/kg.

It is logical that MDX-010 may elicit autoimmune type adverse events, perhaps similar to that seen in GVH following allogeneic bone marrow transplant (BMT). Interestingly, each of the instances of autoimmune-type adverse event phenomena in the melanoma trial have been associated with clinical responses of regression of the underlying tumor. This is also similar to BMT for lymphoid malignancy where a successful GVL often correlates with the occurrence of GVH.[25] An expert panel of consultants has been convened to review these events. The diarrhea/inflammatory bowel reaction likely reflects the authentic bioactivity of this agent, and subjects will be closely monitored for this effect.

1.3.3. Summary of Safety

CD152 blockade in the absence of intentional immunization appears to be benign. Animal studies demonstrated that only T cells responding to specific immunogens seem to be affected, even under conditions of rather chronic treatment. The only side effects observed in human trials have been tissue-specific autoimmunity in the melanoma and prostate models, and in the intense melanoma vaccine trial, 3 cases of inflammatory diarrhea, 1 case of resolved hepatitis, and 1 case of medically stable hypophysitis. Interestingly, clinical immunotherapy of melanoma in human subjects has been associated with development of vitiligo-like lesions which may be an unavoidable consequence of successful treatment.

Given the lethality of the CD152 null mutation in mice, the apparently benign effects of anti-CD152 treatment could have several explanations. It may be that epitopes recognized by the antibody are not available on the entire intracellular pool of CD152. For this, or for some other reason, the functional block by antibody is not complete. Alternatively, complete blockade of CD152 in the adult may not have the same consequences as for the developing immune system.

The overall safety testing for mAb MDX-010 to date indicates there are limited and manageable concerns for safety. This is based on the following criteria:

- MDX-010 is a fully human mAb expected to have minimal immunogenicity in humans.
- The specificity of MDX-010 binding to CD152 was demonstrated using recombinant proteins, transfected cell lines, and frozen human tissues.
- No unanticipated cross-reactivity was observed in 36 tissues from 3 separate donors.
- MDX-010 does not mediate ADCC or CDCC of activated T cells in vitro.
- MDX-010 was well tolerated and not associated with any toxicity in preclinical primate toxicology studies.

- MDX-010 was well tolerated in 2 Phase I studies in humans.
- MDX-010 has been associated with autoimmune-like effects mostly in an intensively dosed study in subjects with melanoma, each of these subjects also showed marked clinical responses.

Additionally, each clinical lot of mAb MDX-010 will be tested in mice and guinea pigs for general safety according to the testing specifications described in the Code of Federal Regulations 21, Part 610.11.

1.4. Rationale for Exploring MDX-010 Therapy in HIV-Infected Subjects

MDX-010 is a human antibody to CD152 that blocks the interaction of CD152 with CD80/86. Binding by MDX-010 results in disinhibition of cellular immune responses that would otherwise be downmodulated after the induction of CD152 and its competition with CD28 for binding to the CD80/86 receptors on APCs. Experimental and clinical data indicate that increased expression of CD152 is present in the T cells of HIV-infected subjects, and this excess expression may interfere with the elaboration of an effective immune response that can control HIV replication and disease progression.[7,8] Signalling via CD152 may increase susceptibility of primary lymphocytes to macrophage tropic (R5) strains of HIV.[10] Blockade of CD152 by MDX-010 may therefore augment an effective immune response to HIV in infected subjects. Two Phase I trials have been completed with MDX-010 in subjects with prostate and melanoma cancers. MDX-010 was well tolerated at single doses of 3 mg/kg, displayed a half life of 12 to 15 days, compatible with monthly injections to maintain effective blood levels, and with preliminary indications of efficacy. It is currently undergoing Phase II evaluations in a variety of cancers, with both multiple dosing and in combination with cancer vaccines to assess its utility to enhance immune responses to tumors and induce their regression.

Subjects whose antiretroviral therapy is no longer suppressing viremia are to be enrolled in this study. These will be HAART-experienced subjects with at least 2 episodes of virologic failure, defined as documented virus breakthrough while on therapy on at least 2 occasions, and will have virus with documented resistance to drugs in each therapeutic class (RTIs, NNRTIs, and PIs) and also to the newer fusion inhibitor if available. Despite breakthrough viremia, subjects may still benefit from their regimen and preserve their CD4 counts, either because the level of viremia on-therapy is lower than that off-therapy, or the breakthrough virus may not be as “fit” and pathogenic as “wild-type” virus.[26] Subjects who are no longer taking previously failed HAART due to intolerance or to regimen fatigue will also be permitted. Ultimately these subjects will require additional therapies to prevent progression of disease, and are in need of new treatment modalities. Therefore these subjects are appropriate for initial studies with MDX-010.

Our current understanding of the viral set point in the absence of HAART is that it reflects the balance between the amount of viral antigenic challenge required to drive the immune system and the control that the immune response can exert.[1] A breakthrough viremia may still be significantly lower than the subject’s natural set point, therefore, the antigenic drive provided by circulating virus may be below that required to maximally activate HIV-specific T cells. Blockade of CD152 in this group may decrease the threshold required to activate an effective antiviral cellular immune response, drive down viremia, preserve or improve their CD4 counts, and thereby delay disease progression.

The principal hypothesis for a clinical trial of MDX-010 in these HIV-infected subjects is that enhanced expression of CD152 occurs in the context of HIV replication, and interferes with the generation of an effective immune response to control the virus. Blockade by MDX-010 of the inhibitory CD152 CD80/86 interactions will enhance the immune response to the virus and manifest by an improved level of control of HIV viremia.

2. STUDY OBJECTIVES

2.1. Primary Objectives

1. To establish the safety and tolerability of multiple doses of MDX-010 in HIV-infected subjects.

2.2. Secondary Objectives

1. To determine the pharmacokinetic profile of MDX-010 in HIV-infected subjects
2. To assess for any clinical activity that augments an effective immune response against HIV.
3. To assess for induced cellular and humoral immune responses toward HIV, candida, and tetanus booster after MDX-010 infusion.

3. OVERVIEW OF STUDY DESIGN

This is a Phase I, open-label, multicenter, dose-escalation study in HIV-infected subjects. The study is designed to determine the safety and tolerability profile of 2 or 4 doses of MDX-010, a human mAb. It is also intended to characterize the pharmacokinetic profile of MDX-010 administered i.v. once every 4 weeks for 2 or 4 doses, to assess any preliminary clinical activity to augment an effective immune response against HIV, and to assess for induced cellular and humoral immune responses toward HIV, Candida, and a tetanus booster.

During the first visit, after obtaining informed consent, screening evaluations will begin. Subject eligibility will be determined by the inclusion and exclusion criteria on prestudy evaluation listed in Section 4.

During the treatment phase, subjects will be required to visit the Investigator's office or clinic every 3 to 7 days for 8 weeks (subjects receiving 2 doses) or 16 weeks (subjects receiving 4 doses) for administration of study drug, pharmacokinetic sampling, vital sign measurements, physical examinations, clinical laboratory testing, viral load and immunologic assessments, and/or the collection of adverse events.

All subjects will receive a tetanus booster at Visit 9 (Day 29) of the study, to be given at the end of the observation period following the second infusion of MDX-010.

Evaluation of clinical activity will include assessments of viral load and CD4 counts. Evaluation of immunologic activity will include assessments of changes in immune response to recall and HIV antigens (e.g. frequency and amount of CD4 and CD8 cells

with cytokine responses and lymphocyte proliferation to HIV, candida, and tetanus antigens). Evaluation of safety and tolerability will include evaluation of adverse events and drug-induced toxicity, physical examination including vital sign measurements, ECG (if indicated), urinalysis, and blood sampling for hematological and biochemical assessments.

4. STUDY POPULATION

Subjects with active HIV infection, who are on antiretroviral therapy and who have detectable viremia (viral load by RT-PCR [VL] between 1,000 and 100,000 copies/mL), and CD4 > 100 cells/mm^3 will be enrolled into the study. A maximum of 50 subjects (3 to 6 subjects per group, with 16 subjects in 1 or 2 cohorts that demonstrates safety and evidence of efficacy) will be enrolled. The specific inclusion and exclusion criteria for enrolling subjects in this study are described in the following sections.

4.1. Inclusion Criteria

All subjects must meet the following criteria on prestudy examination (within 28 days prior to study drug administration) to be eligible to participate in the study:

1. No significant organ compromise or functional medical disorder.
2. At least 18 years of age.
3. Current HAART regimen stable for at least 3 months, and VL between 1,000 and 100,000 copies/mL on at least 2 occasions during this period, with the measurements within 0.5 log variation of each other (including screening value and 1 value at least 45 days prior to screening).
1. CD4 count ≥ 100 cells/mm^3 for the previous 6 months on at least 2 determinations (including screening value and one value at least 90 days prior to screening).
5. Current ART regimen follows at least 2 previous changes for documented virologic failure (breakthrough viremia while adherent to a regimen containing ≥ 3 antiretroviral agents, and exclusive of prior mono or dual therapy), and documented resistance tests demonstrate the presence of at least 1 mutation to each major therapeutic class of ART [(N/Nt)RTI, NNRTI, PI]. Subjects failing fusion inhibitors do not need resistance tests. If a therapeutic class of agents is not being used for reasons of intolerance, then documentation of the signs of intolerance can substitute for resistance mutations to that class. Therefore, subjects who are not currently taking antiretroviral therapy for reasons of resistance, intolerance, or regimen fatigue may also be enrolled.
6. Willing to remain on current therapy for 12 weeks of study (unless toxicity or safety issues indicate otherwise).

7. Screening laboratory values must meet the following criteria:
   - Hemoglobin ≥ 8.0 g/dL
   - WBC ≥ 2000 cells/mm^3
   - Neutrophils ≥ 750 cells/mm^3
   - Platelets ≥ 100,000 cells/mm^3
   - ALT, AST, and alkaline phosphatase ≤ 5 times ULN
   - Serum creatinine < 2 x ULN
   - Proteinuria < 1 g/day

4.2. Exclusion Criteria

Subjects who exhibit any of the following conditions at screening will not be eligible for admission into the study:

1. Any experimental treatment less than 4 months prior to screening.
2. Initiation of any new medications that might reasonably affect the immune response or viral load less than 4 weeks prior to screening.
3. Tetanus booster immunization within 2 months of initial screening procedures, or a history of anaphylaxis or severe local reaction to the tetanus vaccine.
4. History of autoimmune disease (e.g., thyroid, lupus, rheumatoid arthritis, autoimmune hepatitis, IBD / Crohn's disease) at risk for recurrence.
5. Current malignancy, except Stage A or B cervical carcinoma or basal cell carcinoma.
6. Any active chronic medical condition that, in the view of the Investigator, makes the subject a poor candidate for participation in the clinical trial. For example, poorly controlled hypertension, diabetes mellitus, congestive heart failure, or arrhythmias.
7. Active psychiatric disease that would interfere with participation in the trial.
8. Acute illness or infection.
9. Chronic viral hepatitis, due to hepatitis B or hepatitis C, with:
   - Current treatment with interferon or Ribavirin; or
   - Hepatitis B DNA viral load > 25 pg/cc; or
   - Hepatitis C RNA viral load > 20,000 IU/cc
10. Currently undergoing treatment or prophylaxis for tuberculosis infection.
11. Chronic active infectious disease (other than HIV) such as chronic renal infection, chronic chest infection with bronchiectasis or sinusitis.
12. Known active drug or alcohol abuse that would interfere with participation in the trial.

1. Pregnant or breastfeeding women; women of childbearing potential must be using an acceptable barrier method of birth control and prevention of venereal disease and must agree to continue using this method during the course of the study.

14. Sexually active men must agree to use an acceptable barrier method of birth control and prevention of venereal disease.

5. SUBJECT ENROLLMENT

5.1. Procedures

Screening numbers will be assigned by the Principal Investigator/designee at each study site as soon as the subject has signed the informed consent. Subjects that are screened and do not meet all entry criteria will be entered into a screening log; data collected on screening failures will not be entered in the clinical database. Once assigned, numbers for any screening failures, non-treated, non-evaluable, or discontinued subjects will not be re-used.

The study center will contact the sponsor once a subject is determined to be eligible for enrollment for treatment assignment. Subjects who meet all eligibility requirements will be assigned to the next available dose level, as determined by Medarex, Inc.

To assign the subjects to the 2 cohorts consisting of 16 patients for evaluation of clinical activity, the Biostatistics group at Medarex, Inc. will provide a centralized randomization list to Clinical Operations using SAS procedure PROC PLAN. To keep the balance of the treatment allocation, the randomization scheme will be generated using block size of 4 and will not be stratified by site. The randomization list includes block number, patient number, and treatment group. The Biostatistics group will provide an overall randomization list (both electronic copy and hard copy) to Clinical Operations.

5.2. Blinding

This is an open-label study and blinding is not applicable.

6. DOSAGE AND ADMINISTRATION, TOXICITY, AND MANAGEMENT

6.1. MDX-010 Dosage

Beginning Day 1 (Visit 3), MDX-010 will be administered as a 90 minute i.v. infusion, using a 1.2 μm in-line filter and a volumetric pump, at an initial dosage of 0.1 mg/kg/dose; it is **not** to be administered as an i.v. push or bolus injection. Infusions of MDX-010 will be administered every 28 days for 2 or 4 doses. Succeeding dosage

levels will include 1.0, 3.0, and 5.0 mg/kg/dose. If the MTD is not established in the first 6 subjects administered 2 doses of MDX-010 at 3.0 mg/kg, additional subjects (6/group) will be randomized to receive either 4 doses of MDX-010 at 3.0 mg/kg or 2 doses of MDX-010 at 5.0 mg/kg. One or 2 cohorts that exhibit safety and evidence of efficacy will be expanded to 16 subjects.

Therapy will be discontinued for the following toxicities (if deemed drug-related):

- Any Grade 3 infusion reaction, including bronchospasm, hypoxia, hypotension, hypertension, tachycardia, or bradycardia. The infusion is to be immediately discontinued and no additional MDX-010 will be administered.
- Any Grade 3 or greater clinically apparent autoimmunity, or any Grade 2 or greater clinical autoimmunity of critical organs, including lung, heart, kidney, bowel, bone marrow, or central nervous system (including the eye).

MDX-010 dosing is to be delayed for any persistent Grade 2 autoimmunity of other systems that has not resolved by the time additional dosing is scheduled. Once the toxicity has reduced to Grade 1 or less, the dosing regimen is to be resumed. If the persistent autoimmunity worsens (to Grade 2 or 3), treatment is to be discontinued. A delay of greater than 3 weeks in dosing will exclude further therapy.

6.2. Dose Escalation

Dose escalation will start at 0.1 mg/kg; succeeding dose levels will include 1.0, 3.0, and 5.0 mg/kg. Cohorts of 3 to 6 subjects will be treated at each of the 5 dose levels or treatment regimens. Subjects will receive MDX-010 at 4-week intervals for 2 or 4 doses.

For the first 3 cohorts, in the absence of dose-limiting toxicity (DLT) in the first 3 subjects at each dosage level, enrollment in the next dosage level will begin 10 days after the third subject has tolerated the second dose. Cohorts will accrue at least 3 subjects, with 6 subjects in the MTD cohort. The MTD dose will be the highest dose where no more than 1 of 6 subjects has experienced DLT. If one subject experiences a DLT, the cohort will be increased to 6 subjects. If 2 or more of the 6 subjects experience DLT, that dose will have exceeded the MTD and a lower dose level will accrue to a total of 6 subjects. If the previous dose level was well tolerated and no subjects experienced DLT at that level, an intermediate dose level may be defined by protocol amendment.

If the MTD is not established in the first 6 subjects administered 2 doses of MDX-010 at 3.0 mg/kg, additional subjects (6/group) will be randomized to receive either 4 doses of MDX-010 at 3.0 mg/kg or 2 doses of MDX-010 at 5.0 mg/kg. One or 2 of these cohorts that exhibit safety and evidence of efficacy will be expanded to 16 subjects.

In the 5 mg/kg cohort, should 2 DLTs occur, this will exceed the MTD and no additional subjects will be enrolled at this dose, but this will not automatically affect the enrollment in the 3 mg/kg cohort. If the 5 mg/kg cohort is expanded to 16 subjects, an occurrence of 6 or more DLTs in the 16 subjects will exceed the MTD.

In the 3 mg/kg cohort, DLTs that occur prior to the planned third dose will be considered together with the prior experience of the cohort that received 2 doses of 3 mg/kg, for a total of 12 subjects. If the total number of DLTs before 3 doses remains at 3 or less out of 12 subjects, dosing will continue, and subjects that were discontinued before the third dose will be replaced for this cohort. If 4 or more DLTs prior to the third dose occur in the group of 12, dosing of all subjects will be stopped, including the 5 mg/kg cohort, since the MTD for 2 monthly doses will have been exceeded.

DLTs that occur after the third dose of MDX-010 in the 3 mg/kg x 4 cohort will be considered separately, and 2 or more DLTs that occur after the third or fourth dose in the first 6 subjects to reach those doses will exceed the MTD for 4 doses of 3 mg/kg given monthly. The determination of exceeding the MTD with the third and fourth doses will not automatically affect dosing in the 5 mg/kg cohort If the 3 mg/kg x 4 doses cohort is expanded to 16 subjects, a total of 6 or more DLTs will exceed the MTD. In all cases, the DLTs will be reviewed by the sponsor and the investigator to assess whether to maintain, decrease, or stop dosing of other subjects under treatment.

6.3. Dose-Limiting Toxicity

A dose-limiting toxicity is defined as an adverse event or a new laboratory abnormality that occurs during or after the 4-week MDX-010 infusion period (up to 1 month post-treatment), judged to be possibly, likely, or definitely related to MDX-010 and meeting any of the following criteria:

- ≥Grade 3 toxicity that does not resolve (i.e., reduce to Grade 2 or less within 48 hours) with appropriate therapy (including steroids if necessary), excluding Grade 3 infusion reactions that resolve with appropriate therapy within 2 hours.

All adverse events that meet DLT criteria must be reported to Medarex, Inc. within 24 hours. All adverse events of ≥ Grade 3 or SAEs that are judged to be related to MDX-010 will be followed until resolution.

6.3.1. Management of Diarrhea

The occurrence of diarrhea, possibly due to inflammatory enteritis, was noted in 4 of 19 subjects in other ongoing melanoma trials where subjects receive either q3 or q4 week

infusions at 3 mg/kg in combination with a melanoma vaccine. The diarrhea was typically noted within 10 days of the second dose, therefore, in the current protocol, dose escalation will occur 10 days after all subjects in a dosing cohort have received their second dose. Any episodes of diarrhea should be evaluated to rule out any infectious or alternate etiology. Where possible, it is recommended to perform a sigmoidoscopy (or colonoscopy) with colonic biopsy with 3 to 5 specimens for standard paraffin block. Seven sections from each block will be sent to the immunopathology laboratory supporting the trial in addition to those sent to the local laboratory (instructions for handling may be found in Appendix 1). When appropriate therapy is inadequate, inflammatory diarrhea should be treated with steroids followed by a rapid taper.

6.3.2. Management of HIV Disease

The use of MDX-010 is intended to augment the level of anti-HIV immune responses that have been hindered due to excess expression of CD152. Increased activation of the immune system, due to either MDX-010 or tetanus immunization, has the potential for increasing the rate and level of HIV replication and adversely affecting CD4 counts. Previous experience with tetanus immunization in asymptomatic HIV-infected subjects has shown a transient elevation in HIV viremia that resolved spontaneously without adversely impacting CD4 counts.[27] Vaccinations are generally indicated for HIV infected subjects despite the transient increase in viral replication that may occur. Repeated vaccination with tetanus toxoid has also been shown to be safe in these subjects as a method of assessing augmented immune responses in chronically infected subjects.[4] Administration of single doses of MDX-010 to simian-HIV (SHIV)-infected pig-tailed macaques has not resulted in increases in viremia or reductions in CD4 levels (PD Greenberg, personal communication).

Subjects in this trial will have weekly assessment of viral load and CD4 counts. Following administration of MDX-010, an increase in viral load of ≥ 5 times above background (that is not associated with any treatment interruption) that is sustained over 3 consecutive weeks of monitoring will be considered a DLT and will prevent continued dosing of that subject. Reduction in CD4 counts of ≥ 50% that are sustained over 3 consecutive weeks of monitoring will be considered a DLT and prevent continued dosing of that subject.

6.4. Stopping Rules for Delayed Dose-Limiting Toxicity

The dose escalation schema will proceed based upon DLT experienced within the treatment and follow-up periods described above. DLTs experienced after 6 weeks that

are likely or definitely related to therapy with MDX-010 will be collected and evaluated by the Investigators and Medarex, Inc. on an ongoing basis.

Considering the expected half-life of the molecule, dose-related toxicity is most likely to occur during treatment or within the 4 weeks following treatment. Delayed toxicity will likely represent drug effect and not dose effect. Therefore, assessment of delayed toxicity will not directly impact on dose escalation, but will be assessed separately for dose limitation.

6.5. Infusion Reactions

Since MDX-010 contains only human protein sequences, it is unlikely that any allergic reaction will be seen in subjects. However, it is possible that infusion of MDX-010 will induce a cytokine release syndrome that could be evidenced by fever, chills, rigors, rash, pruritus, hypo- or hypertension, bronchospasm, or other symptoms. No prophylactic premedication will be given unless indicated by previous experience in an individual subject (detailed below). Reactions should be treated based upon the following recommendations:

- For mild symptoms: (Localized cutaneous reactions such as mild pruritus, flushing, rash.) Decrease the rate of infusion until recovery from symptoms, remain at bedside and monitor subject; complete MDX-010 infusion at the initial planned rate. Diphenhydramine 50 mg, may be administered at the discretion of the treating physician. Subjects may receive additional doses with close monitoring; premedication may be given at the discretion of the Investigator.
- For moderate symptoms: (Any symptom not listed above [mild symptoms] or below [severe symptoms] such as generalized pruritus, flushing, rash, dyspnea, hypotension with systolic BP >80 mmHg.) Interrupt MDX-010 infusion, administer diphenhydramine 50 mg i.v., remain at bedside and monitor subject until resolution of symptoms. Corticosteroids may abrogate any beneficial immunologic effect, but may be administered at the discretion of the treating physician. Resume MDX-010 infusion after recovery of symptoms. At the discretion of the treating physician, MDX-010 infusion may be resumed at one half the initial infusion rate, then increased incrementally to the initial infusion rate. If symptoms develop after resumption of the infusion, the infusion should be discontinued and no additional MDX-010 should be administered that day. The next dose of MDX-010 may be given with premedication (diphenhydramine and Tylenol) and careful monitoring, following the same treatment guidelines outline above. At the discretion of the treating physician additional oral or i.v. antihistamine may be administered.

- For severe symptoms: (Any reaction such as bronchospasm, generalized urticaria, systolic blood pressure <80 mm Hg, or angioedema.) Immediately discontinue infusion of MDX-010. Consider bronchodilators, epinephrine 1 mg i.v. or subcutaneously, and/or diphenhydramine 50 mg i.v. with solumedrol 100 mg i.v., as needed. Subjects should be monitored until the Investigator is comfortable that the symptoms will not recur. No further MDX-010 will be administered.

In case of late-occurring hypersensitivity symptoms (e.g., appearance within one week after treatment of a localized or generalized pruritus), symptomatic treatment may be given (e.g., oral antihistamine, or corticosteroids).

7. COMPLIANCE

The Investigator or designated study personnel are to maintain a log of all study drugs dispensed and returned. Drug supplies for each subject are to be inventoried and accounted for throughout the trial. All clinical supplies are to be stored in locked facilities until they are returned to Medarex, Inc. at the end of the study. All unused investigational products are to be returned to Medarex, Inc. at the closure of the study site or at an earlier time point if notified by Medarex, Inc.

8. CONCOMITANT THERAPY

All medications taken within 6 weeks prior to the administration of MDX-010, and all concomitant therapy administration during the study are to be recorded on the relevant Case Report Form (CRF) page(s), along with the reason for therapy use. All prior HIV-related antiviral and immunomodulator therapy are to be recorded on the relevant CRF page(s).

9. STUDY EVALUATIONS

9.1. Study Procedures by Visit

9.1.1. Overview

The study is divided into phases with associated evaluations and procedures that must be performed at specific time points, as described in the following sections. The Time and Events Schedules (Table 1) included in the Synopsis summarizes the frequency and timing of various pharmacokinetic, activity, safety, and other measurements.

As soon as the subject is considered for this study and prior to any other study procedures, the subject will have the nature of the study explained to them, and will be

asked to give written informed consent. Informed consent must be obtained prior to any procedures that do not form a part of the subject's normal care.

All subjects (withdrawn or completed) will have final evaluations and procedures performed (Study Completion/Withdrawal Visit).

9.1.2. Screening Phase

9.1.2.1. Prestudy (Visit 1)

Subjects will be evaluated for entry criteria during a screening period within 28 days prior to administration of study drug. The following procedures will be completed for each subject prior to inclusion in the study:

- Confirmation of HIV infection
- Demographics and medical history
- Current medication
- Vital sign measurements including weight, height, temperature, and resting systolic and diastolic blood pressure
- Complete physical examination
- ECG examination
- Chest radiograph
- Clinical laboratory tests:
  - Viral load assessment
  - CD4/CD8 counts
  - Quantitative tetanus enzyme immunoassay (EIA)
  - Screening serology (these labs can be substituted with documented previous labs within 3 months):
    - CRP
    - ANA
    - VDRL
    - SIL-2r
    - TNFα
    - STNFr
    - Neopterin
    - Rheumatoid factor
    - Hep B Ag and Ab (if positive, quantitative Hep B DNA PCR)
    - Hep C Ab (if positive, quantitative Hep C RT-PCR)
  - Hematology**:**
    - Hemoglobin
    - Hematocrit
    - CBC counts with differential (including absolute lymphocyte count) and direct platelet count.

- Clinical Chemistry**:**
    - Albumin
    - Serum alkaline phosphatase
    - SGOT (AST)
    - SGPT (ALT)
    - Bilirubin (direct and total)
    - Calcium
    - Creatinine
    - Glucose
    - Lactate dehydrogenase (LDH)
    - Total protein
    - Urea nitrogen (BUN)
    - Uric acid
    - Electrolytes (including sodium, potassium, chloride, and bicarbonate)
- Urinalysis:
  Gross examination including specific gravity, protein, glucose, and blood. Microscopic examination including WBC/high-powered field (HPF), RBC/HPF, and any additional finding.

- Collection of baseline signs and symptoms

9.1.2.2. Pre-entry (Visit 2)

Subjects who continue to meet inclusion/exclusion criteria after all initial screening labs will return for a pre-entry visit, 7 days prior to the first infusion. Measurements obtained during this visit will include the following:

- Vital sign measurements including weight, height, temperature, and resting systolic and diastolic blood pressure
- Serum β-HCG pregnancy test
- Screening serology:
  If ANA or rheumatoid factor were positive at prestudy Visit 1, the following screening serologies will have been reflexively tested and should be noted at this visit:
    - Anti-cardiolipin Ab;
    - TSH; and
    - Anti-thyroid antibody

  If hepatitis B or C were positive at prestudy Visit 1, quantitative viral load will have been reflexively tested and should be noted at this visit.
- Clinical Chemistry:
    - Albumin
    - Amylase
    - Serum alkaline phosphatase
    - SGOT (AST)
    - SGPT (ALT)
    - Bilirubin (direct and total)
    - Calcium
    - Creatinine
    - Glucose
    - Lactate dehydrogenase (LDH)
    - Lipase
    - Total protein
    - Urea nitrogen (BUN)
    - Uric acid
- Hematology (as outlined in Section 9.1.2.1.)
- Immunologic assessments:

Limited immunologic assessments:
- Population lymphocyte profiles (CD4, CD8 activation markers, memory, and naïve);
- Real-time LPA analyses for HIV, mitgens, tetanus (recall) and Candida (persistent) antigens by 7-day assay

Full immunologic assessments:
In addition to the limited immunologic assessments detailed above, full immunologic assessments will include the following:
- Cryopreserved lymphocytes;
- 48-hour cytokine induction by EIA;
- CD4 and CD8 lymphocyte responses to HIV, mitogens, Candida and tetanus antigens by cytokine induction (measurement of IFNγ, and IL-2 by IHC cytospin)

9.1.3. Treatment Phase for Subjects Receiving Two Doses – Infusion

The treatment period begins with the first i.v. infusion (Day 1, Visit 3) and continues through the end of Visit 14 (Day 57). Subjects who meet selection criteria will start MDX-010 treatment within 7 +/- 1 days of the pre-entry visit (Visit 2).

The subject will be given i.v. infusions of MDX-010 every 4 weeks for 2 doses. During this treatment phase, the following data will be collected and recorded in the CRF at each scheduled visit (or as indicated):

- MDX-010 infusion information (Visits 3 and 9)
- Vital sign measurements including weight, height, temperature, and resting systolic and diastolic blood pressure
- Targeted physical examination, including evaluation of heart, lungs, and abdomen (Visits 3 and 9)
- Tetanus booster (Visit 9 only; to be given at the end of the observation period following the second infusion of MDX-010)
- Urine pregnancy test (prior to dosing at Visits 3 and 9)
- Pharmacokinetic assessment (as outlined in Section 9.2; Visits 3, 4, 5, 6, 7, 9, 10, 11, 12, and 14)
- Serologic monitoring:
  - CRP, sIL-2r, TNFα, sTNFr, and neopterin will be monitored at Visits 9 and 14
  - ANA and rheumatoid factor will be tested in all subjects at Visits 9 and 14
  - If ANA, rheumatoid factor, anti-cardiolipin Ab, TSH, or anti-thyroid Ab were positive during the screening phase or become positive, then all of these parameters will be monitored at Visits 9 and 14
  - If hepatitis B or C viral loads were detectable during the screening phase, these parameters will be monitored at Visits 9 and 14
- Clinical laboratory tests:
  - Viral load assessments
  - CD4/CD8 counts

- Immunologic assessments (as outlined in Section 9.1.2.2.)
    - Limited immunologic assessments will be performed at Visits 3, 4, 6, 9, 11, and 13
    - Full immunology assessments will be performed at Visits 8 and 14
- Quantitative tetanus EIA (Visits 3, 8, 9, 10, 11, 12, 13, and 14)
- Hematology* (as outlined in Section 9.1.2.1)
- Clinical Chemistry* (Visits 3, 6, 9, and 14; as outlined in Section 9.1.2.2)
- Urinalysis (Visits 3, 9, and 14; as outlined in Section 9.1.2.1)

* Local CBC and chemistry panel: results to be reviewed and found acceptable prior to the second infusion of MDX-010 (Visit 8)

- HAHA plasma sample (prior to dosing at Visits 3 and 9)
- Concomitant medications
- Adverse event assessment

9.1.4. Treatment Phase for Subjects Receiving Four Doses – Infusion

The treatment period begins with the first i.v. infusion (Day 1, Visit 3) and continues through the end of Visit 28 (Day 141). Subjects who meet selection criteria will start MDX-010 treatment within 7 +/- 1 days of the pre-entry visit (Visit 2).

The subject will be given i.v. infusions of MDX-010 every 4 weeks for 4 doses. During this treatment phase, the following data will be collected and recorded in the CRF at each scheduled visit (or as indicated):

- MDX-010 infusion information (Visits 3, 9, 15, and 19)
- Vital sign measurements including weight, temperature, and resting systolic and diastolic blood pressure
- Targeted physical examination, including evaluation of heart, lungs, and abdomen (Visits 3, 9, 15, and 19)
- Tetanus booster (Visit 9 only; to be given at the end of the observation period following the second infusion of MDX-010)
- Urine pregnancy test (prior to dosing at Visits 3, 9, 15, and 19)
- Pharmacokinetic assessment (as outlined in Section 9.2; Visits 3 through 7, 9 through 12, and 14 through 23)
- HAHA plasma sample (prior to dosing at Visits 3, 9, 15, and 19)
- Serologic monitoring:
    - CRP, sIL-2r, TNFα, sTNFr, and neopterin will be monitored at Visits 9, 15, and 19
    - ANA and rheumatoid factor will be tested in all subjects at Visits 9, 15, and 19
    - If ANA, rheumatoid factor, anti-cardiolipin Ab, TSH, or anti-thyroid Ab were positive during the screening phase or become positive, then all of these parameters will be monitored at Visits 9, 15, and 19
    - If hepatitis B or C viral loads were detectable during the screening phase, these parameters will be monitored at Visits 9, 15, and 19

- Clinical laboratory tests:
  - Viral load assessments
  - CD4/CD8 counts
  - Immunologic assessments (as outlined in Section 9.1.2.2.)
    - Limited immunologic assessments will be performed at Visits 3, 4, 6, 9, 11, 13, 14, and 18
    - Full immunology assessments will be performed at Visits 8 16, 20, and 23
  - Quantitative tetanus EIA (Visits 3, 8 through 14, 16, 18, 20, and 23)
  - Hematology* (as outlined in Section 9.1.2.1)
  - Clinical Chemistry* (Visits 3, 6, 8, 9, 14, 15, 18, 19, and 23; as outlined in Section 9.1.2.2)
  - Urinalysis (Visits 3, 9, and 14; as outlined in Section 9.1.2.1)

  * Local CBC and chemistry panel: results to be reviewed and found acceptable prior additional infusions of MDX-010 (Visits 8, 14, and 18)
- Concomitant medications
- Adverse event assessment

9.1.5. Follow-up Phase

Subjects who complete all doses of MDX-010 or who discontinue prematurely for reasons other than death, withdrawal of consent, or are lost to follow-up will return for 2 follow-up evaluations biweekly (Visits 15 and 16 for subjects receiving 2 doses, Visits 24 and 25 for subjects receiving 4 doses). At follow-up visits, subjects will undergo the following:

- Vital sign measurements including weight, height, temperature, and resting systolic and diastolic blood pressure
- Complete physical examination including evaluation of heart, lungs, and abdomen (Visits 16 or 25 only)
- Serologic monitoring:
  - CRP, sIL-2r, TNFα, sTNFr, and neopterin will be monitored at Visits 16 or 25 (Termination)
  - ANA and rheumatoid factor will be tested in all subjects at Visit 16 or 25 (Termination)
  - If ANA, rheumatoid factor, anti-cardiolipin Ab, TSH, or anti-thyroid Ab were positive during the screening phase or become positive, then all of these parameters will be monitored at Visits 16 or 25 (Termination).
  - If hepatitis B or C viral loads were detectable during the screening phase, these parameters will be monitored at Visit 16 or 25 (Termination).
- Quantitative tetanus EIA (Visits 16 or 25 only)
- Hematology (as outlined in Section 9.1.2.1)
- Clinical chemistry (Visits 16 or 25 only; as outlined in Section 9.1.2.1) amylase and lipase to be measured at Visits 15, 21, and 24
- Urinalysis (Visits 16 or 25 only)

- Viral load
- CD4/CD8 counts
- Pharmacokinetic assessment (Visits 16 or 25 only)
- HAHA plasma sample (Visits 16 or 25 only)
- Limited immunologic assessment (Visits 16 or 25 only; as outlined in Section 9.1.2.2)
- Concomitant medications
- Adverse event assessment

9.1.6. Termination Visit (Visits 16 or 25)

A Study Completion/Early Withdrawal visit (Visit 16 for subjects receiving 2 doses, Visit 25 for subjects receiving 4 doses) will be scheduled for all subjects enrolled in the study. If subjects prematurely withdraw, these procedures will be completed on the day of withdrawal from the study.

If subjects are withdrawn prior to completing 85 or 141 days of the study, the reason for withdrawal will be documented in the CRF and in the source document. All available subjects (withdrawn or completed) will have the following evaluations and procedures completed:

- Vital sign measurements including weight, height, temperature, and resting systolic and diastolic blood pressure
- Full physical examination
- Hematology
- Clinical chemistry
- Urinalysis
- Viral load
- CD4/CD8 counts
- Pharmacokinetic assessment
- HAHA plasma sample
- Limited immunologic assessment
- Serologic monitoring:
    - CRP, sIL-2r, TNFα, sTNFr, and neopterin will be monitored at termination
    - If ANA, rheumatoid factor, anti-cardiolipin Ab, TSH, or anti-thyroid Ab were positive during the screening phase, these parameters will be monitored at termination
    - If hepatitis B or C viral loads were detectable during the screening phase, these parameters will be monitored at termination
- Quantitative tetanus EIA
- Concomitant medications
- Adverse event assessment

9.2. Pharmacokinetic Evaluations

Blood samples for the analysis of plasma concentrations of MDX-010 will be drawn according to the following schedule:

9.2.1. For Subjects Receiving Two Doses

Treatment Phase:

Visits 3 and 9: 30 minutes prior to infusion, 120 minutes and 4 hours post infusion start time 0.

Visits 4, 5, 6, 7, 10, 11, 12, and 14: 1 sample will be obtained.

Follow-up Phase:

Visit 16: 1 sample will be obtained.

9.2.2. For Subjects Receiving Four Doses

Treatment Phase:

Visits 3, 9, 15, and 19: 30 minutes prior to infusion, 120 minutes and 4 hours post infusion start time 0.

Visits 4, 5, 6, 7, 10, 11, 12, 14, 16, 17, 18, 20, 21, 22, and 23: 1 sample will be obtained.

Follow-up Phase:

Visit 25: 1 sample will be obtained.

Instructions for the handling, storage, and shipment of pharmacokinetic blood samples are provided in Appendix 1.

9.3. Clinical Activity Evaluations

The primary surrogate clinical activity parameter is viral load assessment. A decrease in viral load of at least 0.5 log sustained over 2 or more consecutive determinations will indicate potential clinical activity.

The secondary activity parameter is CD4 count. A CD4 count that increases by more than 20% of entry level sustained over the course of 2 or more consecutive determinations during the trial will indicate potential clinical activity.

9.4. Immunologic Activity Evaluations

Evaluation of immunologic activity will include the following:

- Changes in frequency and amount of CD4 and CD8 cells with cytokine responses to HIV, Candida, and tetanus antigens (> 3-5x increase in total number of responding cells).
- Changes in LPA to HIV, Candida, and tetanus antigens. (> 3-5x increase in stimulation index).
- Changes in quantitative anti-tetanus antibody titer (greater than 4 fold increase in titer sustained over 2 weeks post-vaccination).

9.5. Safety Evaluations

The following evaluations will be performed during the study to measure the safety and tolerability of MDX-010:

- Vital sign measurements including weight, height, temperature, and resting systolic and diastolic blood pressure
- Physical examination
- Plasma sample for HAHA
- Clinical laboratory tests (as outlined in Section 9.1.2)
- Adverse event assessment

10. ADVERSE EVENT REPORTING

10.1. Definitions

An adverse event is any undesirable sign, symptom, clinically significant laboratory abnormality, or medical condition occurring after starting study treatment, even if the event is not considered to be treatment-related. Each adverse event is to be reported on an Adverse Event CRF page. Adverse events are graded primarily using the NIH/NIAID DIVISION OF AIDS (DAIDS) TABLE for GRADING SEVERITY of ADULT ADVERSE EXPERIENCES August, 1992 (available on the internet at http://aactg:cure@roc.s-3.com/members/download/adulttox.pdf). Adverse events not covered in these guidelines will then be covered by the Cancer Therapy Evaluation Program Common Toxicity Criteria (CTC), Version 2.0, DCTD, NCI, NIH, DHHS, April 30, 1999 (located on the internet at http://ctep.cancer.gov/forms/CTCv20_4-30-992.pdf). If DAIDS or CTC grading does not exist for an adverse event, the intensity of mild (1), moderate (2), severe (3), and life-threatening (4) will be used. Information about all adverse events, whether volunteered by the subject, discovered by Investigator questioning, or detected through physical examination, laboratory testing, or other means, will be collected and recorded on the Adverse Event CRF page and followed as appropriate. Adverse event monitoring should be continued for at least 30 days following

the last dose of investigational product, until adverse event resolution/stabilization, or through all follow-up periods, if applicable.

Medical conditions/diseases present before starting study treatment are only considered adverse events if they worsen after starting study treatment. Clinical events occurring before starting study treatment but after signing the informed consent form are recorded on the Medical History/Current Medical Conditions CRF page. Abnormal laboratory values or test results constitute adverse events only if they induce clinical signs or symptoms or require therapy, in which case they should be recorded on the Adverse Events CRF page and should include the signs, symptoms or diagnosis associated with them.

As far as possible, each adverse event will also be described by:

1 Description
1. Duration (start and end dates)
2. DAIDS Grade 1-4 or intensity if DAIDS or CTC not available
3. Relationship to the investigational product attribution (by categories below)
4. Action(s) taken to treat the adverse event
5. Outcome

The relationship of each adverse event to study drug will be defined as “unrelated”, “unlikely”, “possible”, “probable”, or “definite”. The Investigator is responsible for determining the study drug relationship for each adverse event that occurs during the study. Assessments are to be recorded on the CRF.

1. Unrelated it is beyond all reasonable doubt that the reported AE was caused by the study drug.
2. Unlikely there is little possibility that the study drug caused the reported AE; and another factor(s) including concurrent illnesses, progression and expression of the disease state, concurrent medications, or a reaction to concurrent medications appear to explain the AE.
3. Possible the reported AE follows a reasonable temporal sequence from administration of the study drug, but could reasonably be explained by the subject’s clinical state or concurrent therapy.
1. Probable the reported AE follows a reasonable temporal sequence from administration of the study drug, and could <u>not</u> reasonably be explained by the subject’s clinical state or concurrent therapy.
5. Definite the reported AE follows an anticipated response and a reasonable temporal sequence to the study drug; and is confirmed by both improvement upon stopping of study drug (withdrawal) and reappearance of the reaction on repeated exposure (rechallenge).

The actions taken in response to an adverse event are described on a numerical scale (from 0 to 5) that cover the various possibilities. One or more of these are to be selected:

0 No action taken
1 Investigational product dosage adjusted/temporarily interrupted
2 Investigational product permanently discontinued due to this adverse event
3 Concomitant medication taken
4 Non-drug therapy given
5 Hospitalization/prolonged hospitalization

10.2. Serious Adverse Events

Information about all serious adverse events will be collected and recorded on the Serious Adverse Event Report Form. To ensure subject safety, each serious adverse event must also be reported to Medarex, Inc. within 24 hours of learning of its occurrence. A serious adverse event is defined in general as an untoward (unfavorable) event which:

1. is fatal or life-threatening;
1. requires or prolongs hospitalization;
2. is significantly or permanently disabling or incapacitating;
3. constitutes a congenital anomaly or a birth defect; or
4. may jeopardize the subject and may require medical or surgical intervention to prevent one of the outcomes listed above.

Hospitalizations occurring under the following circumstances are not considered serious adverse events: admission to a hospice for respite care; hospitalizations planned before entry into the clinical study; hospitalization for elective treatment of a condition unrelated to the studied indication or its treatment; hospitalization on an emergency, outsubject basis that does not result in admission (unless fulfilling the criteria above); hospitalization as part of the normal treatment or monitoring of the studied indication; and hospitalization not associated with any deterioration in condition.

10.3. Instructions for Rapid Notification of Serious Adverse Events

10.3.1. Reporting Responsibility

Any serious adverse event occurring in a subject after providing informed consent, while receiving study treatment, or in the 4 weeks following study treatment or follow-up must be reported. The period after discontinuing investigational product may be extended if there is a strong suspicion that the drug has not yet been eliminated. All serious adverse events must also be reported for the period in which the study protocol interferes with the standard medical treatment given to a subject.

Each serious adverse event must be reported by the Investigator to Medarex, Inc. within 24 hours of learning of its occurrence, even if it is not felt be treatment-related. Follow-up information about a previously reported serious adverse event must also be reported to Medarex, Inc. within 24 hours of receiving it. If the serious adverse event has not been previously documented (unexpected) and it is thought to be related to investigational product, Medarex, Inc. may contact the Investigator to obtain further information. If warranted, an Investigator alert may be issued, to inform all Investigators involved in any study with the same drug that this serious adverse event has been reported.

10.3.2. Reporting Procedures

The Investigator must complete the Serious Adverse Event Report Form in English, assess the causal relationship to study treatment and send the completed form by fax within 24 hours to Medarex, Inc. The original and the duplicate copies of the Serious Adverse Event Form, and the fax confirmation sheet must be kept with the site's study records. The monitor will collect a copy of the Serious Adverse Event Form and deliver it to Medarex, Inc.

Follow-up information should be sent to the same person at Medarex, Inc. who was sent the original Serious Adverse Event Form. Either a new Serious Adverse Event Form is sent (stating that this is a follow-up), or the original one resubmitted (with the new information highlighted and a new date provided). The follow-up report should describe whether the event has resolved or continues, if and how it was treated, and whether the subject continued or discontinued study participation. The form and fax confirmation sheet must be retained.

10.3.3. Contact Persons and Numbers

The telephone and telefax numbers of the Medarex, Inc. contact person are listed in the Investigator File provided at the site.

11. SUBJECT COMPLETION/WITHDRAWAL

11.1. Completion

It will be documented whether or not each subject completed the Treatment Phase of the clinical study (to the end of Visit 10 or Visit 23), how long they were followed, and the reason for withdrawal.

11.2. Discontinuation of Treatment

If for any reason, either study treatment or observations were discontinued, the reason will be recorded. Reasons for discontinuation include one of the following:

- Adverse event(s)
- Protocol violation
- Disease progression (deterioration in HIV-related disease)
- Subject withdrew consent
- Lost to follow-up
- Death
- Other

Subjects who complete all requirements or who prematurely discontinue from the study should have the study completion/discontinuation evaluations performed on the day of study completion/discontinuation. Subjects who discontinue due to a study-related adverse event or abnormal laboratory value must be followed at least once a week for 4 weeks (and subsequently at 4-week intervals) until resolution or stabilization of the event. If a subject has not recovered from any toxicity within 2 weeks of prematurely discontinuing study drug, s/he must be discontinued from the study, but will continue to be followed for toxicity as described above. All subjects will be followed for adverse events for 30 days following completion of premature discontinuation from the study, or until resolution/stabilization of the adverse event.

Subjects who have not met the minimum requirements defined in Section 12.2 will be regarded as ineligible for the evaluable population and must be replaced.

Subjects will be discontinued from the study for drug-related toxicities as outlined in Section 6.

12. STATISTICAL METHODS

12.1. Sample Size Determination

The sample size of up to 50 patients is based on the trial design for dose escalation and safety evaluation requirements, with up to 30 patients (3 to 6 patients/cohort) at 5 dose levels or treatment regimens and additional 20 patients in the selected 2 cohorts for the evaluation of clinical activity. The primary clinical activity parameter is the proportion of patients who achieve clinical response (a decrease in viral load of at least 0.5 log sustained over two consecutive viral load assessments). It is assumed that none of the patients would have clinical response if they would not have received any therapy and the response rate (RR) for patients in the treatment of 3 mg/kg or 5 mg/kg MDX-010

would be 10%. A one-sample exact Binomial test will be used in testing the alternative hypothesis H_a: $RR_{\text{with treatment}}$ 10% against the null hypothesis H_0: $RR_{\text{without treatment}}$ 0%. A sample size of 16 evaluable patients is required to provide more than 80% power in a two-sided exact Binomial test at the significance level of 0.05.

12.2. Study Population

The following subject data sets will be defined:

12.2.1. Safety Population

Includes all subjects in the study who received at least 1 dose or any partial dose of MDX-010.

12.2.2. Clinical Activity Population

Includes all subjects in the study who completed at least 1 dose of MDX-010 and 4 weeks of follow up assessments.

12.2.3. Immunologic Activity Population

The immunologic activity population is the same as the clinical activity population.

An evaluable subject for tetanus response will have completed 2 doses of MDX-010, receive the tetanus booster, and have 4 weeks of follow up assessments after the second infusion.

12.3. Statistical Consideration

Unless otherwise indicated, statistical significance will be declared if the two-sided p value is ≤ 0.05.

12.4. Statistical Analysis

The data collected in this study will be analyzed by the Biostatistics group at Medarex, Inc. or its designees.

All data will be listed individually by subject. For quantitative parameters, descriptive statistics will include the mean, standard deviation, minimum, median, and maximum. For qualitative parameters, descriptive statistics will include the frequency and percentage.

12.4.1. Demographics and Baseline Characteristics

Subject demographics and baseline characteristics including age, sex, race, height, weight, viral load assessment, immunologic assessment, medical conditions, etc. will be summarized by cohort using descriptive statistics.

12.4.2. Extent of Exposure

The actual dose of MDX-010 will be summarized by cohort using descriptive statistics.

12.4.3. Dose Escalation

The probability that dose escalation (PrDE) will be permitted at any stage during MTD determination is a function of the underlying DLT rate (p) at the current dose level. This probability can be calculated as the sum of the binomial probabilities of the following 2 possible outcomes that would permit escalation to occur: (1) no DLT is observed in the first 3 MTD evaluable patients; or (2) one DLT is observed in the first 3 MTD evaluable patients, followed by no DLT observed in 3 additional patients at the same dose level, as expressed by the following formula:

$$Pr_{DE} \quad q^3 + (3pq^2)q^3 \quad q^3 + 3pq^5, \text{ where } q \quad 1 - p.$$

The following table provides the probabilities of dose escalation (PrDE) for a range of underlying DLT rates (p):

Probabilities of Dose Escalation

Underlying DLT Rate (%) P	Probability of Dose Escalation (%) Pr_{DE}
10%	91%
20%	71%
30%	49%
40%	31%
50%	17%
60%	8%
70%	3%
80%	1%
90%	0.1%

Thus, the probability of dose escalation is no more than 3% when the underlying DLT rate is ≥70%, and when the DLT rate is ≤ 10%, the likelihood of escalation is more than 91%.

Those patients who experience at least one DLT will be listed individually by cohort and reason for DLT.

12.4.4. Concomitant Medication

Concomitant medications and significant non-drug therapies will be summarized by cohort using descriptive statistics.

12.4.5. Activity

Primary Activity Parameter

The primary clinical activity parameter is the proportion of patients who achieve clinical response in the viral load assessment. A decrease in viral load of at least 0.5 log sustained over two consecutive assessments will indicate potential clinical activity. Viral load will be summarized by cohort using descriptive statistics. A one-sample exact Binomial test will be used in testing the alternative hypothesis Ha: $RR_{with\ treatment}$ 10% against the null hypothesis H0: $RR_{without\ treatment}$ 0%. in the selected 2 cohorts. An exact 95% confidence interval will also be provided.

Secondary Activity Parameters

The secondary activity parameter is CD4 cell counts. A CD4 count that increases by more than 20% of baseline over 2 consecutive assessments will indicate potential clinical activity. CD4 cell counts will be summarized by cohort using descriptive statistics.

Immunologic Activity Parameter

Evaluation of immunologic efficacy will include assessments of changes in frequency and amount of CD4 and CD8 cells with cytokine responses to HIV, Candida, and tetanus antigens; changes in LPA to HIV, Candida, and tetanus antigens; and changes in anti-tetanus antibody levels. All immunologic activity parameters will be summarized by cohort using descriptive statistics.

12.4.6. Safety

The safety analysis will be conducted on the safety population. The following safety parameters will be evaluated:

Adverse Events

All adverse events recorded during the study will be summarized using descriptive statistics. The incidence of treatment-emergent adverse events (new or worsening from baseline) will be summarized by body system, type of adverse event, intensity, and relationship to the investigational product.

Vital Signs

The change from baseline to each notable post-baseline visit will be summarized by cohort using descriptive statistics.

Clinical Laboratory Tests

Clinical laboratory test values outside the normal range and clinically significant abnormal range will be flagged in the data listing.

Laboratory data will be summarized by cohort using shift tables (baseline to notable shift post-baseline value). The change from baseline will be summarized by cohort using descriptive statistics.

Physical Examinations

The abnormal findings in physical examinations will be summarized by cohort using descriptive statistics.

Diagnostic Tests

Diagnostic tests (including ECG, chest radiograph, pregnancy test, and screening serologies) will be summarized appropriately.

Special Immune Function Measurements

Special immune function measurements will be summarized appropriately.

12.4.7. Pharmacokinetic Parameters

Plasma concentrations of MDX-010 will be determined by a validated method at the times listed in Tables 1 and 2.

Definition and Calculation of PK Parameters

The following pharmacokinetic parameters will be estimated and reported:

$AUC_{0\ t}$	Area under the concentration-time curve from the time of dosing to the time of the last observation (calculated by linear trapezoidal summation).
$AUC_{0\ \infty}$	Area under the curve from the time of dosing extrapolated to infinity (calculated by linear trapezoidal summation and extrapolated to infinity).
C_{max}	Maximum plasma concentration observed post-dose.
t_{max}	Time at which the C_{max} occurs.
$t_{1/2}$	Elimination half-life; determined as $0.693/\lambda_z$.

All pharmacokinetic parameters will be summarized by cohort using descriptive statistics. Individual as well as mean concentration-time plots will be depicted for plasma concentrations.

12.5. Statistical Software

All statistical analyses will be performed using SAS® version 8.2 or higher.

13. STUDY DRUG INFORMATION

13.1. MDX-010

MDX-010 will be supplied at a concentration of 5 mg/mL in vials containing 5 or 10 mL. For administration, MDX-010 is to be filtered through a sterile, low-protein binding, 0.22 µm filter (provided by Medarex, Inc.). The total dose needed should be diluted to a concentration of no less than 0.2 mg/mL in 0.9% sodium chloride.

MDX-010 will be administered to the first cohort at a dosage of 0.1 mg/kg, administered as an i.v. infusion at a rate of 1 mL/min (controlled by a volumetric pump) using a 1.2 µm in-line filter for up to 90 minutes, with a 10 cc flush at the end. Subsequent cohorts will be administered MDX-010 at dosages of 1, 3, and 5 mg/kg.

Total dose should be calculated as follows:

Subject body weight in kg x 3 mg (for the 3 mg/kg cohort)   total dose, mg

For example, a subject with a body weight of 70 kg would be administered 210 mg of MDX-010 (70 kg x 3.0 mg/kg   210 mg). Dose adjustment is not allowed.

If ≥Grade 3 bronchospasm or other hypersensitivity reaction occurs, the infusion should be discontinued and no additional MDX-010 should be administered.

Reactions felt to be attributable to MDX-010 include sweating, arthralgia, fatigue, headache, nausea, rash, pruritis, tumor pain and retinal depigmentation. Some mild infusion reactions including fever, chills and hypotension have been seen.

13.2. Storage Conditions and Handling of MDX-010

MDX-010 must be stored at a temperature ≥ 2° C and ≤ 8°C.

Stability studies indicate that following dilution in 0.9% saline to concentrations of 0.2 and 3.0 mg/mL, the solution can be stored in i.v. bags for 24 Hours at 27 to 33°C or refrigerated at 2 to 8°C without loss of protein concentration or product stability.

14. ETHICAL ASPECTS

14.1. Ethics and Good Clinical Practice

This study must be carried out in compliance with the protocol and in accordance with Medarex, Inc. standard operating procedures. These are designed to ensure adherence to Good Clinical Practice, as described in the following documents:

1. ICH Harmonized Tripartite Guidelines for Good Clinical Practice 1996.
1. Directive 91/507/EEC, The Rules Governing Medicinal Products in the European Community.
2. Declaration of Helsinki, concerning medical research in humans (Recommendations Guiding Physicians in Biomedical Research Involving Human Subjects, Helsinki 1964, amended Tokyo 1975, Venice 1983, Hong Kong 1989, Somerset West 1996, Edinburgh 2000).

The Investigator agrees, when signing the protocol, to adhere to the instructions and procedures described in it and thereby to adhere to the principles of Good Clinical Practice that it conforms to. A copy of the Declaration of Helsinki may be located on the internet at: http://www.wma.net/e/policy/17-c_e.html.

14.2. Institutional Review Board/Independent Ethics Committee

Before implementing this study, the protocol, the proposed informed consent form, and other information to subjects must be reviewed by an Institutional Review Board/Independent Ethics Committee (IRB/IEC). A signed and dated statement that the protocol and informed consent have been approved by the IRB/IEC must be given to Medarex, Inc. before study initiation. The name and occupation of the chairperson and the members of the IRB/IEC (preferred) or the IRB's HHS Assurance number must be supplied to Medarex, Inc. Any amendments to the protocol which need formal approval as required by local law, will be approved by this committee. The IRB may be notified for all other amendments (i.e. administrative changes)

14.3. Informed Consent

The Investigator will explain to each subject (or legally authorized representative) the nature of the research study, its purpose, the procedures involved, the expected duration, alternative treatment, the potential risks and benefits involved and any discomfort which may occur. Each subject will be informed that participation in the study is voluntary and that he/she may withdraw from the study at any time and that withdrawal of consent will not affect his/her subsequent medical treatment or relationship with the treating physician.

This informed consent should be given by means of a standard written statement, written in non-technical language. The subject should read and consider the statement before signing and dating it, and should be given a copy of the signed document. If written consent is not possible, oral consent can be obtained if witnessed by a signed statement from one or more persons not involved in the study, mentioning why the subject was unable to sign the form. For those subjects who are not of adult age, written parental permission must be obtained. No subject can enter the study before his/her informed consent has been obtained.

The informed consent form is part of the protocol, and must be submitted by the Investigator with it for IRB/IEC approval. Medarex, Inc. supplies a proposed informed consent form, which complies to regulatory requirements and is considered appropriate for the study. Any changes to the proposed consent form suggested by the Investigator must be agreed to by Medarex, Inc. before submission to the IRB/IEC, and a copy of the approved version must be provided to the Medarex, Inc. monitor after IRB/IEC approval.

15. ADMINISTRATIVE REQUIREMENTS

15.1. Protocol Amendments

Any change or addition to this protocol requires a written protocol amendment that must be approved by the sponsor, Medarex, Inc., before implementation. Amendments significantly affecting the safety of subjects, the scope of the investigation or the scientific quality of the study, require additional approval by the IRB/IEC of all centers, and, in some countries, by the regulatory authority. A copy of the written approval of the IRB/IEC must be given to the Medarex, Inc. monitor or their designee. Examples of amendments requiring such approval are:

1. Increase in drug dosage or duration of exposure of subjects;
1. Significant change in the study design (e.g. addition or deletion of a control group);
2. Increase in the number of invasive procedures to which subjects are exposed; or
3. Addition or deletion of a test procedure for safety monitoring.

These requirements for approval should in no way prevent any immediate action from being taken by the Investigator or by Medarex, Inc. in the interests of preserving the safety of all subjects included in the trial. If an immediate change to the protocol is felt to be necessary by the Investigator and is implemented by him/her for safety reasons Medarex, Inc. should be notified and the IRB/IEC at the center should be informed within 10 working days.

Amendments affecting only administrative aspects of the study do not require formal protocol amendments or IRB/IEC approval but the IRB/IEC of each center must be kept informed of such administrative changes. Examples of administrative changes not requiring formal protocol amendments and IRB/IEC approval that can be treated as administrative amendments include but are not limited to:

1. Changes in the staff used to monitor trials (e.g. Medarex, Inc. staff versus a contract research organization [CRO]) and
1. Minor changes in the packaging or labeling of investigational product.

15.2. Monitoring Procedures

Before study initiation, at a site initiation visit or at an Investigator's meeting, a Medarex, Inc. representative will review the protocol and CRF with the Investigators and their staff. During the study, the Medarex, Inc. monitor or its designee will visit the site regularly to check the completeness of subject records, the accuracy of entries on the CRFs, the adherence to the protocol and to Good Clinical Practice, the progress of enrollment, and also to ensure that investigational product is being stored, dispensed and accounted for according to specifications.

The Investigator must give the monitor access to relevant hospital or clinical records to confirm their consistency with the CRF entries. No information in these records about the identity of the subjects will leave the study center. Medarex, Inc. monitoring standards require full verification for the presence of informed consent, adherence to the inclusion/exclusion criteria, documentation of serious adverse events and the recording of primary activity and safety variables. Additional checks of the consistency of the source data with the CRFs are performed according to the study-specific monitoring plan.

15.3. Recording of Data and Retention of Documents

Data on subjects collected on CRFs during the trial will be documented in an anonymous fashion and the subject will only be identified by the subject number, and by his/her initials if also required. If, as an exception, it is necessary for safety or regulatory reasons to identify the subject, both Medarex, Inc. and the Investigator are bound to keep this information confidential.

All the information required by the protocol should be provided; any omissions require explanation. All CRFs should be completed and available for collection within a timely manner, preferably no more than 10 days after the subject's visit (except for the last visit of the last subject, which should be completed in a timely manner, preferably within 5 working days), so that the monitor may check the entries for completeness, accuracy

and legibility, ensure the CRF is signed by the Investigator and transmit the data to Medarex, Inc.

All entries to the CRFs must be made clearly in black ball-point pen, to ensure the legibility of self-copying or photocopied pages. Corrections will be made by placing a single horizontal line through the incorrect entry, so that it can still be seen, and placing the revised entry beside it. The revised entry must be initialed and dated by a member of the Investigator's research team authorized to make CRF entries. Correction fluid must not be used.

The Investigator must maintain source documents for each subject in the study. All information on CRFs will be traceable to these source documents, which are generally maintained in the subject's file. The source documents will contain all demographic and medical information, including laboratory data, electrocardiograms, etc., also a copy of the signed informed consent form, which should indicate the study number and title of the trial.

Essential documents, as listed below, will be retained by the Investigator for as long as needed to comply with national and international regulations. Medarex, Inc. will notify the Investigator(s)/institution(s) when the study-related records are no longer required. The Investigator agrees to adhere to the document retention procedures by signing the protocol. Essential documents include:

1. Signed protocol and all amendments;
2. IRB/IEC approvals for the study protocol and all amendments;
3. All source documents and laboratory records;
4. CRF copies;
5. Subjects' Informed Consent Forms; and
6. Any other pertinent study document.

15.4. Auditing Procedures

In addition to the routine monitoring procedures, Medarex, Inc. or its designees may conduct audits of clinical research activities in accordance with internal Standard Operating Procedures (SOPs) to evaluate compliance with the principles of Good Clinical Practices. Medarex, Inc., its designee, or a regulatory authority may wish to conduct an inspection (during the study or after its completion). If an inspection is requested by a regulatory authority, the Investigator will inform Medarex, Inc. immediately that this request has been made.

15.5. Handling and Accountability of Investigational Product

Medarex, Inc. is the manufacturer and provider of the investigational product supply. All investigational product will be supplied to the Principal Investigator by Medarex, Inc. Investigational product supplies must be kept in an appropriate, secure area (e.g. locked cabinet) and stored in accordance with the conditions specified on the labels. An accurate record of the date and amount of Investigational product dispensed to each subject must be available for inspection at any time.

All investigational products are to be used only for this protocol and not for any other purpose. The Investigator must not destroy any investigational product labels, or any partly-used or unused investigational product supply, unless specifically instructed by the Sponsor. At the conclusion of the study, and, as appropriate during the course of the study, the Investigator will return all unused investigational product containers, investigational product labels and a copy of the completed investigational product disposition form to the Medarex, Inc. monitor or its designee, or to the Medarex, Inc. address provided in the Investigator folder provided for each site.

Ordering study medication: Clinical supplies may be requested by completing a Request Form and faxing it to the Study Manager at Medarex, Inc. The contact information for Medarex, Inc. personnel will be filed in an Investigator's File.

Inventory: Upon receipt, an inventory will be conducted to confirm that the clinical supplies are sufficient for study requirements, and that they correspond to the invoices enclosed in the shipping package. A Receipt of Shipment Confirmation form will be faxed to the Study Manager or appropriate drug delivery personnel. A separate Accountability Log will be used to record all medication infused to trial subjects.

Study medication preparation: Treatment instructions and study medication for dispensing will be prepared as specified in Section 13.

Dispensing supplies: Clinical supplies for the Treatment Phase will be administered to the subject from open-label stock. The Investigator will assume responsibility for administration. The Investigator must maintain an accurate record of the dispensing of the investigational product in an Investigational Product Accountability Log, a copy of which must be given to Medarex, Inc. at the end of the study. The accountability ledger will record the investigational product used, dosages and the time administered. The accountability will be reviewed and noted by the field monitor during site visits and at the completion of the trial.

15.6. Publication of Results

Any formal presentation or publication of data collected from this trial will be considered as a joint publication by the Investigator(s) and the appropriate personnel of Medarex, Inc. Authorship will be determined by mutual agreement. For multicenter studies, it is mandatory that the first publication is based on data from all centers, analyzed as stipulated in the protocol by Medarex, Inc. statisticians, and not by the Investigators themselves. Investigators participating in multicenter studies agree not to present data gathered from one center or a small group of centers before the full, initial publication, unless formally agreed to by all other Investigators and Medarex, Inc.

Medarex, Inc. must receive copies of any intended communication in advance of publication (at least 15 working days for an abstract or oral presentation and 45 working days for a journal submission). Medarex, Inc. will review the communications for accuracy (thus avoiding potential discrepancies with submissions to health authorities), verify that confidential information is not being inadvertently divulged and to provide any relevant supplementary information. Authorship of communications arising from pooled data may include members from each of the contributing centers as well as Medarex, Inc. personnel.

15.7. Disclosure and Confidentiality

By signing the protocol, the Investigator agrees to keep all information provided by Medarex, Inc. in strict confidence and to request similar confidentiality from his/her staff and the IRB/IEC. Study documents provided by Medarex, Inc. (protocols, Investigators' brochures, CRFs and other material) will be stored appropriately to ensure their confidentiality. The information provided by Medarex, Inc. to the Investigator may not be disclosed to others without direct written authorization from Medarex, Inc., except to the extent necessary to obtain informed consent from subjects who wish to participate in the trial.

15.8. Discontinuation of Study

Medarex, Inc. reserves the right to discontinue any study for administrative reasons at any time. If appropriate, reimbursement for reasonable expenses will be made.

15.9. Data Management

15.9.1. Data Collection

Investigators must enter the information required by the protocol onto the Medarex, Inc. CRFs that are printed on "no carbon required" (NCR) paper. Medarex, Inc. monitors or

designees will review the CRFs for completeness and accuracy, and instruct site personnel to make any required corrections or additions. The CRFs will be forwarded to the Medarex, Inc. or its designee, with one copy retained at the investigational site. Receipt of CRFs will be recorded by Medarex, Inc. or its designee; the original copy will be placed in Central Trial Master Files and the NCR copy will be forwarded to the responsible Medarex, Inc. data management staff or its designee for processing.

15.9.2. Database Management and Quality Control

Data items from the CRFs will be entered into the study database using double data entry with verifications.

Subsequently, the information entered into the database will be systematically checked by Data Management staff following Medarex, Inc. or its designee data management procedures. Obvious errors will be corrected by Medarex, Inc. or its designee personnel. Other errors, omissions, or requests for clarification will be queried; queries will be returned to the investigational site for resolution. A copy of the signed Data Clarification Form will be kept with the CRFs, and once the original is received, the resolutions will be entered into the database. Quality control audits of all key safety and activity data in the database will be made after entering data from each visit.

Concomitant medications and prior antiretroviral therapy entered into the database will be coded using a WHODRL dictionary. Coexistent medical conditions, adverse events and other medical events will be coded using MedDRA dictionary.

When the database has been declared to be complete and accurate, the database will be locked. Any changes to the database after that time can only be made by joint written agreement of the Medarex, Inc. study team.

16. REFERENCES

1. Bucy RP. Approach to HIV antigen specific immune enhancement. In: Dolin R, Masur H, Saag MS, editors. AIDS Therapy (2nd Edition). New York: Churchill Livingstone, 2003 (in press).
2. O'Connor DH, Allen TM, Vogel TU, Jing P, DeSouza IP, Dodds E, et al. Acute phase cytotoxic T lymphocyte escape is a hallmark of simian immunodeficiency virus infection. Nat Med 2002;8:493-499.
3. Shankar P, Russo M, Harnisch B, Patterson M, Skolnik P, Lieberman J. Impaired function of circulating HIV-specific CD8(+) T cells in chronic human immunodeficiency virus infection. Blood 2000;96:3094-3101.
4. Valdez H, Connick E, Smith KY, Lederman MM, Bosch RJ, Kim RS, et al. Limited immune restoration after 3 years' suppression of HIV-1 replication in subjects with moderately advanced disease. Aids 2002;16:1859-1866.
5. Rosenberg ES, Billingsley JM, Caliendo AM, Boswell SL, Sax PE, Kalams SA, et al. Vigorous HIV-1-specific CD4+ T cell responses associated with control of viremia. Science 1997;278:1447-1450.
6. Pitcher CJ, Quittner C, Peterson DM Connors M, Koup RA, Maino VC, et al. HIV-1-specific CD4+ T cells are detectable in most individuals with active HIV-1 infection, but decline with prolonged viral suppression. Nat Med 1999;5:518-525.
7. Leng Q, Bentwich Z, Magen E, Kalinkovich A, Borkow G. CTLA-4 upregulation during HIV infection: association with anergy and possible target for therapeutic intervention. Aids 2002;16:519-529.
8. Steiner K, Waase I, Rau T, Dietrich M, Fleischer B, Broker BM. Enhanced expression of CTLA-4 (CD152) on CD4+ T cells in HIV infection. Clin Exp Immunol 1999;115:451-457.
9. Miranda LR, Schaefer BC, Kupfer A, Hu Z, Franzusoff A. Cell surface expression of the HIV-1 envelope glycoproteins is directed from intracellular CTLA-4-containing regulated secretory granules. Proc Natl Acad Sci USA 2002;99:8031-8036.
10. Riley JL, Schlienger K, Blair PJ, Carreno B, Craighead N, Kim D, et al. Modulation of susceptibility to HIV-1 infection by the cytotoxic T lymphocyte antigen 4 costimulatory molecule. J Exp Med 2000;191:1987-1997.
11. Lenschow DJ, Walunas TL, Bluestone JA. CD28/B7 system of T cell costimulation. Ann Rev Immunol 1996;14:233-258.
12. Schwartz RH. Costimulation of T lymphocytes: the role of CD28, CTLA4, and B7/BB1 in interleukin-2 production and immunotherapy. Cell 1992;71(7):1065-8.
13. Linsley PS, Brady W, Urnes M, Grosmaire LS, Damle NK, Ledbetter JA. CTLA-4 is a second receptor for the B cell activation antigen B7. J Exp Med 1991;174(3):561-9.
14. Egen JG, Kuhns MS, Allison JP. CTLA-4: new insights into its biological function and use in tumor immunotherapy. Nat Immunol 2002;3:611-618.
15. Thompson CB, Allison JP. The emerging role of CTLA-4 as an immune attenuator. Immunity 1997;7(4):445-50.

16. Kearney ER, Walunas TL Karr RW, et al. Antigen-dependent clonal expansion of a trace population of antigen- specific CD4+ T cells in vivo is dependent on CD28 costimulation and inhibited by CTLA-4. J Immunol 1995;155(3):1032-6.

17. Krummel MF, Allison JP. CD28 and CTLA-4 have opposing effects on the response of T cells to stimulation. J Exp Med 1995;182(2):459-65.

18. Krummel MF, Sullivan TJ, Allison JP. Superantigen responses and co-stimulation: CD28 and CTLA-4 have opposing effects on T cell expansion in vitro and in vivo. Int Immunol 1996;8(4):519-23.

19. Walunas TL, Lenschow DJ, Bakker CY, et al. CTLA-4 can function as a negative regulator of T cell activation. Immunity 1994;1(5):405-13.

20. Chambers CA, Sullivan TJ, Allison JP. Lymphoproliferation in CTLA-4-deficient mice is mediated by costimulation-dependent activation of CD4+ T cells. Immunity 1997;7(6):885-95.

21. Tivol EA, Borriello F, Schweitzer AN, Lynch WP, Bluestone JA, Sharpe AH. Loss of CTLA-4 leads to massive lymphoproliferation and fatal multiorgan tissue destruction, revealing a critical negative regulatory role of CTLA-4. Immunity 1995;3(5):541-7.

22. Waterhouse P, Penninger JM, Timms E, et al. Lymphoproliferative disorders with early lethality in mice deficient in CTLA-4. Science 1995;270(5238):985-8.

23. Tchekmedyian S, Glasby J, Korman A, Keler T, Deo Y, Davis TA. MDX-010 (human anti-CTLA4): a phase I trial in malignant melanoma. Proc ASCO;21:15a.

24. Davis TA, Tchekmedyian S, Korman A, Keler T, Deo Y, Small EJ. MDX-010 (human anti-CLTA4): a phase I trial in hormone refractory prostate cancer. Proc ASCO;21:19a.

25. Basara N, Kiehl MG, Fauser AA. New therapeutic modalities in the treatment of graft-versus-host disease. Crit Rev Oncol Hematol 2001;38:129-38.

26. Deeks SG, Wrin T, Liegler T, Hoh R, Hayden M, Barbour JD, et al. Virologic and immunologic consequences of discontinuing combination antiretroviral-drug therapy in HIV-infected subjects with detectable viremia. N Engl J Med 2001;344:472-480.

27. Stanley S, Ostrowski MA, Justement JS, Gantt K, Hedayati S, Mannix M, et al. Effect of immunization with a common recall antigen on viral expression in subjects infected with human immunodeficiency virus type 1. N Engl J Med 1996;334:1222-1230.

17. APPENDICES

Appendix 1: Instructions for the Handling, Storage, and Shipment of Blood Samples

Plasma HAHA Analysis

Blood samples (2 mL) for evidence of a host anti-idiotype antibody titer (subject-derived antibodies binding specifically to MDX-010) will be drawn into heparin tubes from a forearm vein in the contra-lateral arm to which the MDX-010 is infused.

Immediately after each tube of blood is drawn, it should be inverted gently several times to insure the mixing of contents (e.g., anticoagulant). Prolonged sample contact with the rubber stopper should be avoided. The tube should be placed upright in a test tube rack at room temperature until centrifugation. Within 10 minutes, the sample should be centrifuged between 3°C and 5°C for five minutes at approximately 2200 g. All plasma should be transferred to a polypropylene screw-cap tube and freeze the samples within 25 minutes of blood collection. Tubes will be labeled as follows: Protocol No., Center No., Subject Number, Sample No., Interval, Date and Time drawn. The tubes will be kept frozen at ≤-20°C pending analysis.

A semi-quantitative enzyme-linked immunosorbant assay (ELISA) will be used to detect IgG antibodies to MDX-010 in the plasma samples. In this assay, plates are coated with MDX-010 F(ab')$_2$ and then antibodies against MDX-010 idiotype are detected with an anti-human IgG, Fc specific conjugated probe. The results for each sample are expressed as fold increase in titer relative to a pretreatment sample.

Pharmacokinetic Assessment

Pharmacokinetic blood sample collection and handling

Blood samples (2 mL) for the analysis of plasma concentrations of MDX-010 will be drawn according to the schedule listed in Table 1. Samples will be drawn from a contra-lateral forearm vein of the MDX-010 infusion into heparin tubes.

Immediately after each tube of blood is drawn, it will be inverted gently several times to insure the mixing of contents (e.g., anticoagulant). Prolonged sample contact with the rubber stopper should be avoided. The tube will be upright in a test tube rack at room temperature until centrifugation. Within 10 minutes, the sample will be centrifuged between 3°C and 5°C for 5 minutes at approximately 2200 g. All of the plasma will be transferred to a polypropylene screw-cap tube and frozen within 25 minutes of blood collection. Tubes will be labeled as follows: Protocol No., Center No., Subject Number, Sample No., Interval, Date, Visit Number, and Time Drawn. The tubes will be kept frozen at ≤-20°C pending analysis.

The Medarex, Inc. laboratory will perform the plasma MDX-010 levels and HAFPA assays at their facility in Bloomsbury, NJ.

It is important to record the exact date and sampling time on the blood collection record. Tubes are to be labeled with the following information:

Protocol number , Site number, Subject number, Subject initials, Sample number and

Date (DD/MMM/YYYY) and actual time (24 hour clock) the blood sample was drawn

Immunologic Samples

Blood samples for the analysis of Special Immune Function Measurements will be drawn according to the schedule listed in Table 1. The amounts and tubes required for each test are:

T cell subsets - flow panel	1 x 5 ml purple
LPA panel	1 x 10 ml yellow (ACD)
Cytokine Production (EIA)	2 x 10 ml yellow
Stored cryo PBMC	6 x 10ml yellow
ICC-IHC	will be done from cyro PBMC

Immediately after each tube of blood is drawn, it will be inverted gently several times to insure the mixing of contents (e.g., anticoagulant). Prolonged sample contact with the rubber stopper should be avoided. The tube will be upright in a test tube rack at room temperature.

It is important to record the exact date and sampling time on the blood collection record. Tubes are to be labeled with the following information:

Protocol number , Site number, Subject number, Subject initials, Sample number and

Date (DD/MMM/YYYY) and actual time (24 hour clock) the blood sample was drawn

Whole blood samples will be shipped overnight at room temperature to the following address for analysis:

[redacted]

Notification of sample shipment is to be faxed to [redacted]

Results of standard hematology test from central lab obtained on the same day are also to be faxed to [redacted] as soon as available.

Biopsy Samples

Any biopsy specimens obtained in the workup of an adverse event (eg diarrhea) should include 3 to 5 biopsies embedded in paraffin. 5 to 7 cuts from each paraffin block should be made (in addition to those required by the local pathologist) and sent by standard packaging to the consulting immunopathologist for the study.

It is important to record the exact date and sampling time on the specimens, and should include the following information:

Protocol number , Site number, Subject number, Subject initials, Sample number and

Date (DD/MMM/YYYY) and actual time (24 hour clock) the biopsy sample was drawn

Specimens should be shipped to:

[redacted]ngham
Birmingham, AL 35249-7331

Notification of sample shipment is to be faxed to [redacted]

All Other Samples

All samples other than those listed above or those identified to be analyzed at the investigational site will be handled, stored and shipped according to the instructions given in the laboratory instructional manual.
